# A New Horizon: Expanding the Access and Impact of Psychosocial Oncology—8–9 June 2026, 41st Annual CAPO Conference

**DOI:** 10.3390/curroncol33080443

**Published:** 2026-07-23

**Authors:** Peter Traversa, Sheila Garland

**Affiliations:** Canadian Association of Psychosocial Oncology, 189 Queen St E., Suite 1, Toronto, ON M5A 1S2, Canada

**Keywords:** psychosocial, oncology, cancer, research, accessible, sustainable, innovation

## Abstract

On behalf of the Canadian Association of Psychosocial Oncology, we are pleased to present the abstracts from the 2026 Annual Conference, titled “A New Horizon: Expanding the Access and Impact of Psychosocial Oncology”. The 41st Annual CAPO Conference was held in St. John’s, Newfoundland from 8 June 2026 to 9 June 2026. As we stand at a new horizon in psychosocial oncology, we recognize the unprecedented opportunities to expand both the access to and the impact of comprehensive cancer care. This conference will explore innovative strategies for breaking down traditional barriers that have historically limited access to psychosocial support, including geographic isolation, resource constraints, cultural disparities, and systemic inequities in healthcare delivery. This expansion of reach and influence represents not merely growth in service numbers, but a fundamental transformation in how we conceptualize, design, and implement patient-centered psychosocial care across diverse communities and care settings. We will explore scalable solutions that amplify impact while maintaining the deeply personal, human-centered approach that defines excellence in psychosocial oncology. From telehealth innovations and peer support networks to community-based interventions and integrated care models, this conference will showcase evidence-based strategies that expand our collective ability to support individuals and families navigating the cancer journey, wherever they may be. This conference brought together key stakeholders including multidisciplinary professionals from nursing, psychology, psychiatry, social work, spiritual care, nutrition, medicine, rehabilitation medicine, occupational health and radiation therapy for both adult and pediatric populations. Participants included clinicians, researchers, educators in cancer care, community-based organizations and patient representatives. Patients, caregivers and family members presented abstracts that speak to their role in managing cancer experiences and care. Over one-hundred and fifty (150) abstracts were submitted for presentation as symposia, 20 min oral presentations, 10 min oral presentations, 90 min workshops and poster presentations. We congratulate all the presenters on their research work and contributions.

## Simple Summary

Psychosocial oncology addresses the emotional, social, and practical needs of people living with cancer, alongside their medical treatment. Despite growing evidence that this kind of support improves quality of life, many patients still cannot access it because of where they live, limited health system resources, cultural barriers, or gaps in care delivery. The Canadian Association of Psychosocial Oncology held its 41st Annual Conference in St. John’s, Newfoundland and Labrador, in June 2026, bringing together clinicians, researchers, patients, and caregivers from many health professions. Over 150 abstracts were presented, covering topics such as digital health tools, community-based care, equity, and support across all ages, from children to older adults. This report summarizes the conference program and the abstracts shared. Together, this work points to practical, scalable ways to expand access to psychosocial cancer care and can help guide future research, inform health policy, and support programs that reach more patients and families, including those in remote or under-resourced communities.

## 1. Abstract Themes

A. Adapting PSO care in LMI countries.

B. Cancer care across the life span (children, adolescent and young adults, adults, and older adults).

C. Community-based and volunteer cancer care services.

D. Complementary and integrative cancer care.

E. Exercise/pre-habilitation and rehabilitation in cancer.

F. Equity, diversity and inclusion (sociodemographic, culture, and sex/gender issues).

G. Healthcare provider wellness.

H. High tech to high touch (digital health, value-based care, integrated PSO interventions).

I. Implementation science, knowledge translation and synthesis.

J. Palliative and end-of-life care.

K. Pandemics and cancer care issues.

L. Patient-oriented research approaches.

M. Primary, secondary and tertiary cancer prevention.

N. Novel interventions and clinical trials in PSO.

O. Survivorship.

P. Other.

## 2. Symposia

### 2.1. S1—From Evidence to Implementation: CALM and Adaptations Across the Cancer Continuum

**Moderator:** Gary Rodin.
**Summary**

Aligned with the CAPO 2026 theme of expanding access and impact in psychosocial oncology, this symposium highlights innovative strategies to deliver evidence-based interventions across diverse populations and care settings. We focus on Managing Cancer and Living Meaningfully (CALM), a brief, manualized psychotherapy for patients with advanced cancer, and its adaptations for unique contexts.

First, we present the Global CALM Program, which trains clinicians worldwide using scalable models and lessons from culturally diverse, resource-limited settings. Second, we describe the CALM National Program, an implementation and quality improvement initiative designed to embed CALM as a routine and proactive component of cancer care across Canadian centres. Third, we share findings from the CALM Ovarian Feasibility Study, examining the acceptability and impact of offering CALM at diagnosis and recurrence, including qualitative insights into patient experiences. Finally, we present preliminary qualitative results from a randomized trial of Emotion and Symptom-Focused Engagement (EASE), a trauma-focused adaptation of CALM for parents of children with cancer, illustrating how CALM principles address parental distress and trauma.

Together, these presentations demonstrate how CALM and its adaptations overcome barriers to psychosocial care through global dissemination, national implementation, and population-specific innovations, creating new horizons for patient-centred support across the cancer continuum.

### 2.2. S1—19: Building Systems for CALM Across Canada: A National Program Driving Sustainability and Equity

Laura Foran, Kathryn Hunt and Gary Rodin.
Princess Margaret Cancer Centre, Toronto, Canada.

**Background/Rationale or Objectives/Purpose**

A diagnosis of advanced cancer is often accompanied by significant psychological distress, including depression, demoralization and fears about death and dying. Managing Cancer and Living Meaningfully (CALM) is a brief, evidence-based psychotherapy designed to address these psychological and existential challenges. While efficacy is well established, the CALM National program was developed to ensure equitable access and system-wide impact.

**Methodology or Methods**

Drawing on the Plan–Do–Study–Act (PDSA) model and informed by Normalization Process Theory (NPT), the CALM National Program is organized into three temporal phases, each with concrete and measurable goals. Phase I embedded CALM as routine practice at the Princess Margaret Cancer Centre (PM), while developing education and training tools for broader dissemination. Phase II expands implementation to partner sites, identifying champions, initiating training, and adapting processes for local contexts. Phase III will focus on national scale-up and sustainability.

**Impact on Practice or Results**

At PM, 14 multidisciplinary CALM therapists are certified, with 11 more in training, across psychiatry, psychology, nursing, palliative care, and social work. Refined processes are in use across five tumour sites, and new patients are streamlined through a dedicated referral pathway. To support national scaling, a virtual training platform was launched on Cancer Campus in March 2025. Seven partner sites have committed to training and referral integration, with additional equity-focused partnerships under development.

**Discussion or Conclusions**

Key lessons in implementation include the importance of early co-design, oncologist endorsement, and advocacy for flexible staffing models. Future directions include targeted implementation studies, AI-enhanced certification, expanded supervision, and establishing a national Community of Practice.

### 2.3. S1—20: The Global CALM Program: Scaling an Evidence-Based Psycho-Oncology Intervention Across Cultures and Health Systems

Junhee Baek ^1^, Elizaveta Klekovkina ^1^ and Gary Rodin. ^1,2^
^1^ Princess Margaret Cancer Centre, Toronto, Canada;
^2^ Temerty Faculty of Medicine, University of Toronto, Toronto, Canada.

**Background/Rationale or Objectives/Purpose**

Untreated psychological and existential distress in advanced cancer is a global challenge. Managing Cancer and Living Meaningfully (CALM), developed at Princess Margaret Cancer Centre, is a brief, evidence-based psychotherapy, grounded in relational and attachment theory and mentalization-based therapy. CALM has demonstrated efficacy in randomized trials, reducing depression and death anxiety, and improving end-of-life preparation.

**Methodology or Methods**

The Global CALM Program promotes accessibility and scalability through international dissemination, clinician training, and research collaborations. A network of regional hubs across Europe, Austral-Asia, Africa, and the Americas supports training and data collection. Dissemination strategies include multilingual global workshops, online educational modules for flexible learning, and virtual clinical supervision to overcome geographic barriers. Training combines standardized curricula with cultural adaptations. Research initiatives and Global CALM Leadership Summits foster international networking and knowledge exchange, strengthening the CALM community worldwide.

**Impact on Practice or Results**

CALM has been implemented in over 20 countries and adapted for diverse cultural and resource settings. In lower-resourced contexts, adaptations have prioritized feasibility, local clinician training, and alignment with existing palliative care structures. In higher-resourced contexts, implementation has emphasized advanced training pathways and integration into established psycho-oncology services. These tailored strategies have strengthened psychosocial care capacity globally.

**Discussion or Conclusions**

Lessons learned highlight CALM’s adaptability and cultural resonance. Future directions include expanding regional hubs, integrating CALM into national cancer care guidelines, and leveraging digital platforms for training and supervision to make CALM a global standard of care.

### 2.4. S1—22: Feasibility and Acceptability of Trauma-Focused Managing Cancer and Living Meaningfully (CALM-TF) in Newly Diagnosed and Recurrent Ovarian Cancer: Preliminary Findings of a Qualitative Study

Megan George ^1,2^, Stephanie Lheureux ^1,2^, Amalya Feldman ^2^, Annie Yu ^1^, Lauren Philp ^1,2^ and Gary Rodin ^1,2^
^1^ University of Toronto, Toronto, Canada;
^2^ Princess Margaret Cancer Centre, Toronto, Canada.

**Background/Rationale or Objectives/Purpose**

Ovarian cancer (OC) has the highest mortality rate among gynecological cancers, with >80% of cases detected at an advanced stage. Newly diagnosed (ND-OC) or recurrent (R-OC) ovarian cancer often triggers traumatic stress symptoms (TSSs). The purpose of this study is to evaluate the feasibility and acceptability of trauma-focused Managing Cancer And Living Meaningfully (CALM-TF) services and treatments, initiated at these time points.

**Methodology or Methods**

A phase II single-arm trial of CALM-TF was conducted to test its feasibility and acceptability in patients with ND-OC or R-OC. Semi-structured qualitative interviews were conducted with eleven participants (ND = 4; R = 7) following study completion, and additional interviews are planned. Data was analyzed using interpretive description methodology.

**Impact on Practice or Results**

Findings suggest CALM-TF is feasible, with a recruitment rate of 33.3% at diagnosis and 63% at recurrence of OC; 84.2% of participants completed >3 CALM-TF sessions over 6 months. Participants with ND-OC valued CALM-TF to help them manage the initial shock and anxiety. R-OC participants described CALM-TF helping them process “re-traumatization” and shifting goals of care. Participants at both time points highlighted therapists’ ability to provide concrete coping strategies. All participants advocated for CALM-TF to be a standard of care from the point of diagnosis. Those recruited at recurrence describe “unnecessary suffering” between diagnosis and recurrence.

**Discussion or Conclusions**

The findings of this study support the feasibility and acceptability of CALM-TF for patients with a ND-OC or R-OC. A planned future RCT will determine the efficacy of CALM-TF to relieve TSSs in this population.

### 2.5. S1—40: Emotion and Symptom-Focused Engagement for Parents of Children with Cancer: Preliminary Qualitative Insights from a Phase III RCT Addressing Traumatic Stress

Stephanie Nanos ^1,2,3^, Lindsay Jibb ^1,3^, Juliane Feliciano ^1^, Sarah Alexander ^4,5^, Nhu-An Pham ^2^, David Brownstone ^1^, Laura Foran ^2^, Kate Hunt ^2^, Elizaveta Klekovkina ^2^ and Gary Rodin ^2,3^
^1^ Hospital for Sick Children, Toronto, Canada;
^2^ Princess Margaret Cancer Centre, Toronto, Canada;
^3^ University of Toronto, Toronto, Canada;
^4^ BC Children’s Hospital, Vancouver, Canada;
^5^ University of British Columbia, Vancouver, Canada.

**Background/Rationale or Objectives/Purpose**

Childhood cancer can be profoundly traumatic for parents, who report moderate-to-severe traumatic stress symptoms (TSSs). However, evidence for effective parent-focused psychosocial interventions remains limited. To address these symptoms, we developed Emotion and Symptom-Focused Engagement (EASE), a trauma-focused adaptation of CALM, which we have shown to be feasible and acceptable to parents of children with cancer. We are now conducting a CIHR-funded RCT to determine its efficacy in mitigating TSSs.

**Methodology or Methods**

This multimethod RCT aims to enroll 306 parents at the Hospital for Sick Children. The treatment arm receives usual care plus EASE, delivered over 12 weeks by a trained clinician. The control arm receives usual care alone. A qualitative component is nested within the trial. After 12 weeks, participants may complete semi-structured interviews to explore mechanisms influencing TSSs, and, among those receiving EASE, how they understand and apply the intervention.

**Impact on Practice or Results**

Since February 2025, 46 parents have been randomized. Preliminary qualitative insights indicate that parents’ distress fluctuates with their child’s symptoms and is compounded by sleep disruption, anxiety, and isolation. Parents described EASE as meeting the needs not addressed by other supports by providing relational support and a structured space to process difficult emotions. Participants reported incorporating EASE strategies into daily life and noted improvements in their wellbeing and relationships, advocating for EASE to be offered as standard of care.

**Discussion or Conclusions**

The findings will inform optimization of EASE for parents’ trauma-related needs. If effective, EASE has the potential to be incorporated into the standard of psychosocial care for this population.

### 2.6. S2—Novel Approaches to Patient Navigation in Cancer Care

**Moderator:** Perri Tutelman.
**Summary**

The challenge of navigating complex cancer systems is among the top unmet needs reported by patients diagnosed with cancer in Canada. Patient navigation interventions, which seek to support patients through the cancer care trajectory with personalized assistance and guidance, have emerged as a strategy to overcome barriers to accessing equitable and timely services. While patient navigation interventions have been associated with improvements in patient outcomes, implementation challenges remain such as poor awareness, limited reach, geographic coverage, and uptake, limited use of technology, insufficient system integration, and lack of sustainable funding. Novel approaches to patient navigation in cancer care are urgently needed. This symposium will feature presentations from three groups across Canada (Alberta, Ontario, and Nova Scotia) that are co-designing novel solutions to address current challenges in cancer patient navigation. First, R. Urquhart will describe the co-design of a virtual navigation intervention for people living with advanced cancer in Canada. Second, J. Bender will outline the barriers and facilitators with implementing a digital peer navigation program in prostate cancer survivorship care. Finally, P. Tutelman will discuss the need for and co-design of a digital intervention to support adolescents and young adults with system navigation around the time of diagnosis in Alberta.

### 2.7. S2—48: Co-Designing a Novel Digital Intervention to Support Adolescents and Young Adults with Navigation Around the Time of Diagnosis

Perri Tutelman ^1^, Se’era Anstruther ^1^, Emily Memedovich ^1^, Sara Beattie ^2^, Andrea DeIure ^2^, Megan Hessel ^1^, Dana Male ^2^, Bobbi Michaud ^2^, Fiona Schulte ^1^ and Linda Watson ^2^
^1^ University of Calgary, Calgary, Canada;
^2^ Cancer Care Alberta, Calgary, Canada.

**Background/Rationale or Objectives/Purpose**

Adolescents and young adults (AYAs) diagnosed with cancer have unique needs and face unique challenges accessing supportive care services and supports within the cancer care system. This presentation will outline recent findings from studies that focused on co-designing ANCHOR, a novel digital health intervention to support AYAs with navigation around the time of diagnosis.

**Methodology or Methods**

The methods include the following: (1) needs-assessment focus groups with AYAs and individual interviews with healthcare providers in Alberta to explore the experiences of AYAs around the time of diagnosis and to identify key components of an intervention; (2) co-design workshops with AYAs and healthcare providers to determine the features, functionality, and architecture of the intervention.

**Impact on Practice or Results**

First, an overview of findings of the needs assessment with AYAs (n = 14; 19–39 years, within 2 years of diagnosis) and healthcare providers (n = 10; various disciplines) will be presented, including unmet needs around the time of diagnosis and preferences for an intervention. Next, results from the co-design workshops with AYAs (n = 5; 23–40 years, within 2 years of diagnosis) and healthcare providers (n = 6, various disciplines) will be shared, including participant perspectives on intervention utility, necessary features, and potential challenges.

**Discussion or Conclusions**

This presentation will offer insights into the initial co-design of a digital intervention to support AYAs with navigating the cancer care system around the time of diagnosis. The findings will inform design principles for guiding the next steps in intervention development.

### 2.8. S2—64: Barriers and Facilitators to Implementing a Digital Peer Navigation Program in Routine Prostate Cancer Survivorship Care

Jacqueline Bender ^1^, Sarah Scruton ^1,2^, Logan Meyers ^1^, Robin Urquhart ^2,3^, Arminee Kazanjian ^4^, Andrew Matthew ^1^ and Jennifer Jones ^1^
^1^ Princess Margaret Cancer Centre, University Health Network, Toronto, Canada;
^2^ Nova Scotia Health Authority, Halifax, Canada;
^3^ Dalhousie University, Halifax, Canada;
^4^ School of Population and Public Health, University of British Columbia, Vancouver, Canada.

**Background/Rationale or Objectives/Purpose**

PeerNav is a digital peer navigation program that aims to address gaps in care, including lack of access to adequate information and emotional support after treatment. This qualitative study evaluated the barriers and facilitators to implementing PeerNav into routine survivorship care for prostate cancer patients.

**Methodology or Methods**

In-depth virtual interviews were conducted with 13 healthcare professionals and administrators at cancer centres in Ontario, British Columbia, and Nova Scotia. Data collection and analysis were informed by the Consolidated Framework for Implementation Research to identify determinants of implementation success.

**Impact on Practice or Results**

The participants identified a clear need for PeerNav, describing it as a unique intervention that fills gaps in supportive care and aligns with the priorities and goals of each institution. Additional facilitators included provider buy-in and committed clinical champions, comprehensive training and support for navigators, and partnerships within and outside of organizations. Barriers included a lack of referrals due to limited awareness and competing clinical demands, constrained resources and prioritization of cancer control over supportive care, inconsistent care pathways, and varying technology proficiency. Strategies to optimize implementation involved targeted education for patients and providers, seamless referral processes embedded into existing workflows and infrastructure, partnerships with organizations and clinical champions, and adaptation to local contexts.

**Discussion or Conclusions**

This study identified the key conditions for successful integration of PeerNav into oncology workflows. Addressing the identified barriers and leveraging facilitators will guide system-wide implementation of the program, ensuring it is a part of routine care for prostate cancer patients.

### 2.9. S2—98: Co-Designing a Navigational Intervention to Meet the Needs of People Living Long-Term with Advanced Cancer

Robin Urquhart ^1^, Sarah Scruton ^2^, Cynthia Kendell ^1^, Julee Pauling ^3^, Yelena Aizenberg ^4^, Amy Trottier ^1^, Alison Wallace ^1^, Tony Reiman ^5^ and John Thoms ^6^
^1^ Dalhousie University, Halifax, Canada;
^2^ Nova Scotia Health, Halifax, Canada;
^3^ Patient Partner, Ottawa, Canada;
^4^ Patient Partner, Toronto, Canada;
^5^ Dalhousie University, Saint John, Canada;
^6^ Memorial University of Newfoundland, St. John’s, Canada.

**Background/Rationale or Objectives/Purpose**

Due largely to advances in novel therapies, there is a growing population of Canadians with advanced cancer who are living long-term with their disease. We undertook qualitative interviews to understand the experiences and needs of this growing patient population. Informed by what we learned, we sought to co-design an intervention to address their needs and support their health and wellbeing while living with their disease.

**Methodology or Methods**

**Sample:** Adults from Canada living long-term with advanced cancer, their healthcare providers, and researchers with methodologic and supportive care expertise.

**Procedures:** A patient survey to prioritize interventions, followed by three online co-design workshops.

**Impact on Practice or Results**

The survey prioritized five broad areas to bring forward to the co-design workshops: one-stop informational resource, healthy living interventions, tailored mental health services, financial planning/support, and caregiver/family support. During Workshop #1 (patients only), discussions focused on understanding these areas, with attendees identifying key touchpoints and opportunities to improve. The resultant concept was a patient navigation/informational resource to help patients access the many resources and supports they need from the time of diagnosis. Workshop #2 (providers/researchers) focused on further shaping a navigation/informational intervention, including feasibility and implementation considerations. Workshop #3 (all groups) delineated core components and implementation considerations for a navigation/informational intervention, tailored specifically for individuals living with advanced cancer.

**Discussion or Conclusions**

Co-design offers an opportunity to design programs better suited to meeting the unique needs of patients. A virtual navigation intervention, using a stepped care approach that maximizes accessibility principles, is now being developed for future testing.

### 2.10. S3—It Takes a Village: Advancing FORT from Efficacy to National Implementation and Adaptation for Survivors of Childhood Cancer

**Moderator:** Sophie Lebel.
**Summary**

The fear of cancer recurrence (FCR) is the most frequent unmet need of cancer survivors. The Fear of Recurrence Therapy (FORT) efficaciously reduces FCR for survivors of adult cancers. We recently began moving FORT beyond efficacy trials to reach the patients it was designed to support. We completed an implementation study of FORT in five Canadian cancer centers, examining barriers and facilitators to adoption and maintenance, partnered with a community organization to offer FORT to survivors in smaller communities who may not otherwise have access, and began work on adapting FORT for survivors of childhood cancer, who have unique developmental needs, but among whom one in three report clinically significant FCR. This work aligns with Canada’s Strategy for Patient-Oriented Research, which emphasizes collaboration among researchers, healthcare professionals, patients, and community partners to build sustainable and equitable health care.

First, Dr. Maheu and colleagues will present FORT implementation results from three sites. Second, Ms. Smart will share results from a pilot of an online community-based implementation. Last, Dr. Russell and two patient-partners will describe adapting FORT for adolescent and early young adult survivors of childhood cancer. Directions for future research and implementation of FCR care with diverse populations will be discussed.

### 2.11. S3—62: From Research to Community: Delivering Fear of Recurrence Therapy at Wellspring

Amanda Smart.
Wellspring Cancer Support Foundation, Toronto, Canada.

**Background/Rationale or Objectives/Purpose**

Fear of cancer recurrence (FCR) is a prevalent and persistent concern among cancer survivors, characterized by excessive worry, hypervigilance to bodily symptoms, and intrusive thoughts [1]. Left unaddressed, FCR can significantly diminish quality of life and contribute to anxiety, fatigue, and maladaptive coping [2]. Although evidence-based interventions exist, such programs are often limited to research or clinical settings.

**Methodology or Methods**

Wellspring Cancer Support Foundation (WCSF), a national not-for-profit organization providing no-cost psychosocial cancer support, partnered with Drs. Sophie Lebel and Christine Maheu to pilot Fear of Recurrence Therapy (FORT) within a community setting [3,4]. Two concurrent, six-week group sessions were delivered between October and December 2025. Trained program leaders conducted pre-program screening interviews with 25 participants to assess FCR severity and program suitability. Sessions followed the standardized FORT curriculum, combining psychoeducation, cognitive–behavioural strategies, and group discussion, with weekly homework assignments to reinforce skill development. Post-pilot evaluations thus far have indicated 100% participant satisfaction. Participants reported increased awareness of FCR triggers, improved ability to manage fear, provided practical coping strategies, and high perceived relevance to survivorship needs.

**Impact on Practice or Results**

By embedding FORT in a community-based cancer support organization, this pilot expanded access to an evidence-based FCR intervention outside academic and clinical institutions, offering a scalable and supportive model for broader survivor populations.

**Discussion or Conclusions**

This initiative represents a novel translation of FORT from research to community care. Early lessons highlight the importance of facilitator training, participant screening, and integration with existing psychosocial programming. The findings from the pilot will inform future program refinement, evaluation, and national scaling of FCR interventions at Wellspring.

**References**

1. Luigjes-Huizer YL, Tauber NM, Humphris G, Kasparian NA, Lam WWT, Lebel S, Simard S, Smith AB, Zachariae R, Afiyanti Y, Bell KJL, Custers JAE, de Wit NJ, Fisher PL, Galica J, Garland SN, Helsper CW, Jeppesen MM, Liu J, Mititelu R, Monninkhof EM, Russell L, Savard J, Speckens AEM, van Helmondt SJ, Vatandoust S, Zdenkowski N, van der Lee ML. What is the prevalence of fear of cancer recurrence in cancer survivors and patients? A systematic review and individual participant data meta-analysis. Psychooncology. 2022 Jun;31(6):879–892. doi: 10.1002/pon.5921. Epub 2022 Apr 7. PMID: 35388525; PMCID: PMC9321869.
2. Bergerot CD, Philip EJ, Bergerot PG, Siddiq N, Tinianov S, Lustberg M. Fear of Cancer Recurrence or Progression: What Is It and What Can We Do About It? Am Soc Clin Oncol Educ Book. 2022 Apr; 42:1–10. doi: 10.1200/EDBK_100031. PMID: 35561298.
3. Christine Maheu, Sophie Lebel, Lori J. Bernstein, Christine Courbasson, Mina Singh, Sarah E. Ferguson, Cheryl Harris, Lynne Jolicoeur, Lorena Baku, Linda Muraca, Agnihotram V. Ramanakumar, Frederic Lamonde, Monique Lefebvre, Christina Tomei, Brittany Mutsaers, Scott Secord, Joanne Power, Nancy Drummond, Maude Hébert, Rajvi J. Wani. (2023). Fear of Cancer Recurrence Therapy (FORT): A Randomized Controlled Trial. Health Psychology, Volume 42 (Issue 3 Mar), 182–194. https://psycnet.apa.org/fulltext/2023-49863-005.html (accessed on 1 January 2026).
4. Lebel, S., Maheu, C., Lefebvre, M., Secord, S., Courbasson, C., Singh, M., Jolicoeur, L., Benea, A., Harris, C., Fung, M. F. K., Rosberger, Z., and Catton, P. (2014). Addressing fear of cancer recurrence among women with cancer: A feasibility and preliminary outcome study. Journal of Cancer Survivorship, 8(3), 485–496.

### 2.12. S3—107: Fear of Cancer Recurrence Therapy (FORT): Analysis of Post-Implementation Facilitators and Barriers from Canadian Centres Sites

Christine Maheu ^1^, Samara Perez ^2^, Sara Beattie ^3^, Elaine Holden ^4^, Paula Newhook ^4^, Aurélie Guindon ^5^ and Sophie Lebel ^5^
^1^ McGill University, Montreal, Canada;
^2^ McGill University Health Centre, Montreal, Canada;
^3^ Arthur J.E. Child Comprehensive Cancer Centre, Calgary, Canada;
^4^ NL Health Services, St. John’s, Canada;
^5^ University of Ottawa, Ottawa, Canada.

**Background/Rationale or Objectives/Purpose**

Fear of cancer recurrence (FCR) is a common yet often unaddressed concern among cancer survivors. The Fear of Recurrence Therapy (FORT), a structured six-session group intervention, has demonstrated efficacy across two randomized controlled trials. FORT has since been implemented at five Canadian cancer centres. The objective of this study was to examine post-implementation facilitators and barriers influencing the sustainability of FORT across these sites.

**Methodology or Methods**

Across participating centres, manager engagement ranged from three to five individuals per site. Each site delivered two FORT groups, typically involving 6–12 cancer survivors, facilitated by 2–4 trained group leaders. All individuals involved in the implementation—including managers, group leaders, and survivors—were invited to participate in semi-structured video conference interviews. Interviews were recorded, transcribed, and analyzed using deductive thematic analysis guided by the Consolidated Framework for Implementation Research (CFIR). Coding was conducted within an NVivo 15 CFIR-based matrix by a trained analysis team that met regularly to review coding outputs and ensure intercoder reliability.

**Impact on practice or Result**

The preliminary findings suggest that positive survivor feedback and strong clinician motivation contributed to the successful implementation of FORT. The key barriers included constrained resources, misalignment with workflow processes (e.g., FCR screening timelines), and perceptions of intervention complexity.

**Discussion or Conclusions**

Identifying the determinants that promote or hinder the sustainability of FORT will help inform targeted implementation strategies to support the long-term integration of similar therapeutic programs across diverse Canadian cancer care settings.

### 2.13. S3—101: The Fear of Cancer Recurrence Therapy for Adolescent and Early Young Adult Survivors of Childhood Cancer (FORT-AeYA): Adaptation Process and Engagement of Youth Partners

K. Brooke Russell ^1^, Tegan Lyster ^2^, Alia Johnston ^3^, Paige Tough ^1^, Celeste Holy ^1^, Sara Beattie ^4^, Gregory Guilcher ^5,6^, Nicole Racine ^1,7^, Kathleen Reynolds ^5,6^, Fiona Schulte ^5,6^, Perri Tutelman ^5^ and Sophie Lebel ^1^
^1^ University of Ottawa, Ottawa, Canada;
^2^ Patient Partner, Saskatoon, Canada;
^3^ Patient Partner, Victoria, Canada;
^4^ Arthur J.E. Child Comprehensive Cancer Centre, Calgary, Canada;
^5^ University of Calgary, Calgary, Canada;
^6^ Alberta Children’s Hospital, Calgary, Canada;
^7^ Children’s Hospital of Eastern Ontario, Ottawa, Canada.

**Background/Rationale or Objectives/Purpose**

Fear of cancer recurrence (FCR) is the persistent fear of disease progression, relapse, or new malignancies. For survivors of childhood cancer, FCR begins to emerge around ages 13–25. These adolescent and early young adults (AeYAs) may be especially vulnerable to FCR due to their unique cognitive and social-emotional development. The Fear of Cancer Recurrence Therapy (FORT), an evidence-based group therapy for adults, reduces FCR and improves quality of life. Our study adapts FORT for AeYAs (FORT-AeYA) through co-design to optimize content and language for this group.

**Methodology or Methods**

We recruited a diverse group of seven AeYA patient partners to support the initial adaptation. Following the ADAPT framework, adaptations to the intervention protocol were led by our patient partners and focused on ensuring content relevance and developmental appropriateness. Each patient partner contributed their top three suggested changes to every session, which were discussed as a group. Changes were iteratively refined by the study lead and patient partners, tracked using the Framework for Reporting Adaptations and Modifications—Enhanced.

**Impact on Practice or Results**

FORT-AeYA addresses a critical gap in psychosocial care by adapting an established intervention to meet the unique FCR needs of AeYAs. The findings from the adaptation phase will form the foundation for a targeted clinical support for this population.

**Discussion or Conclusions**

This presentation will describe the FORT-AeYA adaptation process, key lessons learned, and perspectives from two AeYA patient partners attending CAPO. The findings will guide the next phase of usability testing and pilot feasibility evaluation, with the long-term goal of improving psychosocial outcomes for AeYAs.

### 2.14. S4—Digital Pathways to Psychological and Functional Recovery in Prostate Cancer Survivorship: Mediation, Behaviour Change, and Global Implementation

**Moderator:** Gabriela Ilie.
**Summary**

Prostate cancer survivors frequently experience psychological distress, weight gain, and urinary and sexual dysfunction, yet the mechanisms driving recovery remain poorly understood. This symposium presents new evidence from three mediation analyses within the Prostate Cancer Patient Empowerment Program (PC-PEP) randomized trial, alongside emerging findings from the global Phase 4 implementation study.

The first presentation demonstrates that adherence to self-monitoring—not pelvic floor exercise duration—mediates improvements in urinary continence, underscoring the importance of digital engagement in functional recovery. The second presentation reveals that improvements in urinary and sexual function do not mediate reductions in psychological distress, challenging long-held assumptions that mental health outcomes are primarily symptom-driven. The third presentation shows that changes in diet quality and physical activity partially mediate PC-PEP-related weight loss, highlighting the program’s multidomain behavioural pathways.

The final presentation from the international Phase 4 trial showcases PC-PEP’s real-world reach across Canada, New Zealand, South Africa, and soon Europe, with participants reporting high satisfaction, strong adherence, and meaningful psychosocial benefits.

Together, these findings illustrate how digital survivorship programs can activate behavioural, psychological, and functional pathways of recovery, offering scalable, equitable models of psychosocial oncology care in diverse settings.

### 2.15. S4—104: Mechanisms of Psychological and Functional Recovery in a Digital Prostate Cancer Survivorship Program: A Dual Mediation Analysis

Gabriela Ilie ^1^, Nathan Smith ^2^, Rob Rutledge ^1^, Emily Chedrawe ^2^, Budoor Salman ^2^, Ricardo Rendon ^2^, Ross Mason ^2^, Andrea Kokorovic ^2^, Nikhilesh Patil ^2^, David Bowes ^2^, Greg Bailly ^2^, Eva Abou-Samra ^2^, Alice Bourne ^2^, Liam-Patrick Hagerman ^2^ and Cody MacDonald ^1^
^1^ Faculty of Medicine, Dalhousie University, Halifax, Canada;
^2^ Dalhousie University, Halifax, Canada.

**Background/Rationale or Objectives/Purpose**

The Prostate Cancer Patient Empowerment Program (PC-PEP) is a 6-month digital survivorship intervention integrating pelvic floor training, lifestyle modification, and psychosocial support. Although PC-PEP improves urinary, sexual, and mental health outcomes, the behavioural and functional mechanisms driving these improvements remain unclear. This study combines two mediation analyses to examine (1) whether self-monitoring and pelvic floor engagement mediate urinary recovery, and (2) whether urinary or sexual function mediate reductions in psychological distress.

**Methodology or Methods**

In a randomized controlled trial, 128 men with localized prostate cancer were assigned to PC-PEP or standard care. Weekly adherence surveys, pelvic floor training duration, urinary function (EPIC), sexual function (EPIC), and psychological distress (K10) were assessed. Mediation models adjusted for demographic, clinical, and treatment variables.

**Impact on Practice or Results**

PC-PEP produced a large improvement in EPIC urinary incontinence scores (mean difference +13.1 points, *p* < 0.001). Self-monitoring adherence significantly mediated 41% of this effect (indirect β = 0.21, *p* < 0.001), whereas pelvic floor training duration did not mediate outcomes (*p* = 0.72). PC-PEP also improved sexual function postoperatively (mean +9.4 points), yet the changes in urinary or sexual function did *not* mediate reductions in psychological distress (K10 Δ = −8.7 points, *p* < 0.001).

**Discussion or Conclusions**

Functional and psychological recovery in prostate cancer survivorship arise through distinct mechanisms: urinary gains are driven by adherence behaviours, while mental health benefits emerge through broader psychosocial pathways rather than symptom relief. These findings shift longstanding assumptions in PSO, highlighting that digital activation, behavioural engagement, and whole-person supports—not symptom changes—drive meaningful mental health recovery.

### 2.16. S4—106: Diet and Physical Activity as Mediators of Weight Loss in a Digital Prostate Cancer Survivorship Intervention

Nathan Smith, Gabriela Ilie, Rob Rutledge, Becky Power, Ricardo Rendon, Ross Mason, Andrea Kokorovic, Nikhilesh Patil, David Bowes, Greg Bailly, Derek Wilke, Elizabeth Langley and Cody MacDonald.
Dalhousie University, Halifax, Canada.

**Background/Rationale or Objectives/Purpose**

Weight gain during prostate cancer treatment worsens treatment-related side effects and increases cardiometabolic risk. PC-PEP, a 6-month digital survivorship program, improves lifestyle behaviours and weight outcomes. This analysis tested whether diet and physical activity mediated PC-PEP-related weight loss.

**Methodology or Methods**

In an RCT of 128 men (PC-PEP n = 66; standard care n = 62), diet was assessed using a 12-item dietary quality score (0–36), and physical activity was scored as low, moderate, or high. Weight was measured at baseline and 6 months. Mediation models adjusted for age, treatment modality, baseline BMI, mental health medication use, and comorbidity.

**Impact on Practice or Results**

PC-PEP participants showed significant weight reduction (mean −2.6 kg; *p* = 0.002). Diet improvement mediated 21% of weight loss (indirect β = 0.18, *p* = 0.01), while increased physical activity mediated 16% (indirect β = 0.14, *p* = 0.03), with a combined mediated proportion of 33%. A substantial direct effect remained (β = 0.42, *p* < 0.001), suggesting additional mechanisms such as mindfulness, self-regulation, and stress reduction.

**Discussion or Conclusions**

PC-PEP meaningfully improves diet and physical activity, and these changes mediate one-third of program-related weight loss. Importantly, the strong direct effect highlights that comprehensive digital interventions can shift multiple lifestyle, behavioural, and psychosocial determinants simultaneously, offering a scalable strategy to reduce cardiometabolic risk and strengthen survivorship outcomes in routine cancer care.

### 2.17. S4—109: PC-PEP Phase 4: Global Implementation of a Digital Prostate Cancer Survivorship Model for Psychosocial Oncology Care

Rob Rutledge ^1^, Gabriela Ilie ^1^, Ricardo Rendon ^1^, Ross Mason ^1^, Andrea Kokorovic ^1^, Nikhilesh Patil ^1^, Peter Dickens ^2^, Duvern Ramiah ^3^, Shinghai Mutambirwa ^4^, Rob Thompson ^5^, John Thoms ^6^, Howard Evans ^7^, Kunal Jana ^8^, Susan Ellard ^9^, Larry Pan ^10^, Chris Wallis ^11^ and Ernest Chan ^12^
^1^ Dalhousie University, Halifax, Canada;
^2^ Prostate Cancer Foundation of New Zealand, Auckland, New Zealand;
^3^ University of the Witwatersrand, Johannesburg, South Africa;
^4^ Dr George Mukhari Academic hospital and Sefako Makgatho Health Sciences University, Pretoria, South Africa;
^5^ Horizon Health, Saint John, Canada;
^6^ Memorial Hospital, St. John’s, Canada;
^7^ Alberta Health Services, Edmonton, Canada;
^8^ University of Saskatchewan, Saskatoon, Canada;
^9^ UBC Medical Oncology, Kelowna, Canada;
^10^ PEI Health, Charlottetown, Canada;
^11^ Mt Sinai Hospital, Toronto, Canada;
^12^ Oshawa Hospital, Oshawa, Canada.

**Background/** **Rationale or Objectives/Purpose**

Many prostate cancer survivors lack access to structured psychosocial and behavioural survivorship care. The PC-PEP program is a 6-month digital intervention integrating exercise, mindfulness, pelvic floor training, nutrition, intimacy support, and behavioural self-monitoring. Phase 4 evaluates global real-world implementation.

**Methodology or Methods**

Participants enrolled from Canada, New Zealand, and South Africa complete surveys at baseline, 6, 12, and 24 months assessing distress (K10), urinary/sexual function (EPIC), self-efficacy, adherence, satisfaction, and feasibility. Implementation outcomes include reach, uptake, retention, and cultural adaptation needs.

**Impact on Practice or Results**

Across 705 enrollees (Canada n = 650; NZ n = 50; SA n = 5), satisfaction was high (mean 8.3/10), and integration feasibility was rated 8.9/10. Early findings replicate RCT effects: distress decreased by −8.7 K10 points (*p* < 0.001), EPIC urinary incontinence improved by +13 points, and adherence mediated 41% of continence improvement. Program retention at 6 months exceeded 80%. Cultural adaptation needs were minimal, with digital literacy support emerging as the primary implementation requirement.

**Discussion or Conclusions**

PC-PEP demonstrates strong real-world feasibility, high acceptability, and clinically meaningful improvements across global settings. These findings show that digital survivorship programs can close longstanding equity gaps, extend PSO care beyond major centres, and offer scalable solutions for cancer systems worldwide—marking a significant shift toward accessible, behaviour-based survivorship care.

### 2.18. S5—Translating Couple Therapy Practices into Standard Cancer Care: Walking the Talk

**Moderator:** Andrew Mathew.
**Summary**

General evidence-based couple therapy practices often seem to flow in parallel to psychosocial interventions for couples in cancer care. The relative dearth of couple-based therapeutic treatment may be a function of (1) prioritizing individual over couple interventions because of limited psychosocial resources; (2) only offering couple therapy in the time-limited context of research trials (randomized controlled or feasibility); or (3) prioritizing coping with the demands of cancer over addressing the unique couple dynamic. Whether the reasons for limited integration of standard couple therapy into cancer care are philosophical, contextual, or resource related—the strong recommendation for more dyadically oriented supports for patients and their partners has persisted. The aim of this symposium is to demonstrate how empirically supported therapeutic techniques and approaches may become an integrated component of ‘treatment as usual’ for patients and partners.

Presentations in this symposium will adopt a transtheoretical approach to illustrating how couple therapy theory and technique may be productively integrated into sessions with patients and their partners even where the availability of psychosocial resources are limited. Systemically informed, therapeutic concepts will be illustrated with clinical case material and informed by qualitative interviews with couples coping with cancer of various types and stages of disease.

### 2.19. S5—131: Adapting a Single Session, Communal Coping Interview for Couples with Cancer

Karen Fergus ^1,2^, Nicole Carmona ^1,2^, Kimberley Thibodeau ^3^, Lucas Norton ^1^ and Sami Harb ^1^
^1^ York University, Toronto, Canada;
^2^ Sunnybrook Odette Cancer Centre, Toronto, Canada;
^3^ Cedars Cancer Centre, Montreal, Canada.

**Background/Rationale or Objectives/Purpose**

Evidence rooted in interdependence theory (how each partner’s coping reciprocally influences the other’s) suggests that partners who collaborate and work as a team in managing illness experience more positive outcomes, including reduced emotional distress and enhanced individual and dyadic efficacy. One model of interdependent coping, *communal coping,* emphasizes that having both (1) a shared appraisal of the disease (“ours” rather than “yours/mine”) and (2) collaborative action in managing the disease is predictive of positive health outcomes in couples facing chronic illness. The purpose of this talk is to discuss the potential for an empirically supported brief intervention for couples coping with chronic illness to improve psychosocial outcomes for couples affected by cancer.

**Methodology or Methods**

The Communal Coping Interview (“CCI”) is a single-session, narrative intervention that enhances collaborative coping and the couple’s experience of we-ness (or shared identity) in dyads where one member is living with type 2 diabetes. The CCI is designed to build upon couples’ existing strengths for coping with challenges and draw out their capacity for shared reflexivity. We are in the process of adapting the CCI as a method for supporting couples coping with cancer.

**Impact on Practice or Results**

Interventions that improve couples’ communal coping may enhance emotional and relational outcomes not only for the patient but also for their partner. The CCI has shown promise in supporting individual partner adjustment, while also strengthening the relationship. Thus, the potential for positive impact of the CCI in supporting couples affected by cancer is twofold.

**Discussion or Conclusions**

Although originally developed and tested with couples coping with chronic illness, the CCI may be adapted for couples affected by cancer. Moreover, because of its efficiency, the CCI has the potential to be readily integrated into routine cancer care increasing accessibility to dyadic treatment for individuals with cancer and their partners.

### 2.20. S5—132: Using the Genogram as an Assessment and Clinical Tool in Supporting Couples Through Cancer

Kimberley Thibodeau.
Cedars Cancer Centre, Montreal, Canada.

**Background/Rationale or Objectives/Purpose**

Couples facing a cancer diagnosis often experience profound disruptions to relational, emotional, and family system dynamics. While psychosocial oncology has increasingly emphasized relationally informed care, structured tools that integrate both assessment and intervention within couple-focused therapy remain underutilized. This presentation examines the genogram as a versatile, clinically robust tool for understanding multigenerational patterns, mapping sources of resilience and stress, and guiding therapeutic interventions for couples navigating the cancer trajectory.

**Methodology or Methods**

Drawing on clinical case material and evidence-informed frameworks, we demonstrate how genogram work helps couples externalize challenges, identify inherited coping styles, illuminate role expectations, and contextualize communication patterns that may influence their cancer experience. The genogram also facilitates exploration of cultural beliefs about illness, caregiving norms, and family narratives surrounding adversity, enabling clinicians to tailor psychosocial interventions with greater precision.

We discuss practical strategies for incorporating genograms across the cancer continuum—from diagnosis to survivorship or end-of-life—highlighting their utility in strengthening relational cohesion, enhancing meaning-making, and supporting shared decision making. Implications for clinical training, interdisciplinary collaboration, and integration within standard oncology care are outlined.

**Impact on Practice or Results**

By positioning the genogram as both an assessment method and an active psychotherapeutic tool, this work aims to advance systemic, relationship-centred approaches within psychosocial oncology practice.

**Discussion or Conclusions**

The genogram is a powerful technique with which to engage couple shared reflexive processing about their relationship and their interacting personal and familial histories. Insights gained via the genogram have the potential to enhance couple coping and adjustment to cancer.

### 2.21. S5—133: Couple Identity Across the Cancer Trajectory: Implications for Practice

Sami Harb ^1^, Amanda Piccirilli ^1^ and Karen Fergus ^1,2^
^1^ York University, Toronto, Canada;
^2^ Sunnybrook Odette Cancer Centre, Toronto, Canada.

**Background/Rationale or Objectives/Purpose**

For couples facing cancer, research suggests that bolstered couple identity or “we-ness” promotes the couple’s dyadic coping and resilience. However, there is a lack of research on how we-ness is expressed across the cancer continuum stages (e.g., diagnosis, treatment, survivorship). This exploratory, comparative mixed-methods study examines themes representative of coping and adjustment processes for couples reporting varying degrees of we-ness.

**Methodology or Methods**

Semi-structured interviews were conducted with individuals who met the following criteria: were diagnosed with breast, prostate, or colon cancer; were in a committed relationship at the time of diagnosis; and who have completed their treatment. Prior to being interviewed, participants responded to a 7-point survey item about how they think their relationship has changed since going through cancer (1 = became significantly worse, 4 = no change, 7 = greatly improved). During the interviews, they were asked to discuss ‘critical incidents’ that were significant to their relationship bond from the time of diagnosis through to recovery. A sub-analysis of the interview content of individuals reporting mild-to-no-change (scores of 3–5) in their relationship bond vis à vis cancer is underway. This analysis interweaves the qualitative reports with scores on a measure of we-ness to understand the processes through which couples maintain their level (e.g., low, moderate, or high) of couple identity. Qualitative analysis will generate a framework intended to deepen understanding of we-ness across the cancer trajectory.

**Impact on Practice or Results**

Our results will facilitate understanding of the relationship vulnerabilities or markers for risk that may be particular to different stages of the cancer continuum, relationship processes that are protective of we-ness, and how targeted therapeutic interventions might aid couples who are struggling at different stages of treatment.

**Discussion or Conclusions**

This research hopes to shed light on what relationship processes may contribute to feelings of closeness or distance for couples navigating the demands of cancer treatment and recovery.

### 2.22. Final Category: A. Adapting PSO Care in LMI Countries

56: Building Psychosocial Knowledge and Skills Among Nurses Caring for Cancer Patients in Rwanda
Margaret Fitch.
East York, East York, Canada.

**Background/Rationale or Objectives/Purpose**

Individuals with cancer living in Africa cope with physical, emotional, and practical challenges for which psychosocial interventions could be helpful. Nurses are in an ideal position to identify individuals who are experiencing difficulties and need added support. However, nurses in Rwanda who care for cancer patients have indicated they need to learn more about psychosocial care.

**Methodology or Methods**

A tailored education program was designed to build capacity of nurses caring for cancer patients in providing psychosocial care in Rwanda. During a two-day professional development workshop held for nursing staff lectures, interactive sessions, and case study analysis regarding foundational psychosocial care knowledge and skills was facilitated by an interdisciplinary team. A pre–post questionnaire was utilized to evaluate learning; a workshop satisfaction survey was used to evaluate attendee responses and recommendations.

**Impact on Practice or Results**

Three workshops were held in a setting outside the treatment facility. Thirty-seven nurses participated from pediatric and adult inpatient and ambulatory settings. Thirty-four returned pre- and post-questionnaires. All but two indicated experiencing a change in their confidence and comfort levels with psychosocial knowledge and skills following the workshop. Change scores ranged from 1 to 25 out of a possible 32 (mean = change 9.1).

**Discussion or Conclusions**

Beginning with a workshop approach to share knowledge, contextualized for the setting, offers a foundation for improvement in psychosocial care. Follow-up is needed to continue to build the psychosocial competencies of nurses caring for cancer patients in the clinical setting.

Questions: What would you see as barriers to implementing this approach in other settings? What would you recommend as relevant and appropriate follow-up after an introductory session such as is described here?

### 2.23. Final Category: B. Cancer Care Across the Life Span (Children, Adolescent and Young Adults, Adults, and Older Adults)

#### 2.23.1. 12: Medical Assistance in Dying Use Among Adolescent and Young Adult Cancer Patients: A Mixed-Methods Study

Emilie Muth ^1^, Nicole Maseja ^2^, Andrew Harper ^2^, Siwei Qi ^3^, Linda Watson ^3^, Claire Link ^3^, Janet Vandale ^4^, Kathleen Reynolds ^5^, Ursula Sansom-Daly ^6^, James Silvius ^7^ and Miranda Fidler-Benaoudia ^2,8^
^1^ Department of Oncology, University of Calgary, Calgary, Canada;
^2^ Department of Cancer Epidemiology and Prevention Research, Cancer Care Alberta, Calgary, Canada;
^3^ Department of Supportive Care Services and Patient Experience, Cancer Care Alberta, Calgary, Canada;
^4^ Assisted Living Alberta, Calgary, Canada;
^5^ Department of Family Medicine, University of Calgary, Calgary, Canada;
^6^ Behavioural Sciences Unit, UNSW Sydney, Sydney, Australia;
^7^ Department of Medicine, University of Calgary, Calgary, Canada;
^8^ University of Calgary, Calgary, Canada.

**Background/Rationale or Objectives/Purpose**

To understand medical assistance in dying (MAiD) utilization in the adolescent and young adult (AYA) cancer population.

**Methodology or Methods**

This mixed-methods, retrospective cohort study included all patients in Alberta, Canada who were diagnosed with a first primary cancer between the ages of 15 and 39 years and who received a MAiD provision for cancer from 2017 to 2022. Descriptive statistics were calculated to summarize patient, cancer, and MAiD characteristics. Symptom burden in the year before death was modelled. Qualitative analyses were summarized using thematic analysis and then integrated with quantitative findings using joint display.

**Impact on Practice or Results**

A total of 34 AYA cancer patients accessed MAiD, with provisions peaking in 2020. Individuals were slightly more likely to be female (52.9%), 91.2% lived in an urban zone, 29.4% had children, and over half received at least three types of cancer therapy. While symptom burden was low at month 5–11 prior to death, a crisis period followed at months 3–5 before death, with statistically significant (*p* < 0.05) increases observed in all domains except pain and lack of appetite. Despite this, half of participants received a late palliative care referral (<3 months before death), with 71.4% reporting high symptom complexity in the last month of life and 80% reporting a loss of ability to engage in activities making life meaningful at time of MAiD provision. Qualitative themes offered context into the patient experience and include social isolation, previous experience with cancer death, wanting control, and acceptance of their situation.

**Discussion or Conclusions**

Healthcare providers should use patient-reported symptom burden to trigger palliative care referrals, where a more reactive response to initial symptom increases could avoid AYA cancer patients reaching a crisis state, ultimately reducing suffering and improving experiences at end-of-life.

#### 2.23.2. 16: A Thematic Analysis of Perspectives of Canadian Healthcare Practitioners Working with Adolescent and Young Adult Patients (AYA) in Designing a Regional AYA Program

Sreejita Das ^1^, Mehreen Rashid ^1^, Sarah Cleyn ^2^ and Amirrtha Srikanthan ^1,2^
^1^ University of Ottawa, Ottawa, Canada;
^2^ The Ottawa Hospital Cancer Centre, Ottawa, Canada.

**Background/Rationale or Objectives/Purpose**

When adolescents and young adults (aged 15–39) (AYAs) are diagnosed with cancer, their quality-of-life changes drastically. Despite the unique needs of AYA cancer patients, there are gaps in age-specific care and research. This study explored the experiences and perspectives of HCP when working with AYA cancer patients to develop a tailored program to better support AYA needs.

**Methodology or Methods**

Qualitative semi-structured interviews were conducted with HCPs who work with AYA cancer patients in the Champlain region of Ontario, Canada between November 2024 and February 2025. An experienced interviewer conducted the interviews, which were recorded, transcribed verbatim and analysed thematically. Thematic analysis focused on key domains of discussions for AYA care: mental health, sexual health/fertility, financial impacts, and palliative care.

**Impact on Practice or Results**

Fourteen participants were interviewed; twelve identified as women. Their professions comprised five physicians, five nurses, two technicians, one social worker and one pharmacist. The key themes identified included the following:

(1) **HCP unpreparedness for difficult discussions**: (i) Need more training/resources for discussions; (ii) indirectly addressing “taboo” topics.
(2) **Improving communication**: (i) Communication between HCP teams; (ii) more empathy in conversations with patients; (iii) need for accessible communication strategies; (iv) power of open-ended questions.
(3) **Complex navigation needs for AYA**: (i) Lack of patient advocacy and navigation; (ii) need for HCP role clarity; (iii) unique support needs for AYA.

**Discussion or Conclusions**

This cohort of HCPs highlighted challenges in providing care to AYA populations and the findings will ultimately inform future programming to better meet their needs.

#### 2.23.3. 42: The Alarming Prevalence of Traumatic Stress in Caregivers of Pediatric and Adult Family Members with Cancer: Baseline Results from a Multimethod Longitudinal Study

Stephanie Nanos ^1,2,3^, Lindsay Jibb ^1,3^, Sarah Alexander ^4,5^, Charlotte Hilberdink ^6^, Katharina Schultebraucks ^6,7^, Anne Rydall ^2^, Camilla Zimmermann ^2,3^, Rinat Nissim ^2,3^, Sarah Hales ^2,3^ and Gary Rodin ^2,3^
^1^ Hospital for Sick Children, Toronto, Canada;
^2^ Princess Margaret Cancer Centre, Toronto, Canada;
^3^ University of Toronto, Toronto, Canada;
^4^ BC Children’s Hospital, Vancouver, Canada;
^5^ University of British Columbia, Vancouver, Canada;
^6^ NYU Langone Health, New York, USA;
^7^ NYU, New York, USA.

**Background/Rationale or Objectives/Purpose**

The diagnosis and treatment of cancer can be profoundly traumatic for family caregivers (FCs), who are repeatedly exposed to the threat or reality of the patient’s death. As a result, FCs may experience clinically significant traumatic stress symptoms (TSSs), placing them at risk for serious health outcomes. Yet, systematic assessments are limited, and evidence on effective psychosocial support for TSS remains unclear. This study aimed to characterize the prevalence and severity of TSS among FCs after diagnosis, and to examine its longitudinal course, predictors, and related care needs over the first year to identify targets for early intervention and prevention.

**Methodology or Methods**

FCs of pediatric and adult patients with cancer were recruited from two metropolitan cancer centers within 3 months of diagnosis. Participants completed self-report measures of TSS and related psychosocial outcomes at enrolment and at 3, 6, 9, and 12 months after diagnosis. Baseline quantitative data were analyzed descriptively and using machine learning approaches. A subset of FCs participated in semi-structured interviews at 3, 6, and/or 12 months, and these were analyzed thematically.

**Impact on Practice or Results**

A total of 128 FCs were enrolled (i.e., 99 parents, 24 partners, 4 adult children, 1 sibling) and 30 participated in interviews. At baseline, 76 FCs (59.4%) experienced moderate-to-severe TSS and 46 (35.9%) experienced probable PTSD. Attachment anxiety, higher educational attainment, and caring for a patient ≤ 18 years emerged as key predictors. Qualitative findings aligned with these patterns: FCs described uncertainty, hypervigilance, and exhaustion that permeated daily life, and consistently prioritizing the patient’s needs while neglecting their own but expressing openness to proactive professional support.

**Discussion or Conclusions**

TSSs are common among FCs across the life course after a cancer diagnosis. Systematic assessment and early intervention are needed to prevent and alleviate TSSs and to improve family-centered care.

#### 2.23.4. 67: Helps Them Reclaim Their Sense of Self’: Teen Cancer Connection Program Fills a Gap in Pediatric Psychosocial Oncology Care

Michelle Rianto ^1^, Karren Xiao ^1^, Rebecca Côté ^2^ and Chana Korenblum ^2^
^1^ Temerty Faculty of Medicine, University of Toronto, Toronto, Canada;
^2^ The Hospital for Sick Children, Toronto, Canada.

**Background/Rationale or Objectives/Purpose**

Adolescents and young adults (AYAs) with cancer have biopsychosocial needs that are often unmet in healthcare systems designed for pediatric or older adult populations. To address these gaps, developmentally tailored AYA programs have emerged, primarily within adult hospitals. In 2023, following a needs assessment that identified a lack of age-appropriate psychosocial care, the first AYA program in a Canadian pediatric center—Teen Cancer Connection (TCC)—was launched. This study evaluates the program’s effectiveness after two years by surveying AYA oncology patients and healthcare staff.

**Methodology or Methods**

Nine patients (ages 14–17) have been surveyed, with recruitment ongoing. Most patients identified as male (5/9), and leukemia was the most common diagnosis (6/9). Patient satisfaction was high: 67% of respondents agreed or strongly agreed that TCC met their needs; 89% said that they would recommend TCC to peers.

Forty staff survey responses were received from diverse disciplines, primarily medicine, nursing, social work, and child life. Feedback emphasized strong rapport with teens, developmentally appropriate support, and positive impact on emotional wellbeing. Staff consistently described the TCC nurse as compassionate, energetic, and effective. Suggested improvements included increasing program visibility across inpatient units.

**Impact on Practice or Results**

Findings reinforce the critical role of developmentally tailored psychosocial care for AYA patients in pediatric settings. High satisfaction and positive feedback validate the program’s role in meeting unique needs. Insights will guide program improvements to serve as a model for AYA care.

**Discussion or Conclusions**

Positive feedback about TCC supports integration across pediatric centers to address persistent gaps. Future research should explore scalability and long-term outcomes to ensure sustained benefits.

**Questions for feedback:**

What strategies do you recommend for effectively soliciting patient feedback and improving recruitment for the TCC program, given limited funds and resources?

How would you involve patients and families in shaping the program design and ongoing improvements?

#### 2.23.5. 74: Factors Associated with Perceived Social Support in Adolescents and Young Adults with Cancer

Reanna George ^1,2^, Pam Crotty ^3^, Geoff Eaton ^4^ and Sheila Garland ^1,2^
^1^ Memorial University of Newfoundland, St. John’s, Canada;
^2^ Beatrice Hunter Cancer Research Institute, Halifax, Canada;
^3^ Young Adult Cancer Canada, Calgary, Canada;
^4^ Young Adult Cancer Canada, St. John’s, Canada.

**Background/Rationale or Objectives/Purpose**

Adolescents and young adults (AYAs) with cancer face unique developmental challenges that can be impacted by their diagnosis. For some, social support may play an important role in decreasing the psychological impacts of cancer in AYAs and increase their ability to cope. The current study used data from the Young Adult Cancer Canada (YACC) Recover study to examine factors associated with perceived social support.

**Methodology or Methods**

Canadian AYAs diagnosed with cancer aged 18–39 completed an online survey. The Social Provisions Scale was used to measure perceived social support. Univariable and multivariable linear regressions were used to examine demographic, cancer-related, and psychological variables associated with social support.

**Impact on Practice or Results**

Participants (N = 564, M_age_ = 32.45, 72.2% women) completed the survey. At the multivariable level, being male (*b* = −0.368, *p* ≤ 0.001) and having higher levels of depression (moderate *b* = −0.316, *p* = 0.049; moderately severe *b* = −0.589, *p* = 0.004; severe *b* = −0.820, *p* = 0.020) were significantly associated with lower social support. Being in a relationship (*b* = 0.667, *p* ≤ 0.001) and having higher resilience (*b* = 0.255, *p* ≤ 0.001) were significantly associated with greater social supports. Age, being in treatment, time since diagnosis, having children, living situation, being connected to the AYA cancer community, quality of life, and anxiety were not significantly associated with social support.

**Discussion or Conclusions**

Adequate social support may play an important role in cancer recovery for AYAs. Targeted supports for men and those with higher levels of depression might be needed as they are at higher likelihood of experiencing low social support.

#### 2.23.6. 84: Preliminary Feasibility Data in a Multisite, Multicomponent Intervention for Families in Pediatric Oncology

Nikita Guarascio ^1,2^, Daniel Curnier ^1,2^, Caroline Laverdière ^1,2^, Raoul Santiago ^3,4^, Stéphanie Vairy ^5,6^, Émélie Rondeau ^2^ and Serge Sultan ^1,2^
^1^ Université de Montréal, Montreal, Canada;
^2^ CHU Sainte-Justine, Montreal, Canada;
^3^ CHU de Québec, Québec City, Canada;
^4^ Université Laval, Québec City, Canada;
^5^ CHU de Sherbrooke, Sherbrooke, Canada;
^6^ Université de Sherbrooke, Sherbrooke, Canada.

**Background/Rationale or Objectives/Purpose**

More than 50% of pediatric cancer survivors later develop serious chronic conditions. To help prevent long-term effects and reduce parental distress, researchers developed an evidence-based program called VIE (*Valorization*, *Implication*, *Education*), comprising three components: nutritional support, physical activity and psychosocial support. This work aims to describe preliminary feasibility data and participant flow in each component, with particular emphasis on the psychosocial intervention.

**Methodology or Methods**

We are recruiting patients receiving treatment and diagnosed with cancer before the age of 21, along with their parents, from three pediatric oncology centers in the Province of Québec. We estimate that approximately 75% of patients will be eligible. We anticipate enrolling 125 families. Children and adolescents participate in nutrition and physical activity counselling sessions over 52 weeks. Parents participate in up to six sessions of *Taking Back Control Together* (TBCT), a manualized intervention designed to improve problem-solving skills and dyadic coping. Parents complete questionnaires assessing problem-solving, perceived competence, perceived stress, anxiety, depression, dyadic coping, and social validity at three time points. Clinicians complete checklists after each session to document treatment fidelity.

**Impact on Practice or Results**

To date, 98 families have been eligible to participate in VIE, 31 children have been enrolled in the nutrition and physical activity components, and 45 parents have been enrolled in TBCT (51% mothers). Exposure to TBCT components is not uniform across families and will be presented in detail.

**Discussion or Conclusions**

This work will highlight recruitment/enrollment patterns during treatment and preliminary feasibility results within a multisite, multidisciplinary program, with particular focus on the psychosocial intervention designed to support parents.

#### 2.23.7. 85: The Fear of Cancer Recurrence Therapy for Adolescents and Early Young Adults Survivors of Childhood Cancer (FORT-AeYA): Adaptation Results

Paige Tough ^1^, K. Brooke Russell ^1^, Celeste Holy ^1^, Tegan Lyster ^2^, Alia Johnston ^3^ and Sophie Lebel ^1^
^1^ University of Ottawa, Ottawa, Canada;
^2^ Patient Partner, Saskatoon, Canada;
^3^ Patient Partner, Victoria, Canada.

**Background/Rationale or Objectives/Purpose**

Fear of cancer recurrence (FCR) is the top unmet need among survivors of cancer. Thirty to sixty percent of adolescent and early young adult (AeYA) survivors of childhood cancer (ages 13–25) experience FCR. No FCR interventions exist for AeYAs. The Fear of Cancer Recurrence Therapy (FORT) is an efficacious group intervention that reduces FCR for survivors of adult cancer. Our objective was therefore to adapt FORT for the unique needs of AeYAs (FORT-AeYA).

**Methodology or Methods**

An advisory board of seven patient partners and four researchers met bi-weekly via Zoom over four months to iteratively adapt the six-session FORT manual. Using the Framework for Reporting Adaptations and Modifications to Evidence-based Interventions—Enhanced (FRAME), two independent coders will code each modification to the FORT manual. Modifications will be coded based on eight questions, for example: what was modified, what is the nature of the content modification, and what is the reason for the modification. Descriptive statistics will be used to summarize the results.

**Impact on Practice or Results**

Preliminary results based on one coder indicate that 85 modifications were made to the manual. The majority were content modifications to tweak the protocol and improve fit for AeYAs. Secondary coding is currently underway, with full results expected by the time of the presentation.

**Discussion or Conclusions**

Results of the FRAME coding will highlight the types of changes required to adapt FORT for AeYAs. The adapted manual will be used for usability and pilot testing, with the ultimate goal of improving care for survivors of childhood cancer.

1. Does the FRAME framework adequately capture the developmental nuances of adapting the FORT manual for a younger audience?
2. Does anyone have general feedback on strategies for recruiting survivors of childhood cancer into usability and pilot studies?

#### 2.23.8. 88: Bridging the Gap—A Protocol for Understanding the Transition from Pediatric to Adult Oncology Care

Maya Stern ^1^, Jaden Paltoo ^1^, Matthew Langiano ^1^ and Chana Korenblum ^1,2^
^1^ UHN, Toronto, Canada;
^2^ SickKids, Toronto, Canada.

**Background/Rationale or Objectives/Purpose**

Adolescents and young adults (AYAs) diagnosed with cancer aged between 13 and 18 years or on active treatment face a critical transition from pediatric to adult oncology care. This shift can disrupt continuity of care and heighten distress, affecting medical and psychosocial outcomes. Despite its importance, standardized transition pathways are lacking. This multi-phase project aims to understand and improve this process in a large Ontario cancer centre. Phase One focuses on exploring facilitators and barriers to successful transition.

**Methodology or Methods**

In Phase One, a literature review of global transition practices in pediatric and adult oncology is being conducted (n ≥ 25 studies), alongside an environmental scan of transition processes across Canadian oncology centres (n ≥ 5 pediatric and ≥5 adult centres). Finally, the experiences of patients, caregivers, and healthcare providers will be explored through semi-structured interviews (n ≥ 10 per group). Patient partners are being recruited to ensure a patient-centred approach and to provide contextual insight for interpreting findings.

**Impact on Practice or Results**

Early findings show that existing frameworks do not fully support youth as they transition through cancer care. Gaps in care contribute to delays in surveillance, missed opportunities for early intervention, and increased risk of adverse physical and mental health outcomes.

**Discussion or Conclusions**

This project will generate the critical evidence base required to co-design a robust framework that addresses the gap in psychosocial care for adolescents with cancer transitioning to adult care. The resulting framework has potential for scalability across other cancer centres and adaptation for transitions in other chronic illnesses, supporting broader improvements in continuity and patient-centred care.

#### 2.23.9. 89: Supportive Care Needs Among Individuals with Endometrial Cancer: A Scoping Review

Kaviya Devaraja ^1^, Megha Manoj ^1^, Sharlane C. L. Lau ^1^, Sarah Daniels ^1^, Bianca Lima ^1^, Rouhi Fazelzad ^2^, Rachel Soyoun Kim ^3,4^, Sarah Ferguson ^3,4^, Shima Deljoomanesh ^3^, Stefane Laframboise ^3,4^, Madeline Li ^1,5^ and Aliza Panjwani ^1,5^
^1^ Department of Supportive Care, Princess Margaret Cancer Centre, University Health Network, Toronto, Canada;
^2^ Library and Information Services, Princess Margaret Cancer Centre, University Health Network, Toronto, Canada;
^3^ Division of Gynecologic Oncology, Princess Margaret Cancer Centre, University Health Network, Toronto, Canada;
^4^ Department of Obstetrics and Gynecology, University of Toronto, Toronto, Canada;
^5^ Department of Psychiatry, Temerty Faculty of Medicine, University of Toronto, Toronto, Canada.

**Background/Rationale or Objectives/Purpose**

Endometrial cancer (EC) is the most common gynecological malignancy in North America, and its rising incidence and improving survival rates across age groups underscore the need to better understand the supportive care needs (SCNs) of those who are affected. Consequently, this scoping review aims to identify the SCNs of patients with EC and, where available, explore the distinct needs of younger adults within this population.

**Methodology or Methods**

The review follows the Joanna Briggs Institute guidelines and Arksey and O’Malley’s framework for scoping reviews. We searched seven databases for peer-reviewed English-language studies on SCNs among adults with EC, without restrictions on country, publication date, disease stage, or treatment type. Intervention studies, grey literature, and studies involving mixed cancer samples without EC-specific analyses were excluded. Each abstract and full text was screened by two reviewers from Covidence. Eligible studies are undergoing data extraction.

**Impact on Practice or Results**

Of the 13,446 screened abstracts, 713 studies underwent full-text review, and 130 met the inclusion criteria. Preliminary findings from 55 studies show a strong concentration of research in the physical (e.g., fatigue, pain) and psychological (e.g., anxiety, depression) SCN domains. Comparatively less attention was directed toward informational, interpersonal, daily living, and health system communication needs. Evidence addressing younger adults’ SCNs was minimal.

**Discussion or Conclusions**

The mapped literature suggests that the existing research does not fully capture the supportive care challenges faced by people with EC, particularly those shaped by developmental, sociocultural, or system-level factors. Advancing comprehensive supportive care will require addressing these evidence gaps through studies that broaden the conceptualization and measurement of SCNs in EC.

#### 2.23.10. 92: Exploring Body Image Complexity in Adolescent and Young Adult Cancer Survivors: A Hermeneutic Study

Alana Ireland ^1^, Nancy Moules ^2^ and Shelly Russell-Mayhew ^2^
^1^ Athabasca University, Athabasca, Canada;
^2^ University of Calgary, Calgary, Canada.

**Background/Rationale or Objectives/Purpose**

Often faced with the challenge of finding themselves in a body that is different from that which they knew before cancer, adolescents and young adults (AYAs) are a distinct group within oncology already going through significant developmental changes. The goal of this research was to examine the complexity of body image in adolescent and young adult (AYA) cancer survivors to inform a more responsive approach to supporting these young people following their cancer treatment.

**Methodology or Methods**

Recruited from across Canada, participants included AYA cancer survivors (n = 13) and oncology healthcare professionals working with AYAs (n = 6). Unstructured interviews were conducted with healthcare professionals and AYAs to explore their perceptions of body image in the context of AYA oncology (healthcare professionals) and their personal body image experiences (AYAs). Interviews were 60 to 90 min and interpreted using Gadamer’s philosophical hermeneutics.

**Impact on Practice or Results**

Findings provided an in-depth understanding of the complexity of body image in the context of AYA cancer survivorship. Hermeneutic interpretations explored (a) development through the lens of cancer, (b) body appreciation and acceptance, (c) grief for the body lost, (d) identity, and (e) scars.

**Discussion or Conclusions**

A more comprehensive understanding of how AYAs experience, make meaning of, and understand body image post cancer, translates into best-practice knowledge to inform treatment and provision of care from interdisciplinary teams.

#### 2.23.11. 96: Exploring Factors Associated with Sexual Functioning Related Distress in Adolescents and Young Adults with Cancer

Anna Feehan ^1^, Reanna George ^1,2^, Pam Crotty ^3^, Geoff Eaton ^4^ and Sheila Garland ^1,2,5^
^1^ Department of Psychology, Faculty of Science, Memorial University of Newfoundland, St. John’s, Canada;
^2^ Beatrice Hunter Cancer Research Institute, Halifax, Canada;
^3^ Young Adult Cancer Canada, Calgary, Canada;
^4^ Young Adult Cancer Canada, St. John’s, Canada;
^5^ Discipline of Oncology, Faculty of Medicine, Memorial University of Newfoundland, St. John’s, Canada.

**Background/Rationale or Objectives/Purpose**

A cancer diagnosis can profoundly impact adolescents and young adults (AYAs; 15–39 years). Sexual dysfunction is reported in about 50% of AYAs. This study investigates factors associated with sexual functioning related distress in AYAs with cancer using baseline data from the Young Adult Cancer Canadas (YACC) RECOVER study.

**Methodology or Methods**

Canadian AYAs with cancer, aged 18–39 (N = 538, 76% women), completed an online survey. Univariable and multivariable logistic regressions were used to explore demographic, cancer-related, and psychological variables associated with the question “In the PAST WEEK, how much DISTRESS have you experienced as a result of the following: Sexual Issues (e.g., Pain, sex Drive?)” from the CDS-AYA scale. Responses of “none” were coded as “no distress”; all others were coded “distressed”.

**Impact on Practice or Results**

Participants with gynecological, breast and gastrointestinal cancers reported the greatest distress. At the multivariable level, compared to those who are single, AYAs in a relationship endorsed more distress regarding sexual functioning (*AOR* = 2.895, *p* < 0.001). Compared to those who reported no depressive symptoms, having mild depressive symptoms was associated with greater sexual distress (*AOR* = 2.183, *p* = 0.003). Compared to those completed treatment, not having received (*AOR* = 0.112, *p* < 0.001) or not having completed treatment (*AOR* = 0.387, *p* < 0.001), were associated with less sexual functioning distress. Perceived social support (*AOR* = 0.860, *p* < 0.001) was associated with less sexual functioning distress.

**Discussion or Conclusions**

Understanding which factors are associated with sexual functioning related distress in AYAs can help inform proper interventions and supports.

#### 2.23.12. 112: Factors Associated with Cancer-Related Fatigue in Young Adults with Cancer

Steven Power ^1^, Reanna George ^1,2^, Pam Crotty ^3^, Geoff Eaton ^4^ and Sheila Garland ^1,2^
^1^ Memorial University of Newfoundland, St. John’s, Canada;
^2^ Beatrice Hunter Cancer Research Institute, Halifax, Canada;
^3^ Young Adult Cancer Canada, Calgary, Canada;
^4^ Young Adult Cancer Canada, St. John’s, Canada.

**Background/Rationale or Objectives/Purpose**

Adolescents and young adults (AYAs; 15–39 yrs) with cancer report more fatigue than healthy AYAs (48% to 20%, respectively). Identifying factors associated with fatigue allows for better recognition of risk factors and the development of tailored interventions. The current study used data from the Young Adult Cancer Canada (YACC) Recover study to examine factors associated with fatigue.

**Methodology or Methods**

Canadian AYAs diagnosed with cancer aged 18–39 completed an online survey. The fatigue subscale from the EORTC QLQ-C30 was used to measure fatigue. Univariable and multivariable linear regressions were used to examine demographic, cancer-related, and psychological variables associated with fatigue.

**Impact on Practice or Results**

Participants (N = 564, M_age_ = 32.5, 72% women) completed the survey. At the multivariable level, less life satisfaction (b = −0.090, *p* = 0.017), anxiety (mild; b = 0.236, *p* = 0.003; moderate: b = 0.444, *p* ≤ 0.001; moderately severe: b = 0.577, *p* ≤ 0.001), higher fears of recurrence (b = 0.089, *p* = 0.011), and greater physical distress (b = 0.422, *p* ≤ 0.001) were associated with greater fatigue. Completion of treatment (b = −0.227, *p* = 0.006) was associated with less fatigue. Relationship status, not receiving treatment, and time since diagnosis were not significantly associated with fatigue.

**Discussion or Conclusions**

The ability to recognize potential risk factors for fatigue can lead to better quality of life for AYAs cancer survivors. The current findings demonstrate actionable targets that can be modified to improve fatigue in AYAs with cancer.

#### 2.23.13. 113: Simple Treasures: The Impact of Sensory Tools in AYA Cancer Care

Simone Kurup.
Princess Margaret Cancer Centre, Toronto, Canada.

**Background/Rationale or Objectives/Purpose**

Adolescents and young adults (AYAs) with cancer have distinct psychosocial needs that require specialized care. Sensory/fidget tools, which support emotional regulation, have demonstrated benefits for AYAs. Since the emotional impact of a cancer diagnosis can disrupt coping strategies, accessible interventions that reduce stress are essential. The AYA Program at the Princess Margaret Cancer Centre (PMCC) explored the use of sensory tools to enhance rapport, support grounding, and reduce maladaptive coping among AYAs.

**Methodology or Methods**

Despite increasing AYA psychosocial oncology resources, AYAs consistently report higher levels of distress than older adults. In response, the AYA Program introduced a portable collection of evidence-informed sensory and fidget items housed in a treasure chest. Items are low-cost, and easily accessible for staff to offer, particularly when AYAs express anxiety, emotional dysregulation, or difficulty coping.

**Impact on Practice or Results**

Since the initiative began in February 2025, approximately 200 sensory items have been distributed across the program. Feedback from AYAs and families indicates that these tools provide comfort, support emotional regulation, and meet AYA needs. Clinicians report that the items promote rapport building, normalize mental health discussions, and creating opportunities for psychosocial engagement.

**Discussion or Conclusions**

Providing a tangible object that is familiar and therapeutically supportive has proven impactful in AYA cancer care. The initiative’s adaptability supports broader implementation across settings and continued evaluation will guide refinement and sustainability.

*How feasible do you think integrating sensory/fidget tools would be in your clinical or community setting?*

*How could this approach be improved or adapted to maximize impact for AYAs?*

#### 2.23.14. 116: Spreading the Word: How Engagement with Media Is Relevant to Adolescent and Young Adults (AYAs) Care and Why Clinicians Need to Be a Part of It

Jennifer Catsburg ^1,2^, Simone Kurup ^1^, SarahRose Black ^1,2^, Abha Gupta ^1,2,3^ and Samantha Scime. ^1,2^
^1^ University Health Network, Toronto, Canada;
^2^ University of Toronto, Toronto, Canada;
^3^ Sickkids, Toronto, Canada.

**Background/Rationale or Objectives/Purpose**

Increasingly, media outlets are seeking stories about the AYA cancer experience. Patient and clinician interaction with media offers opportunities for increased program funding, improved access to care, as well as opportunity for AYAs to explore their sense of self and wellbeing situated within the larger cancer system. Participating in this experience as AYA clinicians is imperative; ensuring patient safety, correct messaging, and space for debrief and reflection.

**Methodology or Methods**

Recently, AYAs and their care providers at the Princess Margaret Cancer Centre have engaged in media interviews across various modalities. These media experiences have prompted clinicians to pursue formal media training to better advocate for specialized programming and to ensure adequate psychosocial support for their patient participants.

**Impact on Practice or Results**

Encouraging AYA patients and their care providers to participate in media interviews and providing structured support before, during, and after these engagements offers a unique and essential opportunity to expand public awareness and improve access to tailored AYA care across Canada. Notably, supporting AYAs through media engagement presents new challenges for clinicians, including navigating the emotional impact on patients and reflecting on their own sense of contribution and professional productivity.

**Discussion or Conclusions**

Insights from the Princess Margaret Cancer Centre AYA Program underscore the value of being accessible and prepared to guide AYAs through these interactions. Perspectives from both clinicians and patients experience with media will be shared.

#### 2.23.15. 126: National Environmental Scan to Inform Development of Supportive Care Resources for Adolescents and Young Adults with High-Grade Glioma in Canada

Kaviya Devaraja ^1,2^
^1^ Institute of Medical Science, University of Toronto, Toronto, Canada;
^2^ Adolescent and Young Adult Program, Department of Supportive Care, Princess Margaret Cancer Centre, University of Toronto, Toronto, Canada.

**Background/Rationale or Objectives/Purpose**

Adolescents and young adults (AYAs) with high-grade glioma (HGG) face complex medical and psychosocial needs that are often unmet due to fragmented and inconsistent support services across Canada. To address these gaps, this study conducted a national environmental scan to better understand the availability, consistency, and comprehensiveness of AYA-specific supportive care resources. The goal was to document existing services, identify inequities, and lay the foundation for developing a national AYA HGG Resource Navigator.

**Methodology or Methods**

An environmental scan was completed across 22 major cancer centres in Canada and five national AYA or brain-tumour-focused resource and advocacy organizations. Publicly available information was reviewed, and interviews were conducted with healthcare providers to document current services in domains such as mental health, peer support, fertility, cognitive rehabilitation, education and career transition, and caregiver support. Data were analyzed to assess national variability, identify service gaps, and highlight areas requiring targeted resource development.

**Impact on Practice or Results**

Findings revealed substantial inconsistencies in AYA-focused supports across provinces and institutions, with major gaps in system navigation, cognitive rehabilitation, and transition resources. Results will directly inform the creation of a centralized national AYA HGG Resource Navigator, an educational whiteboard video, and new patient- and caregiver-focused supports developed in partnership with the Brain Tumour Foundation of Canada.

**Discussion or Conclusions**

This environmental scan highlights critical inequities and opportunities to standardize supportive care for AYA HGG patients. Future directions include developing the identified resources and conducting a mixed-methods evaluation with AYAs, caregivers, and healthcare providers to assess their usability, effectiveness, and impact on patient-centered care nationally.

Questions:

□ What additional AYA-specific supportive care domains should be considered in the development of the national AYA HGG Resource Navigator?
□ How can the resource navigator and educational video be optimized to meet the needs of diverse AYA populations across provinces and care settings?
□ What strategies would best support national implementation and integration of these tools into routine clinical pathways?

#### 2.23.16. 128: The Whole Family: Supporting Our Young Cancer Patients from a Family-Centred Perspective

Ashley Mikitzel and Bridget Veltri.
Juravinski Cancer Centre, Hamilton, Canada.

**Background/Rationale or Objectives/Purpose**

The Juravinski Cancer Centre in Hamilton, Ontario launched a dedicated program for Adolescent and Young Adult (AYA) for patients aged 18–39 in October 2025. This program is dedicated to addressing the unique factors and challenges young patients face when diagnosed with cancer. A unique aspect of the Juravinski AYA Program is the involvement of a Child Life Specialist (CLS) who supports the children of our cancer patients. The CLS supports children with age-appropriate information on cancer through therapeutic play, emotional expression and teaching of coping strategies.

**Methodology or Methods**

We will provide an overview on how the AYA and CLS program is embedded within PSO yet provides unique support outside the Social Work and psychology teams. We will discuss the implementation process through multidisciplinary consultation, practical integration of these programs including referrals, follow-ups, and triaging as well as published research on the CLS role.

**Impact on Practice or Results**

We will discuss the impact the AYA and CLS programs have on the PSO department at the Juravinski Cancer Centre. We will also provide anecdotal feedback from patients, loved ones and staff about the impact of these two programs.

**Discussion or Conclusions**

We will discuss the lessons we have learned to this point with supporting our population and where we are looking to move toward as we continue rolling out more components of our AYA Program. We will discuss our internal and external/community partnerships and the way this enhances the support for patients.

### 2.24. Final Category: C. Community-Based and Volunteer Cancer Care Services

#### 2.24.1. 27: Exploring Knowledge User Needs and Preferences for Cancer-Specific Community-Based Insomnia Management and Support

Joshua Tulk ^1^, Stuti Patel ^1^, Linda Carlson ^2^, Sheila Garland ^3,4,5^ and Tavis Campbell ^1^
^1^ Department of Psychology, University of Calgary, Calgary, Canada;
^2^ Department of Oncology, University of Calgary, Calgary, Canada;
^3^ Department of Psychology, Memorial University, St. John’s, Canada;
^4^ Division of Oncology, Memorial University, St. John’s, Canada;
^5^ Beatrice Hunter Cancer Research Institute, Halifax, Canada.

**Background/Rationale or Objectives/Purpose**

Cancer-related insomnia is a highly prevalent and distressing symptom that negatively impacts quality of life (QOL), health outcomes, and social role engagement among survivors. Brief group educational programs have emerged as an effective, scalable, and acceptable approach to supporting the management of cancer-related insomnia. However, no comparable programs currently exist in Canada. The goal of this study is to understand the needs and preferences of cancer survivors and healthcare providers (HCPs) for community-based insomnia support in Canada.

**Methodology or Methods**

A total of 35 cancer survivors with insomnia and 4 HCPs from 6 Canadian provinces participated. Participants completed qualitative interviews regarding experiences with insomnia treatment and preferences for novel service delivery models. Interviews were transcribed and coded using hybrid inductive/deductive thematic analysis.

**Impact on Practice or Results**

Few participants reported discussing insomnia or being referred for treatment by HCPs, which was attributed to a lack of resources and training among both groups. Interview participants unanimously supported the development of new delivery models for insomnia treatment focused on providing informational support. However, views were mixed regarding who should deliver the proposed program, with more support for HCP-led compared to peer-led approaches. Suggestions for program structure and format were also reported.

**Discussion or Conclusions**

Screening and support for cancer-related insomnia is limited in Canada. Development of low-intensity educational programs to manage insomnia could support access to evidence-based interventions, improving dysfunctional beliefs, symptoms, and QOL for a sizeable number of cancer survivors. Future studies will develop and refine an educational insomnia support program through iterative testing among intended user groups.

#### 2.24.2. 52: Pilot Testing the Me for Us Group Intervention for Parents

Élodie Bergeron ^1^, Mélina Rivard ^2^, Léandra Desjardins ^3^, Juliette Bellenger ^2^, Shaneha Patel ^2^, Julie Tremblay ^1^, Annabelle Fortin ^1^ and Christine Lefebvre ^2^
^1^ Leucan, Montréal, Canada;
^2^ Université du Québec à Montréal, Montréal, Canada;
^3^ Centre de recherche du Centre Hospitalier Universitaire Sainte-Justine, Montréal, Canada.

**Background/Rationale or Objectives/Purpose**

*Me for Us* is a group intervention for parents whose young children are receiving or scheduled to receive cancer treatments. It was developed in a partnership initiative between scientists and a community organisation specialised in pediatric cancer support. Its aim is to help parents take better care of themselves while they help their children go through their illness. The intervention was created following an iterative consultation process with various experts: parents of children with cancer as well as caseworkers and scientists with expertise in pediatric cancer. *Me for US* was pilot-tested with two groups of 6 parents in autumn–winter 2024–2025 and autumn 2025.

**Methodology or Methods**

To assess program feasibility and effects, parents shared their experience during individual interviews and completed online questionnaires. These questionnaires measured their wellbeing, psychological flexibility, anxiety, depression and coping mechanisms before and after taking part in the program, as well as their satisfaction with the program.

**Impact on Practice or Results**

Parents’ feedback was positive. They expressed a high level of satisfaction with the program, and most measures showed signs of improvement (medium effect sizes).

**Discussion or Conclusions**

*Me for US* shows great promise in offering help to parents dealing their child’s cancer journey, as their needs are often overlooked by health and social services. The project team is now eager to deploy the program at a larger scale to test its efficiency in the short as well as medium term. *Me for Us* is a low-cost, promising solution to better equip parents in helping their child journey to recovery.

#### 2.24.3. 65: Bridging the Gaps: Identifying Practical and Emotional Needs in Cancer Care

Lesley Hendry ^1^, Ghizlène Sehabi ^2^, Medha Aurora ^3^ and Janet Murray ^3^
^1^ Look Good Feel Better, Toronto, Canada;
^2^ University of Ottawa, Ottawa, Canada;
^3^ LogicalOutcomes, Toronto, Canada.

**Background/Rationale or Objectives/Purpose**

To examine practical and emotional challenges affecting quality of life for individuals living with and beyond cancer and identify gaps in social support services across Canada. Secondary objectives include assessing differences in needs and available services across demographics (age, gender, ethnicity, region, socioeconomic status) and ensuring program development is guided by lived experiences.

**Methodology or Methods**

A national environmental scan identified 71 psychosocial support programs across Canada, coded across six outcome domains: physical wellbeing, emotional support, social connection, practical skills, daily living support, and navigation. Program characteristics, accessibility, and equity considerations were analyzed. Phase two will involve a national survey of individuals affected by cancer to capture priorities, barriers, and service delivery preferences. A Community Reference Group (CRG) of people with lived experience will provide guidance throughout both phases, including survey design, interpretation, and dissemination.

**Impact on Practice or Results**

Programs are concentrated in emotional support, social connection, and informational domains. Critical gaps exist in body image and identity-focused interventions, survivorship care, practical daily living support, navigation, and culturally responsive services. Geographic disparities are evident, with rural, northern, and equity-deserving populations being the least served. Virtual delivery extends reach but does not fully address accessibility, representation, or cultural relevance.

**Discussion or Conclusions**

Critical gaps exist in identity- and body-image-focused interventions, practical support, and culturally responsive programming for people living with and beyond cancer. The upcoming national survey will clarify patient priorities and guide the development of inclusive, accessible, and community- and volunteer-based psychosocial support services. Conference attendees are invited to consider the following questions: (1) How can community- and volunteer-based programs best address the most urgent psychosocial needs of diverse patient populations? (2) What strategies could enhance accessibility, inclusivity, and cultural relevance of these interventions across regions and care settings?

#### 2.24.4. 83: More than a Hand to Hold: The Importance of Role Specificity Among Cancer Care Volunteers

Carmen G. Loiselle ^1,2,3^, Laury-Ann Beaulieu Lemay ^1^ and Dinara Karimova ^1^
^1^ McGill University, Montreal, Canada;
^2^ Jewish General Hospital, Montreal, Canada;
^3^ Hope & Cope, Montreal, Canada.

**Background/Rationale or Objectives/Purpose**

Cancer volunteers provide a significant complement to professional healthcare teams by addressing the non-medical, psychosocial, and practical needs of individuals affected by cancer. However, ambiguity persists around the labeling and operationalization of their roles. Guided by Fitch’s Supportive Care Framework (SCF) and an in-depth literature search, this research clarifies the naming and nature of these roles, while providing working definitions and positioning volunteer roles according to the SCF.

**Methodology or Methods**

Three large databases and the grey literature were perused for publications from 2015 to 2025 (primarily in English) to capture the most prevalent volunteer roles, yielding 69 records. A Boolean search with relevant terms was used (e.g., *(cancer) AND (“patient navigator”) OR (“peer mentor”)*). The search was then expanded pre-2015 to find all operational definitions related to diverse volunteer roles, resulting in an additional 25 papers. Three researchers independently extracted key concepts describing volunteer roles. A refined list of concepts was compiled and related role definitions were finalized. Volunteer roles were subsequently categorized according to the SCF pyramid of needs.

**Impact on Practice or Results**

Following the extensive literature search, 29 cancer volunteer role labels were identified. Of these, 11 non-overlapping roles were consolidated. Each role was then linked to an operational definition, its associated tasks, and positioned within the SCF’s structure.

**Discussion or Conclusions**

Role clarity is essential to guide the choice of quality indicators and capture the effects of volunteer roles on patient experiences. The work presented herein constitutes an initial step in this direction. As such, role specificity will ensure that volunteers receive relevant training to fulfil their respective mandates.

#### 2.24.5. 141: Phase-Specific, Structured Virtual Peer Support Groups for Adolescents and Young Adults with Cancer

Dani Taylor.
Young Adult Cancer Canada, St. John’s, Canada.

**Background/Rationale or Objectives/Purpose**

Adolescents and young adults (AYAs) with cancer experience distinct psychosocial needs that vary across the cancer trajectory. However, peer support groups are often delivered as open or mixed-phase forums, which can unintentionally limit relevance, psychological safety, and engagement. Young Adult Cancer Canada (YACC) identified a need for a structured, phase-specific psychosocial group model tailored to diagnosis stage and developmental context.

**Methodology or Methods**

In 2023, YACC implemented a closed, diagnosis-phase-specific virtual support group program facilitated by peers with advanced mental health training. Groups meet weekly for eight consecutive weeks via videoconferencing and were limited to approximately ten participants. Three parallel streams were offered: (a) newly diagnosed or in active treatment, (b) post-treatment/survivorship, and (c) advanced or metastatic cancer. Each stream followed a structured curriculum addressing physical wellbeing, relationships, identity, mental health, and future planning, adapted to the illness phase. Groups were offered three times annually. Program evaluation included pre- and post-group participant surveys and attendance tracking.

**Impact on Practice or Results**

Following a 2022–2023 pilot (154 participant visits), the program expanded in 2023–2024, reaching 341 visits across all streams. In 2024–2025, YACC recorded 523 total participant visits. Across cohorts, attendance remained high and post-group surveys demonstrated consistently positive perceived impact, including increased connection, validation, and reduced isolation.

**Discussion or Conclusions**

Phase-specific closed groups appear to enhance engagement and psychological safety in AYA psychosocial care. Key lessons include the importance of cohort stability, skilled facilitation, and diagnosis-specific framing. Future directions include formal analysis of pre/post survey outcomes, refinement of transition pathways between group streams, and exploration of scalability and additional experience categories.

### 2.25. Final Category: D. Complementary and Integrative Cancer Care

#### 2.25.1. 8: The Role of Outdoor Activities in Supporting Mental Wellbeing During Pediatric Leukemia Treatment: An Explorative Investigation

Anguli Bharmota ^1,2^, Mariana Brussoni ^1,2^, Shahrad. Rod Rassekh ^1,2^ and Christine Voss ^1^
^1^ University of British Columbia, Vancouver, Canada;
^2^ BC Children’s Hospital Research Institute, Vancouver, Canada.

**Background/Rationale or Objectives/Purpose**

Evidence indicates that the outdoors can provide children with positive mental wellbeing effects and the ability to cope with stressful events, but little is known about use of the outdoors among pediatric cancer patients. This project is an exploratory study into understanding how pediatric leukemia patients aged 6 to 11 years old are spending time outdoors during cancer treatment, and whether outdoor time is associated with their mental wellbeing. Participant recruitment will occur through the Pediatric Oncology Clinic at BC Children’s Hospital in Vancouver, BC. Participants must be diagnosed with acute lymphoblastic leukemia (ALL) and be in the maintenance phase of treatment. This phase of treatment is when oral chemotherapy is administered but children are expected to go back to their normal routines in their communities.

**Methodology or Methods**

Semi-structured interviews will be conducted, giving participants the option for an outdoor interview at their favourite outdoor space, or a photo-elicitation interview via Zoom. In the photo-elicitation method, children will be asked before the interview to take a photo of their favourite outdoor space.

**Impact on Practice or Results**

This project will provide insight into how children with ALL spend time outdoors during treatment, and whether outdoor time is associated with mental and emotional benefits. We will examine the outdoor activities these children are engaging in, as well as any barriers they face to spending time outdoors.

**Discussion or Conclusions**

Understanding the experiences of pediatric leukemia patients can provide valuable insight for researchers, healthcare providers, and families of children with cancer, to promote mental wellbeing practices during childhood cancer treatments.

#### 2.25.2. 28: The Canadian Network for Psychedelic-Assisted Cancer Therapy (CAN-PACT): Establishing a National Foundation in Research, Clinical Practice, and Policy

Linda Carlson ^1^, Harriet Richardson ^2^, Ronald Shore ^3,4^, Christopher Albertyn ^1^, David Clements ^5^, Julie Deleemans ^1^, Elisabeth Irving ^1^, Nisha Marshall ^1^, Juliana Mendes-Rocha ^1^ and Chantal Savard ^1^
^1^ Division of Psychosocial Oncology, Department of Oncology, Cumming School of Medicine, University of Calgary, Calgary, Canada;
^2^ Sinclair Cancer Research Institute, Divisions of Canadian Cancer Trials Group and Cancer Care and Epidemiology Centre for Psychedelics Health and Research at Providence Care, Kingston, Canada;
^3^ Centre for Psychedelics Health and Research at Providence Care, Kingston, Canada;
^4^ Department of Psychiatry, Queen’s Health Sciences, Queen’s University, Kingston, Canada;
^5^ Carleton University, Ottawa, Canada.

**Background/Rationale or Objectives/Purpose**

There is growing interest and preliminary evidence for psychedelic-assisted therapy (PAT) to help people living with cancer cope with the difficulties of living with the disease, but there is a need for larger research studies to assess their efficacy and safety, the consistency of clinical education for use of psychedelics, and access to psychedelics within the current Canadian healthcare system and regulatory environment. Further, the primary objective of CAN-PACT is to address these knowledge and practice gaps for PAT integration and implementation within psychosocial oncology practice in Canada.

**Methodology or Methods**

CAN-PACT includes clinicians, researchers, patient partners, and policymakers from across multiple Canadian provinces. The network represents cancer centres, academic health science institutions, and community-based partners countrywide. A multi-phase strategy is being deployed with six major objectives:

1. Environmental scanning to map existing Canadian PAT initiatives and teams;
2. Patient-centered research priority setting using the James Lind Alliance Priority Setting Partnership model;
3. Adapting and delivering culturally responsive PAT training for healthcare professionals and educational materials for patients and families;
4. Conducting a pilot feasibility study evaluating psilocybin combined with mindfulness therapy targeting demoralization in people living with advanced cancer;
5. A planned multicenter phase III effectiveness trial within national cancer trials infrastructure;
6. Informing and influencing Canadian healthcare policy to ensure equitable and fair access to evidence-based PAT.

**Impact on Practice or Results**

The network was launched in 2025 with support from the Canadian Cancer Society, Brain Canada, and CIHR. The environmental scan finished at the end of 2025. By the time of presenting, we will have our priority setting partnership underway, have completed a PAT educational curriculum for clinicians and have commenced the pilot clinical trial.

**Discussion or Conclusions**

CAN-PACT is the first pan-Canadian initiative to address integration of PAT within psychosocial cancer care, taking a multi-layered approach across clinical trials, education, policy work, and public engagement. Its collaborative and equity-driven approach aim to enable effective, accessible, and evidence-based PAT across publicly funded cancer care settings, supporting mental and physical wellbeing for those facing advanced cancer and demoralisation.

Questions for CAPO attendees:

1. What barriers or facilitators do you foresee at your institution or within your region regarding the integration of psychedelic-assisted therapy into psychosocial oncology care, and how might they be addressed or leveraged to ensure equitable access for diverse patient populations?
2. If given the opportunity, would you personally consider training in psychedelic-assisted therapy (PAT) within your institution, and what factors would most influence your decision?

#### 2.25.3. 29: The Nature-Connected Oncology Research Exploration (N-CORE) Mixed-Method Survey: Exploring Stakeholder Perspectives of Nature-Based Interventions for People Living with and Beyond Cancer

Jamie Petersson, Julie Deleemans, Raef Mina, David Spence, Mohamed Jaffer and Linda Carlson.
University of Calgary, Calgary, Canada.

**Background/Rationale or Objectives/Purpose**

Increasing survivorship has led to a rise in chronic post-treatment gastrointestinal (GI) and psychosocial symptoms. Investigating interventions to improve these cancer-related outcomes is critical, and nature-based interventions (NBIs) have shown potential. This remains unstudied in people living with cancer (PLWC). The Nature-Connected Oncology Research Exploration (N-CORE) survey seeks to assess stakeholder attitudes and explore the relationship between nature connectedness (NC) and GI and psychosocial health in PLWC.

**Methodology or Methods**

An anonymous cross-sectional, self-report online survey of stakeholders, including 385 PLWC, 150 caregivers, 100 healthcare providers, and 50 policymakers will be conducted. Demographic and clinical data, knowledge and preferences for nature-based therapies, NC, patient-reported psychosocial and gastrointestinal health outcomes will be collected. Additionally, a qualitative interview phase will be undertaken through virtual interviews with 15 PLWC, 12 caregivers, 8 healthcare providers, and 5 policymakers. Semi-structured qualitative interview recordings will be transcribed using NVivo.

**Impact on Practice or Results**

The survey will unveil interest, attitudes, barriers, and associations between NC and health symptoms. Descriptive statistics, frequencies, correlations, and linear regression will be used to assess interest, attitudes, and barriers towards NBIs in oncology, and the relationship of nature connectedness to GI and psychosocial symptoms. Interview themes will be revealed using reflexive thematic analysis, providing a deeper understanding of stakeholder perspectives.

**Discussion or Conclusions**

The N-CORE mixed-methods survey and interviews will reveal stakeholder interest and the feasibility of NBIs for cancer care, advancing knowledge on the role of NC in improving cancer-related symptoms among PLWC. The findings will inform the development of an NBI for PLWC and caregivers.

#### 2.25.4. 34: Implementing an Intervention Combining Virtual Reality and Hypnosis in Context of Hematopoietic Stem Cell Transplantation for Hematological Malignancies: Insights from a Feasibility Study and a Randomized Trial

Audrey Laurin ^1,2^, Valentyn Fournier ^1,3,4^, Catherine Paquin ^4^, Lynda Rabia ^4^, Marine Mandon ^4^, Imran Ahmad ^4^, Sandie Oberoi ^1^, Richard Leblanc ^4^, Jean Roy ^4^ and David Ogez ^1,3^
^1^ Maisonneuve-Rosemont Hospital Research Center, Montréal, Canada;
^2^ Department of psychology, Faculty of Arts and Sciences, Université de Montréal, Montréal, Canada;
^3^ Department of Anesthesiology and Pain Medicine, Faculty of Medicine, Université de Montréal, Montréal, Canada;
^4^ Division of Hematology, Oncology and Transplantation, Maisonneuve-Rosemont Hospital and Department of Medicine, Université de Montréal, Montréal, Canada.

**Background/Rationale or Objectives/Purpose**

Hematopoietic stem cell transplantation (HSCT) is a key treatment for hematological malignancies but is associated with significant psychological burden, highlighting the need for tailored interventions to improve patients’ quality of life. Given evidence for the restorative effects of nature, we developed a virtual reality (VR) intervention that brings natural environments into the hospital room, combined with hypnosis to enhance attentional focus on positive elements. Two studies are presented: (1) a feasibility study including patient recommendations, and (2) a randomized trial evaluating the intervention’s efficacy.

**Methodology or Methods**

In the feasibility study, retention rate, frequency of VR use, and cybersickness symptoms were monitored over two weeks of autonomous headset use. Semi-structured interviews explored patients’ suggestions for improvement. The subsequent randomized trial includes six intervention sessions over two weeks, from day 7 to 21 after HSCT, with quality of life (FACT-BMT) as primary outcome.

**Impact on Practice or Results**

Feasibility results indicated a relatively low retention rate (60%), frequency of use ranging from 1 to 7, and low cybersickness. Patients feedback emphasized the preference for supervised, time-limited session rather than autonomous use. These insights informed the design of the ongoing randomized trial, for which preliminary results will be available at the time of the conference.

**Discussion or Conclusions**

This work represents one of the first interventions for patients undergoing HSCT, grounded in a structured development model. If effective, it may offer a simple, low-cost, and easily implementable approach to support patients undergoing HSCT in protective isolation.

#### 2.25.5. 123: Biofield Energetic Therapy for Supporting Psychosocial Health in People with Cancer and Caregivers: Preliminary Findings

Julie Deleemans, Katherine Jarche, Hanna Conradi, Raef Mina and Linda Carlson.
University of Calgary, Calgary, Canada.

**Background/Rationale or Objectives/Purpose**

Biofield therapies (e.g., reiki, healing touch) work with one’s physical and energetic bodies to support wellbeing. Cancer is associated with high levels of distress, adversely impacting patients and caregivers. This proof-of-concept study aims to determine whether a biofield therapy intervention (i) is feasible; and (ii) improves psychosocial health.

**Methodology or Methods**

Using a mixed-methods, pre-test–post-test, single-arm design, we are recruiting 30 people, aged ≥ 18 years, with any current or previous cancer diagnosis, or caregiver, to attend three private 60 min biofield therapy sessions with an experienced practitioner at the Arthur Child Cancer Centre in Calgary, Alberta. Validated PRO measures are collected at baseline and post-intervention for hope, depression, anxiety, post-traumatic stress, pain, spirituality, and fatigue. ESAS is collected pre-and-post each session. Optional semi-structured interviews are conducted post-intervention.

**Impact on Practice or Results**

Recruitment began in October 2025 and continues until April 2026. To date, 19 people have been screened, 15 enrolled (7 post-treatment, 5 active treatments, 2 caregivers); 6 finished the intervention with 100% compliance and data completion, 7 are ongoing, and 2 withdrew due to cancer-related illness. Preliminary data suggests the intervention is feasible and acceptable, with many participants inquiring about further sessions during their post-intervention interview. Final quantitative data will be analyzed using descriptive statistics and paired samples *t*-tests, and qualitative data via inductive thematic analysis.

**Discussion or Conclusions**

This first-of-its-kind study will explore a novel biofield therapy intervention to support people with cancer and caregivers. This intervention may yield evidence for effective, non-invasive opportunities for fostering improved psychosocial wellbeing throughout the cancer journey.

#### 2.25.6. 140: How Do Members of the Oncology Association of Naturopathic Physicians Frame the Supposed Benefits of Naturopathy in Cancer Treatment?

Giovanni Utano and Marco Zenone.
University of Ottawa, Ottawa, Canada.

**Background/Rationale or Objectives/Purpose**

The purpose of this study is to examine how naturopathic oncology practitioners frame and promote cancer-related treatment claims in online professional materials. Specifically, this study aims to identify the types of therapeutic claims presented, analyze the rhetorical and framing strategies used to construct scientific legitimacy and professional credibility, and assess the ethical and public health implications of these communication practices for patient understanding and informed decision making in complementary cancer care.

**Methodology or Methods**

A qualitative framing analysis was conducted using publicly available websites and marketing materials produced by Canadian members of the Oncology Association of Naturopathic Physicians (OncANP). Practitioners were identified through the public directory, and eligible materials were systematically collected using time-stamped screenshots and structured documentation. Cancer-related claims were extracted verbatim and classified by therapy type, claim content, and implied clinical impact using a standardized data-collection framework. Claims were assessed using a five-point problematic scale and reviewed for potential regulatory non-compliance.

**Impact on Practice or Results**

Preliminary analysis identifies recurrent frames emphasizing natural healing, immune enhancement, empowerment, and dissatisfaction with pharmaceuticals. Practitioners frequently employ scientific terminology, selective citation of research, and professional affiliations to construct credibility, while testimonials personalize claims. Many representations blur distinctions between supportive care and disease-modifying effects, creating ambiguity regarding therapeutic intent and efficacy.

**Discussion or Conclusions**

These framing practices raise ethical concerns related to patient trust and informed consent in complementary cancer care. The findings highlight the need for clearer communication standards, improved knowledge translation, and oversight to support patient safety. This work informs psychosocial oncology by clarifying how environments shape patient expectations and care choices.

#### 2.25.7. 146: Caring for the Caregiver: Development and Implementation of Oncology Caregiver Day at the Hospital for Sick Children

Fiona Penny, M.S.W, R.S.W. ^1^, Rebecca Cote, M.Sc, RN ^1^, Benita Yi, M.S.W., R.S.W. ^1^, David Brownstone, M.S.W., R.S.W. ^1^, Anna Gacsadi, RN ^1^, Megan Easton ^2^, Elana Shapiro ^2^, Megan Casey ^1^, Sonia Lucchetta, M.S.W., R.S.W. ^1,3^ and Melissa Howlett, Ph.D., Clinical and Health Psychologist ^1^
^1^ Division of Haematology and Oncology, The Hospital for Sick Children, Toronto, Canada.
^2^ Haematology/Oncology Patient and Family Advisory Council, The Hospital for Sick Children, Toronto, Canada.
^3^ Factor-Inwentash Faculty of Social Work, University of Toronto, Toronto, Canada.

**Background/Rationale or Objectives/Purpose**

Pediatric oncology caregivers experience emotional, mental, and physical burdens [1–4]. Despite evidence that caregivers desire connection, coping strategies, and psychosocial support, participation is often limited due to logistical barriers [5,6]. A needs assessment was conducted at the Hospital for Sick Children to understand and address this gap, which led to the development and implementation of an annual ‘Caregiver Day’ event.

**References**

1. Kazak, A. E., Boeving, C. A., Alderfer, M. A., Hwang, W.-T., and Reilly, A. (2005). Posttraumatic stress symptoms during treatmentinparents of children with cancer. Journal of Clinical Oncology, 23(30), 7405–7410. https://doi.org/10.1200/JCO.2005.09.110
2. Rensen, N.,Steur, L.,Grootenhuis, M.,Twisk, J., vanEijkelenburg, N., van der Sluis, I., Dors, N., van den Bos, C.,Tissing, W., Kaspers, G., and vanLitsenburg, R. (2022).Parental Sleep, Distress, and Quality of Life in Childhood Acute Lymphoblastic Leukemia: A Longitudinal Report from Diagnosis up to Three Years Later. Cancers, 14(11), 2779. https://doi.org/10.3390/cancers14112779
3. Santacroce, S. J., and Kneipp, S. M. (2020). Influence of pediatric cancer-related financial burden on parent distress and other stress-related symptoms. Pediatric Blood and Cancer,67(3), e28093. https://doi.org/10.1002/pbc.28093
4. van Warmerdam, J., Zabih, V.,Kurdyak, P., Sutradhar, R., Nathan, P. C., and Gupta, S. (2019). Prevalence of anxiety, depression, and posttraumatic stress disorder in parents of children with cancer: A meta-analysis. Pediatric Blood and Cancer, 66(6), e27677. https://doi.org/10.1002/pbc.27677
5. McGrath, P. (2001).Identifyingsupport issues ofparents of childrenwith leukemia. Cancer Practice, 9(4), 198–205. https://doi.org/10.1046/j.1523-5394.2001.94008.x
6. Paul, V.,Inhestern, L., Sigmund, D., et al. (2025). Addressing gaps and enhancing experiences in support services for families of pediatric cancer survivors. Pediatric Research, 98, 168–173. https://doi.org/10.1038/s41390-024-03320-2

**Methodology or Methods**

Fifty caregivers completed an online survey assessing caregiver needs, including desired support, content, and delivery format. Feedback highlighted the need for content specific to caregiver wellness, peer-connection, and community resources. This inspired the development of Oncology Caregiver Day; a half-day, in-person event. The first event took place in November 2024 (n = 23). Participant feedback informed program refinement and a second event was held in November 2025 (n = 30). Programming included didactic sessions promoting caregiver wellness, a caregiver panel discussion, promotion of community resources, and time dedicated to caregiver connection. Minimization of logistical barriers to participation was influential in the planning process.

**Impact on Practice or Results**

Implementation of an Oncology Caregiver Day has reinforced the importance of dedicated caregiver psychosocial interventions, co-designed with caregivers, within pediatric oncology. The event also demonstrated an appetite for in-person programming and connections.

**Discussion or Conclusions**

Caregiver feedback following the event highlighted the negative impact of unmet caregiver needs on caregiving ability, quality of life, and family outcomes. Implementation to date demonstrates strong feasibility and high demand, while reinforcing the importance of flexibility and peer connection. Future directions include ongoing refinement and expansion of caregiver programming within the Division of Haematology/Oncology.

Questions:

1. What are the common barriers to caregiver engagement and how can we adapt programming to address them?
2. How do other teams build support for investment in caregiver psychosocial support within their organizations?

#### 2.25.8. 147: From Boutique Programs to Broad Impact: Supportive and Palliative Care as Population-Level Interventions

Virginia Lee ^1,2^, Anna Burgos ^2^, Malcolm Baird ^2^, Lauryn Santiago ^2^ and Bronwen Phillips ^2^
^1^ McGill University Health Centre, Montreal, Canada;
^2^ Cedars CanSupport, Montreal, Canada.

**Background/Rationale or Objectives/Purpose**

Supportive and palliative care services including psychosocial oncology programs improve patient and caregiver outcomes and quality of cancer care. Yet, despite strong evidence, these services remain inconsistently implemented, under-resourced, and often siloed as “boutique” programs that reach only a small proportion of patients. While such models deliver excellence for some, they can perpetuate inequities. This presentation explores how supportive and psychosocial oncology services can evolve into scalable, sustainable, and equitable population-level interventions.

**Methodology or Methods**

Drawing on real-world health system experiences from the Supportive Care Programs and Cedars CanSupport at the McGill University Health Centre, this presentation will present case studies to examine strategies to shift from referral-based specialty services to system-integrated care models. Key enabling factors include stepped-care approaches, team-based, low-intensity supports for broader reach (e.g., education, digital tools, peer support, volunteers), specialty high intensity support for complex care, workforce redesign, group-based interventions, and alignment with oncology workflows.

**Impact on Practice or Results**

This session reframes supportive and psychosocial care from a “nice-to-have” adjunct to a core, essential “need-to-have” component of comprehensive cancer care delivery.

**Discussion or Conclusions**

Supportive and psychosocial care should be a foundational element of oncology for all patients, not a privilege for a few. Participants will be invited to share experiences and discuss opportunities for broader implementation across Canada.

### 2.26. Final Category: E. Exercise/Pre-Habilitation and Rehabilitation in Cancer

#### 2.26.1. 15: Implicit Belief Systems and Sexual Rehabilitation Engagement in Cancer Survivorship

Maha Khawaja ^1^, Stéphanie Gauvin ^2^ and Jessica Maxwell. ^1^
^1^ McMaster University, Hamilton, Canada;
^2^ Acadia University, Wolfville, Canada.

**Background/Rationale or Objectives/Purpose**

Sexual wellbeing is a core survivorship outcome, but psychosocial oncology rarely assesses the belief systems that shape survivors’ willingness to engage in sexual rehabilitation. This study translated Maxwell et al.’s (2017) implicit sex destiny and sex growth beliefs into an oncology context to (1) identify growth- vs. destiny-oriented belief patterns in real-world cancer survivors’ Reddit narratives and (2) examine whether growth-oriented beliefs cluster with recovery-promoting behaviours (e.g., dilation, pelvic/sexual rehabilitation, partner intimacy rebuilding), highlighting a modifiable psychosocial target for tailored survivorship care.

**Methodology or Methods**

Publicly available posts were webscraped and collected from multiple cancer-related subreddits (e.g., r/cancer, r/cervicalcancer, r/sexualhealth, r/prostatecancer, etc.) via the Python Reddit API Wrapper (PRAW). Posts were cleaned and thematically coded for linguistic markers of *sex growth beliefs* (adaptability, relearning pleasure, partner collaboration) versus *sex destiny beliefs* (permanent loss, fixed incompatibility, “sex is over”). Posts were then grouped into belief clusters and examined for co-occurring references to sexual-recovery behaviours, including vaginal dilator adherence, pelvic/sexual function exercises, and partner intimacy planning.

**Impact on Practice or Results**

Three belief clusters emerged: (1) growth-oriented, which most often described trying recommended sexual-recovery strategies and seeking partner/peer support; (2) ambivalent/mixed, which acknowledged desire to recover but anticipated failure; and (3) destiny/fixed loss, which framed sexual change as permanent and rarely mentioned rehabilitation.

**Discussion or Conclusions**

Identifying implicit sex-related belief orientations in survivor narratives offers a scalable way to flag patients who may disengage from recovery protocols. Incorporating belief-sensitive assessment into psychosocial oncology could help tailor counselling, enhance adherence, and ultimately improve post-treatment sexual quality of life.

Questions:

1. Which aspects of these belief orientations are most actionable for designing targeted psychoeducational or counselling interventions for survivors struggling with sexual recovery?
2. From a clinical or programmatic standpoint, what would be the most meaningful way to integrate belief-sensitive assessment into psychosocial oncology practice, and what barriers might clinicians face in applying this approach?

#### 2.26.2. 57: Assessing Interim Implementation Within an Ongoing Yoga Trial for Young Adults Affected by Cancer Across Canada

Emma McLaughlin ^1^, Dr. S. Nicole Culos-Reed ^1^, Hannah Cripps ^1^, Paige Boklaschuk ^2^, Heather Molina ^3^, Lauren Cowley ^4^, Kaitlyn Quinn ^5^, in memory of Lisa Currey ^5^, Maria-Hélèna Pacelli ^5^, Melissa Coombs ^5^, Sundas Shamshad ^5^, Julianna Dreger ^1^ and Dr. Amanda Wurz ^6^
^1^ Faculty of Kinesiology, University of Calgary, Calgary, Canada;
^2^ Faculty of Medicine and Health Sciences, McGill University, Montreal, Canada;
^3^ Calgary Rural Primary Care Network, Calgary, Canada;
^4^ Being Human Club, Calgary, Canada;
^5^ Participant Advisory Board, Calgary, Canada;
^6^ School of Kinesiology, University of the Fraser Valley, Chilliwack, Canada.

**Background/Rationale or Objectives/Purpose**

Based on the documented benefits of yoga across the cancer continuum, and lack of programming for young adults (YAs) diagnosed with cancer, we co-developed a 12-week yoga program for this cohort with potential for sustainability. Since September 2021, we have been testing this program in a national effectiveness–implementation trial to explore benefits and implementation. Herein, we describe interim data covering program implementation.

**Methodology or Methods**

YAs diagnosed with cancer between 18 and 39 are eligible to participate. The program consists of 60-min group-based yoga classes delivered twice weekly over Zoom. Interim implementation covers recruitment, enrollment, attendance, fidelity, adverse events, and delivery cost. In addition, quality improvement (QI) cycle data related to number of online advisory board (5 YAs [1 deceased], 2 trial staff) meetings and resultant modifications were recorded. Descriptive statistics were used to analyze interim implementation and QI data.

**Impact on Practice or Results**

As of September 2025, 130 YAs have self-referred, 80 consented, and 57 participated. Participants attended on average 17/24 (71%) sessions. Five instructors and 10 volunteers completed 216 program delivery hours costing CAD 8640 (CAD 40/hour) or CAD 152 per participant. Fidelity of program delivery ranges from 95–98% and no adverse events have been reported. Seven QI meetings led to modified recruitment strategies and revised promotional materials.

**Discussion or Conclusions**

High attendance, good fidelity, absence of adverse events, and moderate-to-low delivery costs suggest the program is feasible to deliver at scale. Ongoing efforts to secure funding and strengthen partnerships with supportive care organizations will help sustain and expand yoga for YAs, ensuring programming continues to meet the evolving needs of this population.

#### 2.26.3. 73: An Expert and Patient Driven Tailoring Process for Exercise Prescription in Neuro-Oncology

Julia T. Daun ^1,2^, Lauren C. Capozzi ^3^, Gloria Roldan Urgoiti ^4^, Meghan H. McDonough ^1^, Jessica Danyluk ^1^, Tanya Williamson ^1^, Emma McLaughlin ^1^, Paula A. Ospina ^5^, Jay C. Easaw ^6^, Margaret L. McNeely ^5,7^, George J. Francis ^4,8^, Christine Lesiuk ^4^, Paula de Robles ^4,8^, Catriona Leckie ^4^ and S. Nicole Culos-Reed ^1,9,10^
^1^ Faculty of Kinesiology, University of Calgary, Calgary, Canada;
^2^ Cumming School of Medicine, University of Calgary, Calgary, Canada;
^3^ Supportive Care, BC Cancer, Kelowna, Canada;
^4^ Department of Medical Oncology, Arthur J.E. Child Comprehensive Cancer Centre, Alberta Health Services, Calgary, Canada;
^5^ Department of Physical Therapy, University of Alberta, Edmonton, Canada;
^6^ Department of Medical Oncology, Cross Cancer Institute, Edmonton, Canada;
^7^ Supportive Care and Patient Experience, Cancer Care Alberta, Edmonton, Canada;
^8^ Department of Clinical Neurosciences, Cumming School of Medicine, University of Calgary, Calgary, Canada;
^9^ Department of Oncology, Cumming School of Medicine, University of Calgary, Calgary, Canada;
^10^ Department of Psychosocial Resources, Arthur J.E. Child Comprehensive Cancer Centre, Alberta Health Services, Calgary, Canada.

**Background/Rationale or Objectives/Purpose**

There is a need to tailor exercise interventions to address the needs of complex populations, such as neuro-oncology patients. The Alberta Cancer Exercise Neuro-Oncology (ACE-Neuro) study included a tailored approach to exercise screening, triage, prescription, and adaptation. The purpose of this work was to examine the exercise tailoring process.

**Methodology or Methods**

ACE-Neuro was a type II effectiveness–implementation exercise trial in Alberta for adult neuro-oncology patients, delivered between April 2021 and December 2023. The exercise tailoring process included (1) screening, comprising medical history review and physical examination, followed by triage to rehabilitation (physiatry, OT, PT) and/or exercise resources; (2) developing initial individualized exercise prescriptions informed by screening and triage; and (3) adapting exercise prescriptions across the intervention period based on acute patient needs. Consultation for each phase of tailoring occurred via meetings and email correspondence among clinicians (physicians, physiotherapists, nurses), exercise professionals, and researchers, with patient feedback integrated during exercise sessions.

**Impact on Practice or Results**

Screening and triage were informed by physiatrists, exercise professionals, and physiotherapists (*n* = 5). Exercise prescription involved 53 meetings (39 h) with medical oncologists, physiatrists, nurses, exercise professionals, and researchers (*n* = 12). Feedback from *n* = 59 patients informed ongoing prescription adjustments. This process resulted in an exercise prescription that considered patient function (e.g., aerobic fitness, gait), neurological impairment, exercise readiness, safety (e.g., seizure risk), disease progression, and practical factors such as transportation and ability to attend in-person and/or online.

**Discussion or Conclusions**

Expert- and patient-informed tailoring facilitated ACE-Neuro delivery. Documenting professional contributions helps to identify reproducible steps for tailored exercise and guides ongoing work to standardize exercise pathways for complex populations.

**Questions for Feedback:**

1. What clinical, psychosocial, or contextual factors most often shape exercise prescription in oncology settings?
2. What opportunities exist to strengthen patient-centred approaches to tailoring exercise in cancer care?

#### 2.26.4. 86: Examining Rural/Remote and Urban Baseline Characteristics in the EXercise for Cancer to Enhance Living Well (EXCEL) Participants: Implications for Exercise Program Delivery

S Nicole Culos-Reed ^1^, Jonathan Low ^1^, Julianna Dreger ^1^, Margaret McNeely ^2^, Melanie Keats ^3^, Daniel Santa Mina ^4^, Linda Trinh ^5^, Kristin Campbell ^6^, Isabelle Doré ^7^, Aude-Marie Foucaut ^1^, Daniel Sibley ^4^, Kelly Mackenzie ^6^, Thomas Christensen ^8^, Alexia Piché ^7^ and Olivia Mercer ^8^
^1^ Faculty of Kinesiology, University of Calgary, Calgary, Canada;
^2^ Department of Physical Therapy, Faculty of Rehabilitation Medicine, University of Alberta, Edmonton, Canada;
^3^ School of Health and Human Performance, Faculty of Health, Dalhousie University, Halifax, Canada;
^4^ Princess Margaret Cancer Centre, University Health Network, Toronto, Canada;
^5^ Faculty of Kinesiology and Physical Education, University of Toronto, Toronto, Canada;
^6^ Department of Physical Therapy, Faculty of Medicine, UBC, Vancouver, Canada;
^7^ School of Kinesiology and Science of Physical Activity, University of Montreal, Montreal, Canada;
^8^ Department of Medical Oncology, Nova Scotia Health, Halifax, Canada.

**Background/Rationale or Objectives/Purpose**

The EXercise for Cancer to Enhance Living well (EXCEL) study provides delivery of exercise oncology programs to rural/remote or underserved urban communities. The purpose of this analysis was to examine rural/remote vs. urban participant differences at baseline and understand potential implications for program delivery.

**Methodology or Methods**

Eligible adult participants with any cancer diagnosis were pre-treatment, on treatment, or up to 3 years post-treatment, lived in rural/remote (population < 100,000) or underserved urban areas, and able to participate in mild physical activity. EXCEL includes up to 12 weeks of a tailored exercise oncology program, with 60-min twice-weekly classes delivered by a qualified exercise professional.

Recruitment was coordinated through regional hub sites in Calgary, Edmonton, Halifax, Toronto, Vancouver, and Montréal with participant screening performed by Clinical Exercise Physiologists. Participant characteristics assessed at baseline included self-reported demographics, lifestyle characteristics, cancer diagnosis and treatment.

**Impact on Practice or Results**

EXCEL recruited n = 1085 rural and n = 400 urban participants between 2020 and 2025, with an average age of 60.7 (rural) vs. 57.2 years (urban). There were more female than male participants overall (79% vs. 18%; 3% not reported), and a greater relative proportion of males from rural/remote. Rural male participants were also more likely to present with metastatic disease (24.6% vs. 19.5% in urban). In contrast, urban participants were more likely to be on treatment (59.5% vs. 53.5%), more highly educated, and have lower levels of smoking and alcohol use. Physical activity levels were similar across geographic location (60% active).

**Discussion or Conclusions**

Results highlight differences between EXCEL participants at baseline based upon geographic location to consider when examining exercise delivery and effectiveness.

#### 2.26.5. 97: Improving Access to Exercise-Based Rehabilitation for People Diagnosed with Advanced Cancer: Interim Analysis of the REACH Study

Shirin M. Shallwani ^1^, Michelle Audoin ^2^, Ben Spilak ^1^, Anil Abraham Joy ^1,3^, Edith Pituskn ^1^, Kristin L. Campbell ^4^, Roanne Thomas ^5^, Sonya Lowe ^1^ and Margaret McNeely ^1^
^1^ University of Alberta, Edmonton, Canada;
^2^ Patient Partner, Cancer Rehabilitation Clinic, Toronto, Canada;
^3^ Cross Cancer Institute, Edmonton, Canada;
^4^ University of British Columbia, Vancouver, Canada;
^5^ University of Ottawa, Ottawa, Canada.

**Background/Rationale or Objectives/Purpose**

People with advanced cancer often experience symptom burden, psychosocial concerns, and functional decline. Tailored exercise-based rehabilitation (EBR) can help mitigate these concerns. The Rehabilitation and Exercise for Advanced Cancer Health (REACH) study aims to identify needs, preferences, and contextual factors influencing access to EBR among individuals with advanced cancer.

**Methodology or Methods**

Adults (≥18 years) diagnosed with advanced or metastatic cancer across Canada are recruited through cancer networks, community organizations, and social media.

This patient-oriented, mixed-methods study includes two phases of data collection:

1. Online survey examining participant characteristics, cancer-related symptoms, activity level, and access to support.
2. Focus groups exploring barriers and facilitators to accessing EBR.

**Impact on Practice or Results**

As of this interim analysis (November 2025), 84 individuals have completed the survey. Respondents are mostly cisgender women (83.3%) with a mean age of 59.3 years. Common cancer types include breast (55.9%), gynecological (13.1%), genitourinary (9.5%), and digestive (9.5%), with over half (53.6%) on active treatment. Frequent concerns include fatigue (75.0%), sleep problems (54.8%), joint issues (50.0%), and peripheral neuropathy (40.5%). Mean ESAS scores are worst for tiredness, sleep, and wellbeing (range: 3.9–4.1), and 64.3% engage in ≥30 min of physical activity at least 3 days per week. Many participants received exercise advice (64.3%) and EBR support (66.7%), while 71.4% reported barriers to accessing support. By the time of the conference, we anticipate findings from ≥140 survey responses and three focus groups (in progress).

**Discussion or Conclusions**

This presentation will identify key considerations and solutions to accessing EBR support and inform scalable, person-centered strategies to integrate EBR into supportive cancer care.

### 2.27. Final Category: F. Equity, Diversity and Inclusion (Sociodemographic, Culture, and Sex/Gender Issues)

#### 2.27.1. 14: Demographic and Clinical Profile of Patients Participating in a Group Cognitive–Behavioural Therapy for Fear of Cancer Recurrence Integrated in Routine Care

Emma Coughlan ^1,2,3^ and Josée Savard ^1,2,3^
^1^ Université Laval, Québec, Canada;
^2^ CHU de Québec-Université Laval Research Center, Québec, Canada;
^3^ Université Laval Cancer Research, Québec, Canada.

**Background/Rationale or Objectives/Purpose**

**Objectives/purpose:** This study was conducted with the aimed of identifying the demographic and clinical characteristics of patients participating in a group cognitive–behavioural therapy (CBT) for fear of cancer recurrence (FCR) in a real-world setting. This will contribute to determining who utilizes the services and, more importantly, who are the underserved subgroups.

**Methodology or Methods**

**Sample and setting:** A total of 160 participants took part in the quality-of-care assessment of the group CBT for FCR offered as part of routine care at the CHU de Québec–Université Laval. Patients were referred to a triage nurse by their cancer care provider, and when their psychosocial needs were related to FCR, the FCR group therapy was proposed to them.

**Procedures:** Participants were invited to complete questionnaires (pre- and post-treatment), either online or on paper, as part of the quality-of-care assessment. This included a sociodemographic and medical questionnaire.

**Impact on Practice or Results**

**Results:** For this study, only sociodemographic and clinical data from the pre-treatment assessment were analyzed. Most participants were middle-aged women with post-secondary education and were either employed, on sick leave, or retired. Clinically, breast cancer was the most common diagnosis, and radiation therapy was the most frequently administered treatment. The results also reveal underserved patient subgroups, including men and patients with elementary or high school education.

**Discussion or Conclusions**

**Conclusions:** Identifying the profile of patients who engage in FCR therapy in real-world settings can help tailor existing interventions to better address their needs and identify at-risk subgroups that may benefit from targeted outreach and support.

#### 2.27.2. 21: Expanding Equitable Psychosocial Cancer Care for Racialized and Neurodivergent People in Canada

Paula Holmes-Rodman, PhD ^1^, Michelle Audoin, MA ^2^ and Leah Stephenson, MA ^2^
^1^ All.Can Canada, Courtenay, Canada;
^2^ All.Can Canada, Toronto, Canada.

**Background/Rationale or Objectives/Purpose**

Canadians from underserved communities face interconnected structural barriers that negatively affect their cancer experiences and psychological wellbeing. These challenges include delayed diagnoses, higher mortality rates, lower quality of life, difficulty navigating complex care systems, and limited access to appropriate psychosocial support. This workshop highlights the experiences and innovations of individuals facing diverse and intersectional forms of marginalization to equip psychosocial oncology providers with practical strategies for delivering culturally competent, equity-oriented care, particularly for racialized and neurodivergent patients.

**Methodology or Methods**

This interactive session features the lived experiences of a Black person living with breast cancer and a caregiver supporting her autistic sister with ovarian cancer. These stories will be contextualized through findings from All.Can Canada’s research, with attention to evidence demonstrating the need for accessible, culturally appropriate psychosocial support across the cancer continuum—from early diagnostic stages to end of life.

**Impact on Practice or Results**

Participants will examine inequities in psychosocial oncology care from diagnosis through palliative and end-of-life care, informed by patient and caregiver stories of navigating and challenging systemic barriers. Through facilitated discussions, participants will identify opportunities to strengthen cultural humility, integrate equitable and community-based practices, and enhance patient-centred psychosocial support.

**Discussion or Conclusions**

1. It is important to identify and assess equity gaps in psychosocial oncology care affecting racialized and neurodivergent patients and their families.
2. It is important to learn about innovative, culturally competent resources and approaches that support diverse populations.
3. It is important to reflect on implications for professional practice and explore strategies to improve equitable psychosocial care across the cancer journey.

#### 2.27.3. 32: Enhancing Equitable Access to Cancer Care Through Guided Intake Conversations with Newly Diagnosed Patients

Erin MacDonald ^1^, Claire Link ^1^, April Wales ^1^, Ashley Mollison ^1^, Andrea DeIure ^1^ and Linda Watson ^1,2^
^1^ Cancer Care Alberta, Calgary, Canada;
^2^ University of Calgary, Calgary, Canada.

**Background/Rationale or Objectives/Purpose**

Many people face barriers to accessing cancer care. There are resources available through Cancer Care Alberta (CCA) and the community to support patients in navigating these barriers but identifying those who would benefit from an early connection to resources is a challenge. To manage this, CCA is designing provincial standardized intake questions to ask patients before their first appointment. This will help us understand who the patient is and what supports can be put in place to mitigate their barriers, including specialty navigation services for distinct patient populations.

**Methodology or Methods**

Engagement with community partners, Patient and Family Advisors, and oncology care providers guided iterative development of the intake questions. Early testing was conducted by frontline champions of this work during conversations with real patients. A pilot project facilitated additional understanding of the value of asking these questions and which barriers and associated resources were most common among patients.

**Impact on Practice or Results**

Identifying patients with complex barriers at the beginning of their care trajectory helps better equip staff to provide equity-informed, person-centered care and connect patients to relevant resources and specialty navigation as needed.

**Discussion or Conclusions**

A key learning has been that partnerships are critical to designing and implementing an equity-informed intervention like this. We have worked to partner with community-based organizations in Alberta, and with teams in other provinces doing similar equity-informed work. We also need strong partnerships across cancer care to ensure that patient navigators, clerks, oncology teams, and Allied Health providers can work together to provide tailored care.

#### 2.27.4. 38: Experiences of Online Support Group Use Among Sexual and Gender Diverse People Diagnosed with Cancer in Canada

Lauren Squires ^1,2^, Lori Ross ^1^, Aisha Lofters ^1,3^ and Jackie Bender ^1,2^
^1^ University of Toronto, Toronto, Canada;
^2^ University Health Network, Toronto, Canada;
^3^ Women’s College Hospital, Toronto, Canada.

**Background/Rationale or Objectives/Purpose**

Sexual and gender-diverse (SGD) people diagnosed with cancer navigate unique challenges and often report unmet needs. Online support groups (OSGs) may be a way for this population to access relevant peer supports to address their needs. However, little is known about their OSG experiences and whether OSGs are well-positioned to meet their needs.

**Methodology or Methods**

Participants were SGD people diagnosed with cancer recruited from across Canada both in-person at ambulatory clinics located in the Princess Margaret Cancer Centre and online. Individual 60–90-min semi-structured interviews were conducted. Data were analyzed using reflexive thematic analysis organized using Bender’s multi-theory framework of OSG use.

**Impact on Practice or Results**

A total of 19 individuals (average age = 51, 13 cisgender, 14 white, 11 breast/chest cancer) participated in interviews. Through data analysis we generated the following themes: the value of finding connection outside of/in addition to SGD identities and cancer diagnoses; OSG peers as more reliable sources of information and emotional support; trial and error when trying to fit into OSGs; and balancing the need for SGD-specific spaces with the need for critical mass.

**Discussion or Conclusions**

OSGs were able to meet participants’ needs to varying degrees. However, SGD identities were not the only similarities important to participants, and participating in SGD-specific OSGs did not necessarily guarantee satisfaction. We anticipate that insight gained from our findings will contribute to a more nuanced understanding of how OSGs can be optimized to meet the needs of SGD people diagnosed with cancer.

#### 2.27.5. 45: QUEERMUNITY: A Gender, Sex, and Relationship Diversity (GSRD) Psychotherapy Group for Cancer Patients

Margo Kennedy ^1^, Lauren Squires ^1,2^, Joseph Nimako ^3^ and Christian Schulz-Quach ^4,5,6^
^1^ Department of Supportive Care, Princess Margaret Cancer Centre, Toronto, Canada;
^2^ Dana Lana School of Public Health, University of Toronto, Toronto, Canada;
^3^ University Health Network, Toronto, Canada;
^4^ Department of Psychiatry, University of Toronto, Toronto, Canada;
^5^ Institute of Health Policy, Management and Evaluation, University of Toronto, Toronto, Canada;
^6^ Mental Health Program, University Health Network, Toronto, Canada.

**Background/Rationale or Objectives/Purpose**

Sexual and gender diverse patients with cancer (SGDc) experience documented disparities including lower social support, significant unmet psychosocial needs, and minority stress from systemic discrimination. A comprehensive needs assessment with SGDc patients (ages 18–77; diverse cancer types) revealed critical gaps: protective non-disclosure, isolation, and inadequate access to culturally affirming mental health support. Patients reported feeling “like a lone fish” and expressed urgent need for GSRD-informed therapeutic spaces addressing intersecting identities, cancer-related trauma, and queer resilience.

**Methodology or Methods**

QUEERMUNITY is an innovative in-person drop-in psychotherapy group (2nd and 4th Thursday, 90 min) grounded in Gender, Sex, and Relationship Diversity (GSRD) therapy principles and narrative/strengths-based frameworks. The intervention integrates the following approaches: (1) trauma- and grief-informed care; (2) minority stress and intersectionality theory; (3) themed psychoeducation (body image, trauma, queer shame, resilience, queer joy); and (4) peer support fostering salutogenic factors. The evaluation uses mixed methods to explore participant experience: Likert-scale outcome measures (community, belonging, coping, wellbeing) and qualitative feedback.

**Impact on Practice or Results**

Preliminary outcomes (n = 21, 15 groups January–August 2025) demonstrate strong agreement on community/belonging/safety, increased emotional wellbeing, and enhanced integration of queer identities with cancer experience. Participants developed concrete coping strategies and reported “greater sense of queer joy and pride.” The model centers SGDc identities, accessibility, and anti-oppressive practice—transferable to other oncology programs.

**Discussion or Conclusions**

Lessons learned include the following: structured facilitation enhances vulnerability; multidisciplinary/peer co-facilitation strengthens depth; environmental inclusion markers (pronouns, flags) affirm belonging. Future directions include expansion, formal effectiveness research, and integration into standard oncology psychosocial care.

#### 2.27.6. 60: EXCEL-Punjabi: Implementing a Culturally Tailored Exercise Oncology Intervention

Mannat Bansal ^1^, Sarbjit Jawandha ^2^, Chandan Sangha ^2^, Gurleen Brar ^1,2^, Jagbir Kaur ^2,3^, Tanvir C. Turin ^1^, Meghan H. McDonough ^1^ and S. Nicole Culos-Reed ^1^
^1^ University of Calgary, Calgary, Canada;
^2^ Punjabi Advisory Board, Calgary, Canada;
^3^ University of British Columbia, Vancouver, Canada.

**Background/Rationale or Objectives/Purpose**

Evaluate the implementation of a Punjabi 12-week exercise intervention delivered online or in person for individuals of South Asian heritage living with and beyond cancer.

**Methodology or Methods**

EXCEL-Punjabi is a twice-weekly culturally tailored exercise intervention consisting of progressive circuit-based aerobic, strength, balance, and flexibility exercises. Assessment included examination of referral source, reasons for non-participation, intervention adherence, facilitator delivery time, and barriers faced by participants. Punjabi-speaking individuals living with and beyond cancer and their support persons were recruited. The hybrid exercise class included online and in-person delivery at a community fitness facility (Calgary, AB). The first wave began in August 2025 and the second wave started in September 2025.

**Impact on Practice or Results**

As of November 2025, 24 participants and support persons were initially contacted either through healthcare provider referral (n = 9) or self-referral/support person referral (n = 15). 17 individuals consented (14 participants and three support persons). Barriers to consenting and enrolling included childcare commitments, work commitments, vacation plans, and not wanting others in the Punjabi community to know of their cancer. One support person and two participants dropped out. Drop out reasons included scheduling conflicts and feeling unable to exercise. Average attendance was 65%. No adverse events occurred. The instructor delivered 24 h of group exercise programming and spent 2 h per participant translating pre- and post-course questionnaires.

**Discussion or Conclusions**

These findings demonstrate that the intervention is feasible and safe. However, implementation challenges related to recruitment, scheduling, and cultural barriers need to be addressed to improve intervention uptake. Culturally tailored exercise may grant more individuals access to the benefits of exercise oncology programming.

#### 2.27.7. 63: Toward Culturally Grounded Care: Perspectives of Black Women Living with Breast Cancer in Canada

Ghizlène Sehabi, Ruth Kwizera, Anifa Kalay, Marie-Hélène Chomienne, Maria Cherba, Josephine Etowa, Mwali Muray, Anna Wilkinson, Annstein Bedeau, Malick Ouattara, Yasmine Sehabi and Sophie Lebel.
University of Ottawa, Ottawa, Canada.

**Background/Rationale or Objectives/Purpose**

In Canada, psychosocial dimensions of breast cancer among Black women remain largely understudied, both in community settings and in the experiences of the women themselves. This study aims to explore the needs, perceptions, and culturally grounded strategies used by Black women living with breast cancer as they navigate the healthcare system and seek supportive and responsive care in Canada.

**Methodology or Methods**

In partnership with a community organization, the Congolese Women’s Network, two focus groups and several individual interviews will be conducted with Black women in Canada who have lived experience of breast cancer.

**Impact on Practice or Results**

Given the lack of culturally adapted information and resources, women are expected to report challenges related to self-efficacy, unmet psychosocial needs, and limited culturally relevant supports throughout their cancer journey.

**Discussion or Conclusions**

This study will contribute to improving clinical and community practices by identifying the psychosocial and cultural needs of Black women with breast cancer in Canada, and by highlighting the importance of adaptive, culturally anchored approaches to their care. Conference attendees are invited to consider the following questions: (1) How can psychosocial oncology services be adapted to integrate culturally grounded practices that meaningfully support Black women facing breast cancer in Canada? (2) What system-level and community-based strategies can strengthen culturally responsive psychosocial support and reduce barriers to care for Black women throughout their cancer journey.

#### 2.27.8. 77: Improving Psychosocial Outcomes for Older Adults in Rural and Remote Communities: Evaluation Findings from the First 18 Months of CTAAS-BC

Robin Tonbazian ^1^, Sharon Lee ^2^, Vivian Yeung ^1^, Matthew LaRose ^2^, Carolyn Knox ^3^, Mona Rozdale ^3^, Laura Burnett ^2^ and Jennifer Martin ^4^
^1^ Canadian Cancer Society, Toronto, Canada;
^2^ Canadian Cancer Society, Hamilton, Canada;
^3^ Canadian Cancer Society, Vancouver, Canada;
^4^ Canadian Cancer Society, St. Johns, Canada.

**Background/Rationale or Objectives/Purpose**

The Canadian Cancer Society, through a partnership with the BC Government launched the Cancer Travel and Accommodation Services program in British Columbia (CTAAS-BC). Rural and remote residents, particularly older adults, face disproportionate financial, emotional, and logistical challenges accessing cancer treatment. This evaluation assessed the impact of CTAAS-BC’s supports, including travel grants, expanded transportation, and subsidized lodge accommodations, during its first 18 months.

**Methodology or Methods**

A mixed-methods evaluation (November 2024–April 2025) combined administrative data, a patient/caregiver survey (n = 956), and interest holder interviews. Rural and remote reach was mapped using Statistical Area Classification from Statistics Canada. Survey domains (treatment compliance, financial toxicity, emotional distress, and empowerment) were informed by a COSMIN-guided review of validated instruments. Quantitative data were analyzed descriptively; qualitative findings contextualized psychosocial outcomes.

**Impact on Practice or Results**

Among 9289 unique clients, 34% lived in rural and remote communities; 63% of survey respondents were aged 65+, and 55% had annual incomes below CAD 50,000. Practical supports yielded substantial out-of-pocket savings and enabled 75% of patients to continue or complete treatment. Psychosocial benefits were striking: reductions in anger (37%), depression (47%), hopelessness (49%), and worry (58%) paired with increased empowerment, as nearly half felt more optimistic and 43% enjoyed their lives despite their health challenges. These outcomes exceed overall averages, highlighting CTAAS-BC’s role in mitigating financial toxicity and emotional distress while promoting treatment adherence and resilience among older adults in rural and remote communities.

**Discussion or Conclusions**

Findings can inform provincial strategies to sustain and scale CTAAS-BC, supporting equitable, psychosocially responsive cancer care across Canada.

#### 2.27.9. 90: Co-Creating Better Gynecologic Cancer Care with Trans and Gender Diverse Communities

Tristan Bilash ^1,2^, Amanda Bolderston ^2^, Kim Meeking ^2^, Jennifer Croke ^3,4^, Christian Shultz-Quach ^3,4^, Margo Kennedy ^3,4^, Lauren Squires ^3,5^, Evan Taylor ^6^, Chelsea D’Silva ^7^ and Nazlin Jivraj ^3,4^
^1^ Cancer Care Manitoba, Winnipeg, Canada;
^2^ Queering Cancer, Centreville, Canada;
^3^ Princess Margaret Cancer Centre, Toronto, Canada;
^4^ University Health Network, Toronto, Canada;
^5^ Dalla Lana School of Public Health, Toronto, Canada;
^6^ University of Fraser Valley, Abbotsford, Canada;
^7^ Praxus Health, Calgary, Canada.

**Background/Rationale or Objectives/Purpose**

Gynecological cancer care can be a difficult, and even hostile, experience for transgender and gender-diverse (TGD) people owing to cisheteronormative environments and practices. TGD people are often diagnosed later with more advanced cancer, and experience worse health outcomes compared to the general population. Healthcare professionals (HCPs) often lack knowledge and confidence in caring for TGD people. These issues are exacerbated by a lack of research in this area.

This community-engaged project, involving people with lived experience, clinicians, researchers, and 2SLGBTQI+ community advocacy organizations, aims to address inequalities through the co-creation of patient resources, policy recommendations and HCP tools aiming to improve gynecological cancer care for TGD Canadians.

**Methodology or Methods**

A total of 20 TGD Canadians impacted by gynecological cancer will participate in semi-structured interviews. Purposive sampling will seek diversity of experiences among participants.

After a comprehensive literature search and environmental scan, an interview guide was co-developed. Trauma-informed semi-structured interviews are being conducted to understand experiences with cancer care and explore opportunities for improving the experiences for TGD people. People with lived experience are actively involved and prioritized in the recruitment strategy, interviewing, and data analysis. Through co-production, findings will inform the creation of community driven, evidence-informed resources for HCPs, patients, carers, chosen families and policy makers.

**Impact on Practice or Results**

We will share lessons learned from the project to date and preliminary themes from the interviews.

**Discussion or Conclusions**

This project addresses an identified gap for TGD communities and equips HCPs to provide more equitable and inclusive care.

1. What are some of the best practices that you’re aware of for TGD cancer care?
2. What might be possible ethical and/or theory concerns when conducting research with vulnerable groups?

#### 2.27.10. 99: A Co-Designed Nutritional Supportive Care Program for Inuit Cancer Patients and Their Families

Judi Perry Brinkert ^1^, Felicia Adelaja ^2^, Rosalie Alaralak ^3^, Juan Bermudez ^2^, Jill Burns ^4^, Beverley Dykstra ^2^, Belal El-Cheikh ^2^, Kyle Hainnu ^2^, Janet Komaksiutiksak ^3^, Moses KooNoo ^2^, Melinda Shambare ^2^, Rosie Simonee ^2^, Jemimah Thomas ^2^, Deborah Tagornak ^5^, Akumalik Tikavik ^2^, Eva Thurlow ^1^, Reyna Uriarte ^4^, Sipporah Enuaraq ^3^, Sanija Padluq ^3^ and Josee Bertrand ^6^
^1^ Wellspring Cancer Support Foundation, Toronto, Canada;
^2^ Tungasuvvingat Inuit, Ottawa, Canada;
^3^ Elder, Ottawa, Canada;
^4^ Ottawa Cancer Foundation, Ottawa, Canada;
^5^ Inuit Community Member, Ottawa, Canada;
^6^ The Ottawa Hospital, Ottawa, Canada.

**Background/Rationale or Objectives/Purpose**

Tungasuvvingat Inuit (TI) identified nutrition education as a priority need for Inuit community members affected by cancer. TI approached the Ottawa Cancer Foundation (OCF), which locally delivers Wellspring Cancer Support’s evidence based *Nourish* program, to explore cultural adaptation for urban Inuit participants facing cancer. This led to a co-design initiative utilizing a peoples-specific, self-determined approach to create a culturally adapted version of Wellspring’s Nourish program. The adapted program emphasizes the nutritional and cultural significance of traditional country foods, while also highlighting accessible nutritious foods available through local food banks. Food is approached not only as sustenance, but as an essential component of healing, identity, and community connection. In parallel, a sustainability and scalability model is being developed. The project is funded by the Canadian Partnership Against Cancer’s Models of Care Initiative.

**Methodology or Methods**

Co-design and co-implementation are guided by an Inuit Elder Advisory Group and a multidisciplinary working group including representatives from Wellspring, OCF, TI, Inuit cultural navigators, food bank staff, an Inuit chef, and a dietitian from The Ottawa Hospital Cancer Program. Evaluation methods include participant surveys, interviews, and storytelling approaches to capture both quantitative and qualitative outcomes.

**Impact on Practice or Results**

An iterative development process has been used, incorporating continuous learning and feedback. To date, an Elder-led recipe sampling session and one large community event (n = 40) have been delivered. Six additional events are planned for 2026.

**Discussion or Conclusions**

Findings will inform a replication framework, toolkit, and sustainability model to support implementation in other Inuit communities across Canada and guide future adaptations for other Indigenous populations.

1. How does this project align with other work being undertaken within the Inuit community related to cancer. Are there learnings that we can benefit from?
2. What barriers or considerations would attendees suggest we plan for in our sustainability and scalability plan?

#### 2.27.11. 105: “No One Has to Face Cancer Alone”: Wellspring Alberta’s Approach to Supporting Underserved Communities Affected by Cancer

Tana Dhruva and Niki Fehr.
Wellspring Alberta, Calgary, Canada.

**Background/Rationale or Objectives/Purpose**

Members of South Asian and Indigenous communities face unique challenges in accessing cancer support services. In South Asian communities, language barriers and cultural stigma prevent individuals from seeking or receiving cancer care. Indigenous peoples face additional barriers and poorer outcomes during a cancer experience because of racism, generational trauma, and a lack of cultural sensitivity.

Despite these challenges, there were no culturally tailored programs designed to address the unique needs of South Asian and Indigenous communities. The intended presentation will review the design approach, leadership models, and program evaluations developed by Wellspring Alberta to cultivate ethical cancer support programs for these populations, the first of their kind in Canada.

**Methodology or Methods**

Utilizing a community-based participatory approach, a needs assessment was conducted to capture unmet needs of the community and inform program development. Through focus groups and interviews, we gathered perspectives from South Asian patients, caregivers, community leaders, and healthcare providers.

In 2017 Wellspring was approached by an Indigenous Patient Navigator to develop programming for the Indigenous cancer community. Prior to program launch, 18 months were spent in a dialogue, listening and learning space with Indigenous clinicians, Indigenous peoples with lived experiences, and Elders.

**Impact on Practice or Results**

In response, Saath (“together”) Cancer Support was launched as a family-oriented, community-based program delivered in Hindi, and Punjabi. Additionally, Wellspring Alberta launched the Indigenous Cancer Sharing Circle, led by Elders and knowledge keepers.

**Discussion or Conclusions**

Ongoing tailored outreach strategies are needed to build and strengthen community relationships, raise awareness, and ensure long-term sustainability of programming.

#### 2.27.12. 110: Does Culture Matter in Treatment and Post-Cancer Recovery Among Young Adults with Cancer?

Thomas Qiao ^1^, Sharon Hou ^2^, Brianna Henry ^1^, Rachelle Drummond ^1^, Caitlin Forbes ^1^, Kyle Mendonca ^3^, Holly Wright ^4^, Iqra Rahamatullah ^5^ and Fiona Schulte ^1^
^1^ University of Calgary, Calgary, Canada;
^2^ Simon Fraser University, Burnaby, Canada;
^3^ University of Manitoba, Winnipeg, Canada;
^4^ University of Alberta, Edmonton, Canada;
^5^ Alberta Health Services, Calgary, Canada.

**Background/Rationale or Objectives/Purpose**

Young adults (18–39 years) represent a growing group within the cancer population, and Canadian healthcare has increasingly emphasized screening, assessment, treatment, and psychosocial support. However, cultural identity and related experiences remain underexamined in healthcare, despite Canada’s increasing diversity. This study aims to explore how cultural identity is perceived to influence young adults’ cancer treatment and post-cancer care experiences.

**Methodology or Methods**

As part of a large-scale mixed-methods study, young adults aged 18–39 with a cancer diagnosis were recruited across Canada to complete surveys and interviews assessing healthcare experiences and psychosocial wellbeing. A total of 98 participants (29 men, 67 women, 2 non-binary, average age 28.53 (SD = 5.25)) from 7 provinces and 1 territory completed questions related to cultural identity.

**Impact on Practice or Results**

Most participants identified as white (72.4%), followed by East Asian (10.2%), multiracial (8.1%), South Asian (5.1%), Middle Eastern (2.0%), Black (1.0%), and Latina/Latino (1.0%). Almost half (48.0%) reported that cultural identity shaped how they understood their cancer experience; 37.8% indicated that it affected follow-up care, and 13.3% reported culture-related concerns or difficulties. Over one-fifth (22.4%) expressed worries that doctors and patients misunderstand each other because of cultural differences, and 20.4% reported culture-related concerns about follow-up care.

**Discussion or Conclusions**

Cultural identity is key for many young adults with cancer and impacts both treatment and survivorship experiences. Integrating cultural identity into clinical care may reduce misunderstandings and unmet needs. Our findings will inform the refinement of clinical practices and development of guidelines to better support Canada’s diverse young adult cancer population.

#### 2.27.13. 111: Improving Psychosocial Outcomes for GBTQ+/MSM Prostate Cancer Survivors Through a Digital Survivorship Program: Mixed-Methods Findings from PCPEP Phase 4

Gabriela Ilie and Rob Rutledge.
Dalhousie University, Halifax, Canada.

**Background/Rationale or Objectives/Purpose**

GBTQ+ and Men who have Sex with Men (MSM) with prostate cancer experience disproportionately high rates of sexual dysfunction, psychological distress, and discrimination in healthcare settings—yet psychosocial oncology programs rarely address their unique needs. The Prostate Cancer Patient Empowerment Program (PCPEP) is a six-month digital survivorship intervention integrating exercise, mindfulness, pelvic floor training, intimacy support, and behavioural self-regulation. This mixed-methods study examined PCPEP’s psychosocial impact on GBTQ+/MSM participants and identified recommendations for program adaptation.

**Methodology or Methods**

Among 26 self-identified GBTQ+/MSM men enrolled in PCPEP Phase 4, fifteen completed pre/post assessments of distress (K10), anxiety (GAD-7), self-efficacy (SEMCD-6), and functional outcomes (FACT-G, EPIC domains). Twelve participated in a videoconference focus group. Analysis was guided by self-efficacy theory and behaviour-change frameworks.

**Impact on Practice or Results**

Relative to 402 heterosexual participants, GBTQ+/MSM men showed larger reductions in distress (−5.0 vs. −0.7 points) and anxiety (−3.1 vs. −1.0), with improvements in physical wellbeing (FACT-G +4.1) and smaller declines in sexual function. Qualitative findings revealed unmet needs: inclusive language, sexuality-specific guidance, navigation support for discrimination experiences, and structured opportunities for peer connection.

**Discussion or Conclusions**

GBTQ+/MSM men benefit meaningfully from digital survivorship programming yet require culturally tailored content to optimize engagement, safety, and psychosocial outcomes. Co-designing sexual-minority-specific PCPEP modules and peer-support pathways can advance equitable, identity-affirming survivorship care—addressing a long-standing gap in psychosocial oncology and improving quality of life for a historically underserved population.

#### 2.27.14. 127: Documentation of Sexual and Reproductive Health Counseling for Adolescent and Young Adult Patients Assigned Female at Birth Undergoing Pelvic Radiotherapy: A Retrospective Chart Review

Kaviya Devaraja ^1,2^, Monisha Chawla ^2^ and Anjali Sachdeva ^3^
^1^ Institute of Medical Science, University of Toronto, Toronto, Canada;
^2^ Adolescent and Young Adult Program, Department of Supportive Care, Princess Margaret Cancer Centre, University of Toronto, Toronto, Canada;
^3^ Temerty Faculty of Medicine, University of Toronto, Toronto, Canada.

**Background/Rationale or Objectives/Purpose**

Adolescent and young adult (AYA) patients assigned female at birth (AFAB) who receive pelvic radiotherapy (PRT) are at high risk for sexual and reproductive health (SRH) toxicities; however, the extent to which SRH-related discussions and referrals are documented in clinical practice remains unclear. This study aimed to evaluate the frequency, timing, nature, and comprehensiveness of documented SRH counseling (SRHC)—including discussion of key topics and referral to relevant services—for AFAB AYAs undergoing PRT at a comprehensive cancer centre.

**Methodology or Methods**

A retrospective chart review was conducted at Princess Margaret Cancer Centre. Eligible patients were ≤39 years at treatment, had a vagina, and received external beam radiotherapy or brachytherapy to the pelvis between 1 January 2017 and 31 December 2023. Seventy-six patients met the inclusion criteria.

Charts were identified through the MOSAIQ database. The extracted data included demographics, tumour and treatment characteristics, and SRHC documentation before, during, and after PRT. Documentation was reviewed across six timepoints—from initial consult to two years post-treatment—using a comprehensive list of SRH-related keywords spanning sexual function, fertility, menstrual changes, vaginal health, pelvic floor concerns, psychosocial wellbeing, and counseling or management discussions.

**Impact on Practice or Results**

Across 76 charts, SRHC documentation was inconsistent, with wide variability in frequency and timing. Preliminary analysis indicates limited documentation of sexual dysfunction, vaginal health management, fertility concerns, and distress, particularly beyond treatment completion. Counseling-related terms appeared infrequently and were rarely revisited longitudinally.

**Discussion or Conclusions**

SRHC for AFAB AYAs receiving PRT is insufficiently documented, limiting continuity of care and survivorship planning. The findings highlight the need for standardized SRHC guidelines, provider training, and improved documentation tools to support comprehensive, developmentally appropriate SRH care for AYA patients.

#### 2.27.15. 137: Maladaptive Coping and Supportive Interventions Among Asian Adults with Gastrointestinal Cancer: A Scoping Review

Toney Lieu ^1^, Lyndsey Huynh ^1^, Stephanie Huang ^2^, Lucy Ma ^3^, Michael Tau ^4,5^, Yu Qing Huang ^6,7,8^ and Eric K. Wong ^9,10^
^1^ Temerty School of Medicine, University of Toronto, Toronto, Canada;
^2^ Faculty of Medicine, University of Ottawa, Ottawa, Canada;
^3^ Division of Medical Oncology and Hematology, Princess Margaret Cancer Centre, University of Toronto, Toronto, Canada;
^4^ Geriatric Psychiatrist, Unity Health Toronto, Toronto, Canada;
^5^ Lecturer, Department of Psychiatry, University of Toronto Faculty of Medicine, Toronto, Canada;
^6^ Department of Medicine, Division of Geriatric Medicine, University of Toronto, Toronto, Canada;
^7^ Institute of Health Policy, Management, and Evaluation, University of Toronto, Toronto, Canada;
^8^ Knowledge Translation Program, Li Ka Shing Knowledge Institute, St. Michael’s Hospital, Toronto, Canada;
^9^ Knowledge Translation Program, Li Ka Shing Knowledge Institute, St. Michael’s Hospital, Unity Health Toronto, Toronto, Canada;
^10^ Division of Geriatric Medicine, Temerty Faculty of Medicine, University of Toronto, Toronto, Canada.

**Background/Rationale or Objectives/Purpose**

Gastrointestinal cancers are common in Asian populations, and their diagnosis is frequently associated with psychological distress. Coping strategies may modify psychological distress and other outcomes, but the types of coping mechanisms and ways to treat maladaptive coping are unknown. This scoping review aims to (i) explore coping strategies in Asian adults with gastrointestinal cancer; and (ii) determine the interventions for healthy coping.

**Methodology or Methods**

Asian adults aged > 18 years living in Asia or in diasporic contexts who are diagnosed with gastrointestinal cancers were included. Following PRISMA-ScR guidelines, MEDLINE, PsycINFO, Embase, and Scopus were systematically searched. Two reviewers independently screened studies. There were no restrictions for year of publication, language or setting.

**Impact on Practice or Results**

Of the 6197 records screened, 164 were included. The studies were mainly conducted in China (67.7%), South Korea (9.8%) and Taiwan (7.9%). Colorectal (59.1%), gastric (28.0%), hepatic (13.4%), and esophageal cancers (12.8%) were explored in studies. Adaptive coping strategies, such as active problem-solving, emotional acceptance, and meaning-based coping, were associated with better treatment adherence, social functioning and quality of life. Maladaptive coping strategies, including avoidance, rumination, and catastrophizing, were associated with higher psychological distress. When psychological interventions for coping were added to standard chemotherapy in patients with gastrointestinal cancer, there were improvements in cancer-related pain, overall symptom burden, side effects from chemotherapy, and mood.

**Discussion or Conclusions**

Asian adults with gastrointestinal cancer use a range of coping strategies with important implications for psychological wellbeing. Psychological interventions for coping improve outcomes during chemotherapy treatment.

#### 2.27.16. 155: QUEERMUNITY: A GSRD-Informed Queer-Affirming Psychotherapy Support Group Within a Comprehensive Cancer Centre

Alphonse Daniel ^1^, Margo Kennedy ^1^, Joseph Nimako ^1^ and Christian Schulz-Quach ^1,2,3,4^

^1^ Sexual and Gender Diversity in Cancer Care (SGDc) Program, Princess Margaret Cancer Centre, University Health Network, Toronto, Canada;
^2^ Department of Psychiatry, Temerty Faculty of Medicine, University of Toronto, Toronto, Canada;
^3^ Wilson Centre, University Health Network, University of Toronto, Toronto, Canada;
^4^ Institute of Health Policy, Management and Evaluation, University of Toronto, Toronto, Canada.

**Background/Rationale or Objectives/Purpose**

Sexually and gender-diverse people affected by cancer (SGDc) face significant health disparities and often lack access to affirming psychosocial support. Existing reviews and equity-focused frameworks have called for dedicated SGDc interventions that centre queer and trans experiences, yet few evaluated models exist in psychosocial oncology.

**Methodology or Methods**

A pre-implementation needs assessment survey of SGDc patients at Princess Margaret Cancer Centre (n = 19) assessed supportive care priorities, including interest in queer-specific groups. In response, we developed and implemented QUEERMUNITY, a queer-affirming, GSRD-informed psychotherapy support group, and invited group participants (n = 31) to complete a one-time cross-sectional post-implementation survey assessing safety, relevance, perceived impact on coping and community, and overall satisfaction, supplemented by qualitative feedback and facilitator observations.

**Impact on Practice or Results**

The needs assessment showed strong demand for queer-specific group-based supports and dedicated SGDc-specific professional supports (psychiatry/psychology, social work, and other healthcare providers). In the post-implementation survey, 95.7% endorsed the community and support, with strong recommendation of the group to others. Qualitative feedback highlighted the value of an explicitly queer-centred space for discussing cancer and queer identities.

**Discussion or Conclusions**

These findings demonstrated strong demand for queer-specific support groups and SGDc-tailored professional supports, and suggest that QUEERMUNITY is a feasible, acceptable, and highly valued model for delivering queer-affirming group-based psychosocial care in oncology. This model offers a practical, replicable, operational approach and may contribute to more equitable, affirming services for SGDc populations.

### 2.28. Final Category: H. High Tech to High Touch (Digital Health, Value-Based Care, Integrated PSO Interventions)

#### 2.28.1. 30: Personalized Cancer Care Using Patient-Reported Outcomes: The Remote SymPtom mOnitoring and Management (RESPONd) Study in Alberta

Claire Link ^1^, Chizoba Anyimukwu ^1^, Sheena Morton ^1^, Lauren Homayoon ^1^, Benedicta Asante ^1^, April Wales ^1^, Marni Armstrong ^1,2^, Andrea DeIure ^1^ and Linda Watson ^1,2^
^1^ Cancer Care Alberta, Calgary, Canada;
^2^ University of Calgary, Calgary, Canada.

**Background/Rationale or Objectives/Purpose**

Cancer Care Alberta (CCA) routinely collects Patient-Reported Outcomes (PROs) at clinic appointments using a validated measure. Many patients experience symptoms and concerns between clinic appointments, but there are limited supports beyond telephone triage calls and emergency department visits. A new study allows patients to report PROs remotely between appointments and receive support from a dedicated study nurse. This study is called REmote SymPtom MOnitoring and MaNagement (RESPONd).

**Methodology or Methods**

RESPONd implementation follows a stepped wedge design, with 6 clinics joining the study at sequential times. Recruitment is ongoing; patients in participating clinics who received systemic treatment are eligible, provided they can complete electronic PROs independently from home.

Participants are sent the PRO measure on a weekly or monthly basis. Dedicated RESPONd nurses monitor PROs reported between clinic appointments and contact patients by phone or direct message to provide symptom management support and referrals as needed. Clinic-level PROs are shared with care teams on a monthly basis.

**Impact on Practice or Results**

There are 75 patients currently active in RESPONd. Overall symptom complexity among study patients is distributed as 64% low, 29% moderate, and 7% high complexity. The study nurse has provided support through 51 phone calls and 77 direct messages and has facilitated 12 supportive care referrals to psychosocial oncology, nutrition services and physiotherapy.

**Discussion or Conclusions**

Giving patients the opportunity to report symptoms and receive support between clinic appointments can positively impact their care experience. Sharing PROs with clinic teams increases awareness of this data and its utility in informing clinical care.

#### 2.28.2. 36: Using Artificial Intelligence (AI)-Enabled Animated Videos to Enhance Patient Education and Create Clinical Capacity for Gynecological Patients in Alberta

Andrea DeIure ^1^, Amanda Jacques ^1^, Claire Link ^1^, Sophia Pin ^2^ and Tiffany Wells ^2^
^1^ Cancer Care Alberta, Calgary, Canada;
^2^ Cancer Care Alberta, Edmonton, Canada.

**Background/Rationale or Objectives/Purpose**

Cancer Care Alberta (CCA) is currently experiencing long wait times, which will continue to rise as cancer incidence is projected to grow to over 33,000 cases by 2040. Artificial Intelligence (AI) technology is increasingly being used to enhance human capabilities for accuracy, efficiency, and timely execution of administrative and educational processes. CCA has been working with clinical teams to create and implement an AI-enabled animated patient education video for gynecological patients in Alberta.

**Methodology or Methods**

Early testing was conducted with Patient and Family Advisors to assess the video’s acceptability, ease of understanding, and helpfulness. Ongoing feedback is collected through an online survey shared with endometrial cancer patients who watched the video prior to their first consultation. Survey responses help us understand if the video decreased patients’ anxiety and better prepared them for the first conversation with their oncologist.

**Impact on Practice or Results**

The goal of this video is to help patients with endometrial cancer better understand information about their cancer, its treatments, and what will happen at their consultation, before they actually attend the appointment. With this foundational knowledge, we hope that patients and families can use their first consultation appointment to have a more informed and personalized conversation about their care.

**Discussion or Conclusions**

This session will walk through how the video was created and implemented and will share feedback collected from patients who viewed the video. Videos like this provide an opportunity to create capacity for medical oncologists to spend more time on direct patient care and less time providing general background information.

#### 2.28.3. 66: Interventions for Reducing Loneliness and Social Isolation in Cancer Patients: A Systematic Review

Samantha Cheng ^1^, Layya Al Malouf ^1^, Regis Gu ^1^, Elizabeth Li ^1^, Jane Jomy ^1,2^ and Srinivas Raman ^3^
^1^ Temerty Faculty of Medicine, University of Toronto, Toronto, Canada;
^2^ Princess Margaret Cancer Centre, University Health Network, Toronto, Canada;
^3^ Department of Radiation Oncology, BC Cancer, Vancouver, Canada.

**Background/Rationale or Objectives/Purpose**

This study aims to identify and evaluate interventions designed to reduce loneliness and social isolation among adults with cancer.

**Methodology or Methods**

We searched MEDLINE, Embase, PsycINFO, CINAHL, Scopus, Web of Science, CENTRAL, and trial registries from inception to October 2025 for intervention studies reporting loneliness, social isolation, or related psychosocial outcomes among cancer patients. Eligible designs included randomized trials, quasi-experimental studies, and non-randomized interventions. Two pairs of reviewers independently screened citations and full texts, resolving discrepancies by consensus or consultation with a third reviewer. Data on study characteristics, intervention components, delivery format, and outcomes will be extracted using a standardized form. Risk of bias will be assessed using Cochrane RoB-2 and the CLARITY tool. Where appropriate, random-effects meta-analyses are planned, along with subgroup analyses by intervention type and risk of bias. Certainty of evidence will be appraised using GRADE.

**Impact on Practice or Results**

Of 2785 records imported, 523 duplicates were removed and 2262 citations were screened. A total of 191 full-text articles are undergoing review. Preliminary findings indicate substantial variation across intervention modalities, including group-based psychosocial programs, digital or telehealth supports, peer-based interventions, mind–body approaches, and exercise- or activity-based programs. Early evidence suggests several interventions improve loneliness or perceived social support, though heterogeneity and methodological limitations constrain comparability. Data extraction and synthesis are ongoing, and full results will be available prior to CAPO 2026.

**Discussion or Conclusions**

This review will synthesize current evidence on interventions to reduce loneliness and social isolation in cancer populations, clarifying promising approaches and informing future supportive oncology practice and intervention development.

#### 2.28.4. 87: Understanding the Respite Care Access Needs of Families Living with Advanced Cancers and Creating Digital Tools to Address Those Needs: A Human-Centred Design Study

Aimee Castro ^1^, John Kildea ^2^ and Argerie Tsimicalis ^2^
^1^ Université de Montréal, Montreal, Canada;
^2^ McGill University, Montreal, Canada.

**Background/Rationale or Objectives/Purpose**

Respite care benefits both patients living with advanced and terminal cancers and their family caregivers by offering short breaks from their intensive family roles while another person offers in-home accompaniment. Yet, there is a paradox of respite care in that, while it is in high demand, these services are often underutilized. Therefore, the objectives of this human-centred design study were as follows: (1) to explore the respite care needs of families coping with advanced cancers; and (2) to design digital health tools to support their needs.

**Methodology or Methods**

Sample and setting: Three adults living with advanced cancers, nine caregivers, nine nurses, and five subject-matter experts were recruited virtually through two Montreal-area hospitals and palliative community organizations.

Procedures: Iterative semi-structured interviews were conducted. Data were analyzed using content analysis techniques.

**Impact on Practice or Results**

Participants affirmed the need for respite care, particularly by families lacking social supports. Families frequently experienced difficulties in navigating and identifying appropriate community respite services. The findings informed: (1) the development of a digital navigation tool to help families seek out existing respite care agencies, and (2) the participatory design of a proof-of-concept of a smartphone app for directly coordinating in-home respite care services.

**Discussion or Conclusions**

Clinicians and researchers can use these findings to better understand the respite care needs of families living with advanced cancers and help them identify appropriate respite care services, enabling them to achieve their goals of remaining at home for as long as possible.

#### 2.28.5. 94: Development of a Digital Triage Algorithm Integrating Biomarkers and Physiological Data for CAR-T Patients

Saeed Moradian ^1^, Aghigh Ziaeemehr ^1^, Abdolreza Babamahmoodi ^2^, Shiva Ghasemi ^3^ and Babak Boutorabi ^4^
^1^ York University, Toronto, Canada
^2^ Shahidbeheshti University, Tehran, Iran
^3^ Tarbiat Modares University, Tehran, Iran
^4^ Tehran University, Tehran, Iran

**Background/Rationale or Objectives/Purpose**

Chimeric Antigen Receptor T-cell (CAR-T) therapy has transformed the treatment of hematologic malignancies. Up to 91% of CAR-T recipients experience CRS, a life-threatening immune response. Current monitoring approaches depend on intermittent assessments and symptom escalation, which may delay timely intervention. Psychologically, uncertainty about when symptoms may worsen can increase distress and cause anxiety for patients and their families.

Integrating advances biosensor technologies to measure biomarkers (IL-6, CRP, ferritin) and physiological indicators (fever, oxygen-desaturation, tachycardia) with digital health systems is now an opportunity to support early triage and proactive management.

**Methodology or Methods**

A multi-phase mixed-methods design will be employed. The algorithm will be developed based on CAR-T therapy guidelines and refined through clinician and patient input to ensure usability and accuracy. Biosensor data will be integrated with Patient-Reported Outcomes (PROs) via V-Care. User experience, adherence, and workflow integration will be assessed, along with psychological outcomes. Technology adoption and contextual factors influencing implementation will be evaluated using the CFIR framework.

**Impact on Practice or Results**

This research directly addresses a priority challenge in cancer care to improve safety and quality of life for patients undergoing advanced therapies such as CAR-T cell therapy. Early detection of CRS and timely intervention can reduce patient anxiety by ensuring continuous monitoring and reliable access to care support.

**Discussion or Conclusions**

This project highlights advancement of real-time and accurate digital triage to transform CAR-T monitoring. Increasing patients’ safety and control helps maintain or improve psychosocial wellbeing.

#### 2.28.6. 118: An Artificial Intelligence (AI) Information Agent to Empower Patients and Promote Shared Decision-Making in Lung Cancer: A Multi-Method Pilot Study

Samara Perez ^1,2,3^, Charles-Oliver Balise ^4^, Nicholas Meti ^2^, Brigitte Durieux ^2^, Ting Du ^2^, Andrew Issa ^3^, Kajamathy Subramaniam ^3^, Tibor Schuster ^2^, Georgina Lee ^5^, Juliana Herrera ^3^ and Justin Sanders ^1,2,3^
^1^ McGill University Health Centre, Montreal, Canada;
^2^ McGill University, Montreal, Canada;
^3^ Research Institute of the McGill University Health Centre, Montreal, Canada;
^4^ St Marys Hospital, Montreal, Canada.
^5^ Research Institute of the McGill University Health Centre, Montreal, Canada.

**Background/Rationale or Objectives/Purpose**

Patients with lung cancer frequently report unmet information needs about diagnosis, treatment, side-effects, and prognosis, which negatively impact anxiety, quality of life, and decision making. ALeC (AI for Lung Cancer) is an artificial intelligence (AI)-enhanced digital avatar developed to provide patients with clear, evidence-based information tailored to their cancer journey. The overall objective of this pilot study is to evaluate the usability and acceptability of ALeC among patients with lung cancer and their caregivers and to determine whether this tool supports shared decision making in cancer care. Objective 1 is to assess patient and caregiver acceptability and usability of ALeC; Objective 2 is to evaluate the clinical accuracy of AI-provided information; Objective 3 is to explore participant perspectives on ALeC’s role in supporting cancer care.

**Methodology or Methods**

This REB-approved multi-method pilot study will recruit 40 participants (20 lung cancer patients and 20 caregivers) from two cancer centres in Montreal. ALeC is HIPAA-compliant from OpenAI, AI-powered, avatar-based information agent for lung cancer that delivers up-to-date, evidence-based content sourced from National Cancer Institute materials. The AI agent provides a realistic and personalized conversational tool for patient interaction and accepts oral questions while giving verbal responses in real time (**Demo will be shown**). Participants will complete a pre-session survey, use ALeC for up to 60 min, and then complete a post-session survey, followed by a semi-structured interview. Quantitative data will assess usability and acceptability using validated satisfaction and decision-support scales. Qualitative interviews will explore participant experience, perceived value, emotional impact, and potential safety concerns related to AI-generated cancer information. Interviews will be analyzed using reflexive thematic analysis.

**Impact on Practice or Results**

Data collection is ongoing and preliminary data will be presented. Early pilot testing with our patient partners with lived experience suggest that participants find ALeC easy to use, non-judgmental, and emotionally supportive when asking difficult questions about cancer. AI information agents could be a scalable strategy to reduce unmet information needs in lung cancer and to strengthen shared decision making. Results will integrate quantitative and qualitative findings to identify design, communication, and workflow recommendations for safer and more effective AI information delivery in cancer care.

**Discussion or Conclusions**

Early findings suggest that ALeC demonstrates how high tech can enable high touch by giving patients and caregivers a safe, judgment-free space to ask difficult questions before appointments, leading to more meaningful, person-centred conversations with their oncology team. If validated, ALeC could become a scalable way to enhance shared decision making and therapeutic connection without increasing clinician workload.

1. How can AI tools best complement, rather than replace, communication with oncology teams?
2. What strategies foster patient trust and connection when introducing AI into cancer care pathways?
3. Are there differences in how patient and caregivers receive information aligned with evidence-based standards?
4. What safeguards are needed to ensure safe and responsible AI-based information delivery in cancer care?

#### 2.28.7. 124: Do the Apps Really Work? A Systematic Review and Meta-Analysis of Digital Mindfulness-Based Interventions for Cancer Survivors

Hanna Conradi, Chantal Savard and Linda Carlson.
University of Calgary, Calgary, Canada.

**Background/Rationale or Objectives/Purpose**

Cancer survivors (CSs) commonly experience persistent psychosocial symptoms (e.g., depression, anxiety, fear of cancer recurrence, fatigue, etc.) which contribute to reduced quality of life. Mindfulness-based interventions (MBIs) reduce these symptoms. However, access barriers limit participation in traditional synchronous programs. Digital health delivery offers a promising avenue to expand access. Yet, existing reviews have failed to focus on mobile-app delivered MBIs (mMBIs) in CS. Therefore, the efficacy of mMBIs for CS remains unclear.

**Methodology or Methods**

The proposed study will synthesize evidence on mMBIs for managing psychosocial symptoms in CS and meta-analyze the effects on depression, anxiety, and quality of life. The proposed methods follow established guidelines (e.g., Cochrane, PRISMA, PICO) and are registered in PROSPERO. Databases (e.g., PubMed; MEDLINE (Ovid); PsycINFO) will be searched using curated search terms for peer-reviewed studies that evaluate the psychosocial effects of an mMBI for CS. Eligible designs include randomized controlled trials as well as non-randomized, pilot, or single-group pre–post studies.

**Impact on Practice or Results**

Understanding the efficacy of mMBIs for CS, including current gaps in knowledge, will guide future directions for clinicians and researchers. Knowing which mMBIs have been tested, how, for which outcomes, and for what CS will guide the designing of further research as well as inform clinicians of the available evidence on mMBIs for CS.

**Discussion or Conclusions**

This study will address a critical gap by synthesizing and critically evaluating the effects of mMBIs for CS. The findings will inform the research, development, and implementation of mMBIs which have the potential to enhance quality of life for CS.

#### 2.28.8. 152: Targeting Cancer Patients with Treatment Disinformation Online

Marco Zenone.
University of Ottawa, Ottawa, Canada.

**Background/Rationale or Objectives/Purpose**

This presentation examines how digital platforms and online infrastructures facilitate the targeting of cancer patients with misleading or unsupported treatment claims.

**Methodology or Methods**

We draw on five empirical studies that examine how different digital platforms and online infrastructures shape cancer patients’ exposure to misleading or unsupported treatment information, including the following:

□ Audits of Google Search advertising data, examining whether alternative cancer treatment providers can target cancer-related informational queries through paid keyword advertising.
□ Content and compliance analyses of Meta (Facebook and Instagram) advertising libraries, assessing the prevalence and characteristics of ads promoting alternative cancer treatments.
□ Analyses of cancer-related medical crowdfunding campaigns drawn from major crowdfunding platforms, examining requests for non–evidence-based or alternative cancer treatments.
□ Content analyses of cancer-related books sold on Amazon, assessing the availability and nature of misleading or unsupported treatment claims in e-commerce environments.
□ Analyses of online clinic listings and patient reviews for alternative cancer clinics, examining how reputation systems and user-generated content shape clinic credibility and visibility.

**Impact on Practice or Results**

Across the five studies, we find consistent evidence that digital platforms enable the targeting and amplification of misleading or unsupported cancer treatment information. Google search advertising audits showed that alternative cancer treatment providers were able to target cancer-related informational queries, allowing ads to appear alongside or in response to searches by patients seeking legitimate medical information. Analyses of Meta advertising data identified widespread promotion of alternative cancer treatments, with many ads making implicit or explicit claims that lacked scientific support and frequently violated platform or regulatory standards. Crowdfunding analyses revealed a substantial number of cancer-related campaigns seeking funds for non-evidence-based or alternative treatments, often framed through narratives of hope, urgency, and treatment failure within conventional medicine. Content analyses of cancer-related books on Amazon demonstrated the ready availability of materials promoting unsupported or misleading treatment claims within commercial e commerce environments. Finally, analyses of online clinic listings and patient reviews showed that reputation systems and user-generated content frequently present alternative cancer clinics in a positive light, contributing to their legitimacy and visibility despite the absence of evidence for their treatment claims.

**Discussion or Conclusions**

Digital platforms function as major conduits through which cancer patients are exposed to misleading and unsupported treatment information. Across search engines, social media, e commerce platforms, crowdfunding sites, and online review systems, platform infrastructures such as advertising systems, ranking algorithms, and user-generated content features create scalable opportunities for bad actors to identify, reach, and influence patients at moments of heightened vulnerability. Rather than reflecting isolated instances of misinformation, these findings point to a structurally enabled ecosystem in which commercial incentives, limited enforcement, and opaque governance allow unsupported cancer treatment claims to persist and proliferate. These dynamics raise important concerns for patient decision making and public trust in health information, and they highlight the need for stronger platform accountability and regulatory oversight in cancer-related digital information environments.

### 2.29. Final Category: I. Implementation Science, Knowledge Translation and Synthesis

#### 2.29.1. 37: Practicing What We Preach: An Interactive Orientation to an Asynchronous E-Learning Course for People Coping with a New Cancer Diagnosis

Dana Male ^1,2^, Nicola Michaud ^3^, Amanda Jacques ^1^, Claire Link ^1^, Andrea DeIure ^1^, Marie de Guzman Wilding ^1^ and Treena Hinse ^3^
^1^ Cancer Care Alberta, Calgary, Canada;
^2^ University of Calgary, Calgary, Canada;
^3^ Cancer Care Alberta, Edmonton, Canada.

**Background/Rationale or Objectives/Purpose**

Addressing wait times in psychosocial oncology is a national priority given the increasing demand for such services and lack of commensurate clinical resources since the COVID-19 pandemic. In December 2021, Cancer Care Alberta (CCA) clinicians and researchers developed a professionally facilitated psychoeducational virtual class for patients and support people coping with a new cancer diagnosis. The pilot class demonstrated reduced distress levels and reduced the volume of patients who would have otherwise accessed more resource-intensive counselling services. These outcomes led to provincial implementation of the ‘*Coping with a New Diagnosis*’ class.

**Methodology or Methods**

Recently, CCA adapted this class to an asynchronous, interactive e-learning format. The intended benefits include the following: improved access to service through increased user convenience; ease of sharing with support people; and closed captioning in multiple languages for those with hearing impairments or with English as an Additional Language (EAL). This e-class is more sustainable than synchronous courses which rely on human resources for facilitation.

**Impact on Practice or Results**

This workshop will guide participants through an overview of the ‘*Coping with a New Diagnosis*’ e-learning modules and provide experiential learning opportunities to practice foundational skills from the course. Participants will leave with greater knowledge to support people with a new diagnosis, and awareness of this freely available resource to share or use for professional development purposes.

**Discussion or Conclusions**

Participants will explore how the information from this course may inform their practice and be adapted for their local setting. We will discuss this e-course in the context of stepped care in psychosocial oncology and consider when additional synchronous or individualized services would be recommended.

#### 2.29.2. 53: Embedding a Videoconference-Based Physical Activity Intervention Referral and Consenting Pathway into Pediatric Cancer Care

Lauren Ha ^1^, Vanessa Sales ^1^, Emma McLaughlin ^2^, Nicole Culos-Reed ^2^, Hannah Cripps ^2^, Gregory Guilcher ^3^, Sara Fisher ^4^, Caron Strahlendorf ^5^, Ewa Lunaczek-Motyka ^6^, Anne Carrelli ^6^, Raveena Ramphal ^7^, Brianna Empringham ^7^, Christopher Consmueller ^8^, Chelsea Ash ^9^, Mary Stuart ^9^, Annette Flanders ^9^ and Amanda Wurz ^1^
^1^ University of the Fraser Valley, Chilliwack, Canada;
^2^ University of Calgary, Calgary, Canada;
^3^ Alberta Children’s Hospital, Calgary, Canada;
^4^ Stollery Children’s Hospital, Edmonton, Canada;
^5^ British Columbia Children’s Hospital, Vancouver, Canada;
^6^ Victoria General Hospital, Victoria, Canada;
^7^ Children’s Hospital of Eastern Ontario, Ottawa, Canada;
^8^ Dalhousie University, Halifax, Canada;
^9^ IWK Health Centre, Halifax, Canada.

**Background/Rationale or Objectives/Purpose**

Embedding a physical activity (PA) referral and consenting pathway into pediatric cancer care requires practical strategies aligned with clinical priorities, staffing structures, and patient needs. We are collaborating with patients, survivors, carers, clinicians, and support organization partners to strengthen implementation capacity for a videoconference-delivered PA intervention (IMPACT) within pediatric cancer care across six Canadian provinces.

**Methodology or Methods**

IMPACT was initially implemented in Alberta (March 2022). Using an iterative, mixed-methods approach, we are refining referral and consenting pathways through qualitative interviews, participant meetings, and review of trial data. In British Columbia (BC), Ontario, and the Maritime provinces, IMPACT is undergoing co-adaptation (July 2025-present), with site-specific referral pathways being developed.

**Impact on Practice or Results**

In Alberta, 105 patients have been referred to IMPACT, with 27 providing informed consent. Fifteen Alberta-based clinicians have also been interviewed. These findings are guiding refinements to the existing pathway. In BC, Ontario, and the Maritime provinces, co-adaptation activities include interviews with patients/survivors (n = 1), carers (n = 2), clinicians (n = 10), and support organization partners (n = 1), alongside > 5 advisory and team meetings. Preliminary findings highlight the importance of streamlining workflows (e.g., QR code-enabled referrals), connecting to institutional consenting procedures (where applicable), and considering onsite team member presence where feasible.

**Discussion or Conclusions**

This work establishes the foundation for embedding referral and consenting pathways to enable sustained and scalable delivery of IMPACT across diverse contexts. By systematically addressing contextual determinants and co-adapting processes with patients, survivors, carers, clinicians and organization personnel, we are building capacity for future trials and advancing the broader integration of PA into pediatric cancer care.

#### 2.29.3. 68: Implementation Science in Pediatric Psychosocial Oncology: A Scoping Review of Frameworks, Strategies, and Outcomes

Harsimronjoot Sidhu ^1^, Intisar El Houjairy ^1^, Geoffrey Cuvelier ^2^, Linda Watson ^3^, Lindsay Jibb ^4^, Leandra Desjardins ^5^ and Fiona Schulte ^6^
^1^ University of Calgary, Calgary, Canada;
^2^ Section of Oncology and Transplantation, Alberta Children’s Hospital, Calgary, Canada;
^3^ Cancer Care Alberta, Alberta Health Services, Calgary, Canada;
^4^ Lawrence S. Bloomberg Faculty of Nursing, University of Toronto, Toronto, Canada;
^5^ Charles-Bruneau Cancer Center, Sainte-Justine University Health Center, Montreal, Canada;
^6^ Department of Oncology, Division of Psychosocial Oncology, Cumming School of Medicine, University of Calgary, Calgary, Canada.

**Background/Rationale or Objectives/Purpose**

Psychosocial distress is prevalent among children and adolescents with cancer, yet evidence-based psychosocial care is not consistently embedded in routine practice. This scoping review summarizes how implementation approaches have been used to support psychosocial oncology interventions in pediatric settings and identifies gaps relevant to clinical integration.

**Methodology or Methods**

We include empirical studies that report an explicit implementation framework, model, or strategy for a psychosocial intervention in pediatric (0–19 years) cancer patients and/or caregivers. Non-empirical papers, studies without an implementation component, and predominantly non-oncology populations are excluded. We followed Arksey and O’Malley’s scoping review framework. Using a PCC-based search strategy, we searched MEDLINE, PsycINFO, CINAHL, Embase, and Web of Science, identifying 9950 records. Two reviewers independently screen titles/abstracts and full texts in Covidence. Data are charted with a piloted form capturing study characteristics, psychosocial intervention type, implementation frameworks and strategies, implementation outcomes, equity-related variables, and barriers and facilitators organized against the Consolidated Framework for Implementation Research (CFIR).

**Impact on Practice or Results**

Screening and data charting are ongoing. The review will describe implementation approaches in pediatric psychosocial oncology, how often outcomes such as adoption, fidelity, sustainability, cost, and equity are examined, and commonly reported contextual barriers and enablers.

**Discussion or Conclusions**

This review will provide an overview of implementation work in pediatric psychosocial oncology, highlight areas where methods and outcome measurement are underdeveloped, and offer a reference for selecting frameworks, strategies, and outcomes. The findings will support planning for psychosocial implementation at a tertiary pediatric oncology center and may inform similar efforts elsewhere.

#### 2.29.4. 69: Mapping Determinants of Routine Distress Screening in Pediatic Oncology: CFIR-Guided Interviews with Multidisciplinary Staff

Harsimronjoot Sidhu ^1^, Geoffrey Cuvelier ^2^, Linda Watson ^3^, Lindsay Jibb ^4^, Leandra Desjardins ^5^ and Fiona Schulte ^6^
^1^ University of Calgary, Calgary, Canada;
^2^ Section of Oncology and Transplantation, Alberta Children’s Hospital, Calgary, Canada;
^3^ Cancer Care Alberta, Alberta Health Services, Calgary, Canada;
^4^ Lawrence S. Bloomberg Faculty of Nursing, University of Toronto, Toronto, Canada;
^5^ Charles-Bruneau Cancer Center, Sainte-Justine University Health Center, Montreal, Canada;
^6^ Department of Oncology, Division of Psychosocial Oncology, Cumming School of Medicine, University of Calgary, Calgary, Canada.

**Background/Rationale or Objectives/Purpose**

Routine patient-reported distress screening is recommended in pediatric oncology but is not consistently implemented. This qualitative study examines individual, workflow, and organizational factors affecting adoption of routine distress screening in a tertiary pediatric oncology program.

**Methodology or Methods**

The study occurs in the pediatric oncology program at Alberta Children’s Hospital, a tertiary center treating 70–80 new pediatric cancer cases annually. Using purposive, stratified sampling, 8–12 participants are recruited across key roles (pediatric oncologists, nurses, psychosocial clinicians, and clinic coordinators/managers). Recruitment continues to thematic saturation. Semi-structured interviews (45–60 min) are conducted in person or via secure videoconference, audio-recorded with consent, transcribed verbatim, and accompanied by field notes. An interview guide based on the Consolidated Framework for Implementation Research (CFIR) probes intervention characteristics, inner and outer setting, individual characteristics, and process. Analysis uses framework-based thematic analysis in NVivo 14 with a hybrid deductive-inductive approach: a CFIR-informed codebook is piloted and refined; initial transcripts are double-coded with intercoder agreement checks and discrepancy resolution; an audit trail and reflexive memos document analytic decisions. Credibility is supported by peer debriefing and member checking of preliminary thematic summaries.

**Impact on Practice or Results**

Data collection is underway. Analysis will report CFIR-organized themes specifying modifiable barriers and facilitators by role, role-specific contrasts, and workflow requirements for integration; outputs will include a prioritized determinant list to inform protocol design.

**Discussion or Conclusions**

This study will provide a site-specific profile of barriers and facilitators mapped to CFIR domains, identifying actionable targets for workflow design, role determination, and training to support integration of routine distress screening in pediatric oncology and to guide subsequent implementation and evaluation.

#### 2.29.5. 76: Building the CAnadian Network for Psychedelic-Assisted Cancer Therapy (CAN-PACT): Results of a Pan-Canadian Environmental Scan

Chantal Savard ^1^, Christopher P. Albertyn ^1^, Nisha Marshall ^1^, Elisabeth T. Irving ^1^, Juliana Mendes Rocha ^1^, Julie Deleemans ^1^, David Clements ^2^, Harriet Richardson ^3^, Ronald Shore ^3,4^ and Linda E. Carlson ^1^
^1^ University of Calgary, Calgary, Canada;
^2^ Carleton University, Ottawa, Canada;
^3^ Providence Care, Kingston, Canada;
^4^ Queen’s University, Kingston, Canada.

**Background/Rationale or Objectives/Purpose**

To map the Canadian landscape of psychedelic-assisted therapy (PAT) in cancer care and psychedelic mental health research and use these findings to inform the development of CAN-PACT.

**Methodology or Methods**

The scan targeted individuals and organizations engaged in PAT in oncology, palliative care, psychosocial oncology, and broader psychedelic mental health research whose expertise could support future oncology applications. Key individuals included clinicians, researchers, senior leaders, health policy and regulatory experts, students and trainees, patient partners, Indigenous representatives working in academic centers, cancer programs, and publicly funded health systems. Private, for-profit, and non-public-facing services were excluded.

A structured framework guided data collection across clinical, research, education/training, advocacy, and policy domains. Data were obtained from peer-reviewed and grey literature, clinical-trial registries, institutional websites, conference proceedings, and key-informant outreach. Key individuals were organized in a centralized database and analyzed using descriptive mapping.

**Impact on Practice or Results**

The scan identified 102 potential network members working across psychedelic-assisted oncology, broader psychedelic mental health research, and health policy. Invitations were extended to 60 prospective members who met our criteria, and 38 agreed to participate; combined with 30 individuals from the original grant submission, CAN-PACT now includes 68 members. Our members span seven provinces (BC n = 11, Alberta n = 22, Manitoba n = 3, Saskatchewan n = 1, Ontario n = 20, Quebec n = 5, NB n = 5). Engagement is most concentrated in Alberta and Ontario, highlighting opportunities to strengthen involvement across Canada.

**Discussion or Conclusions**

The environmental scan provided an empirical foundation for establishing CAN-PACT, enabling targeted invitations, initiating cross-provincial collaboration, and supporting the formation of clinical trials, education and training, and policy subcommittees. These developments position CAN-PACT to advance equitable, evidence-based psychedelic-assisted cancer care in Canada.

#### 2.29.6. 78: Implementing a Psycho-Oncology Program at VFCC: Improving Access Through Integrated Pathways and Standardized Distress Measurement

Juan Borda ^1,2^
^1^ Department of Psychiatry, Schulich School of Medicine and Dentistry, Western University, London, Canada;
^2^ Psycho-Oncology Program, Verspeeten Family Cancer Centre, London Health Sciences Centre, London, Canada.

**Background/Rationale or Objectives/Purpose**

Prior to 2025, patients at the Verspeeten Family Cancer Centre (VFCC) with mental health needs were referred externally to General Adult Ambulatory Mental Health Services (GAAMHS) by either their family physician or oncology team. This fragmented referral pathway resulted in prolonged wait times, inconsistent triage processes, and limited access to specialized psychiatric care. Although universal distress screening is widely endorsed as a core component of psychosocial oncology, its implementation at VFCC was challenged by workflow disruption, variable clinician engagement, and the absence of embedded mental health pathways. To address these gaps, a dedicated Psycho-Oncology program was implemented to strengthen screening-to-care integration, incorporating standardized distress measures (PHQ-9, GAD-7) to support early identification and longitudinal outcome monitoring.

**Methodology or Methods**

Guided by implementation science principles, the program progressively introduced a direct referral pathway within VFCC, rapid-access psychiatric assessments, structured triage algorithms, and measurement-based care using PHQ-9 and GAD-7. A controlled interrupted time series examined distress screening patterns, psychiatric referral processes, and completion of psychiatric assessments during the six months before implementation (GAAMHS-based referrals) and the six months after implementation (direct VFCC referrals).

**Impact on Practice or Results**

The integrated model was associated with more consistent identification of distress, increased referral rates, improved timeliness of psychiatric assessment, and enhanced completion of psychiatric evaluations. Routine use of PHQ-9 and GAD-7 supported diagnostic clarity, treatment planning, and longitudinal monitoring. Despite these improvements, sustained adoption of universal distress screening across clinical teams remained variable.

**Discussion or Conclusions**

Consistent with implementation science literature, embedding Psycho-Oncology services within the cancer centre improved screening-to-care integration and reduced structural barriers to access. However, broader adoption of universal distress screening continues to be limited by system-level factors. This presentation includes a brief narrative review of evidence-based implementation strategies shown to enhance sustainability of distress screening in oncology, including electronic health record integration, use of brief validated tools, hybrid electronic-paper workflows, stepped collaborative care models, staff training, workflow optimization, and ongoing patient and family education. Future directions will focus on leveraging oncology team resources to build psychotherapeutic competencies, strengthening collaborative care pathways, and developing targeted strategies to better support patients with cancer and severe persistent mental illness.

#### 2.29.7. 103: An Evidence-Based Framework for a Provincial Psychosocial Oncology Program in Nova Scotia

Katrin Julia Kaal ^1^, Anahita Hollenhorst ^2^, Logan Lawrence ^3^, Marianne Arab ^2^, Janice Howes ^2^, Mark Embrett ^3^, Joy Tarasuk ^2^, Kenneth Jacquard ^2^, Belinda Smith ^2^, Allie Campbell ^2^ and Jill Flinn ^2^
^1^ Dalhousie University/Nova Scotia Health, Halifax, Canada;
^2^ Nova Scotia Health Cancer Care Program, Halifax, Canada;
^3^ Nova Scotia Health Research, Innovation and Discovery, Halifax, Canada.

**Background/Rationale or Objectives/Purpose**

Psychosocial oncology (PSO) is an integral part of holistic, patient-centered care in oncology, and included in national oncology accreditation standards. The expansion of PSO resources in eight community oncology clinics across Nova Scotia required a comprehensive approach to organize and integrate PSO services. A multidisciplinary embedded research project conducted within the provincial cancer care program informed the development of a provincial framework for PSO with a focus on underserved populations to enable more equitable access to PSO care.

**Methodology or Methods**

A multi-phase embedded research project informed the development of the framework. Phase One encompassed rigorous evidence gathering via a scoping review of the literature and internal and external key informant interviews. Phase Two comprised two virtual workshops engaging healthcare providers/administrators, patients and community partners in discussion about findings of Phase One. Data from Phases One and Two were triangulated and synthesized. Phase Three involved knowledge dissemination in form of contextualized evidence summaries, collaborative development of core framework elements, and implementation recommendations.

**Impact on Practice or Results**

Three core elements of the framework were informed by the gathered evidence:

1. Organization, structure and philosophy: governance and reporting structure; PSO integration and communication across care teams; PSO as standard of care in cancer care.
2. Screening: distress screening.
3. Access: information and resource awareness; referral process; virtual care.

**Discussion or Conclusions**

A multidisciplinary, programmatic, complex systems approach was used to inform an evidence-based framework for a provincial model of PSO care with an emphasis on underserved populations. Implementation recommendations of the framework are guided by a phased approach outlining ‘bronze, silver and gold’ status implementation options.

#### 2.29.8. 115: Examination of Facilitating the Implementation of the Feeling Heard and Understood Scale in Canadian Cancer Care: A Multi-Site Environmental Scan

Rachael Harper ^1^, Karen Wassef ^2^, Katherine Ast ^3^, Robert Gramling ^4^, Justin Sanders ^1,2^ and Samara Perez ^1,2^
^1^ McGill University, Montreal, Canada;
^2^ McGill University Health Centre, Montreal, Canada;
^3^ American Academy of Hospice and Palliative Medicine, Chicago, USA;
^4^ University of Vermont, Burlington, USA.

**Background/Rationale or Objectives/Purpose**

Promoting health care environments where patients feel heard and understood is essential for value-concordant decision making, dignity, and the relief of suffering (Gramling et al., 2016). Although this experience is fundamental to high-quality care, it is not consistently measured in oncology and palliative care settings. The Feeling Heard and Understood (H&U) Scale was developed to address this gap as a low-burden, point-of-care measure of patients’ perceptions of clinician understanding (Edelen et al., 2022). While the scale has gained traction in several U.S. cancer centers and endorsed by The American Academy of Hospice and Palliative Medicine, to the best of our knowledge, the H&U is not being used in any Canadian hospital. Our project aims to examine the barriers and facilitators of implementing the H&U scale in Canadian oncology and palliative care contexts as a quality care measure.

**Methodology or Methods**

This environmental scan will examine how the H&U scale is currently used across 10–15 palliative and oncology programs, with a focus on understanding institutional processes, challenges, and successes. Semi-structured interviews across US institutions, e.g., City of Hope National Medical Center and University of Alabama at Birmingham, will explore patterns of implementation, perceived strengths and limitations, and system-level considerations such as cost, workflow integration, and administration logistics. Guided by the Consolidated Framework for Implementation Research (CFIR), findings will identify the key facilitators and barriers to integrating the H&U scale into routine cancer practice.

**Impact on Practice or Results**

Ongoing interviews with key stakeholders across the US (ongoing) will reveal how the H&U can be implemented e.g., systems, unexpected results of implementing, and how the measure has improved the quality of patient care. The findings will inform recommendations for the potential adoption of the H&U scale at the Cedars Cancer Centre in McGill University Health Centre (MUHC). This work aims to support the development of standardized, patient-centred communication measures within Quebec cancer care, drawing on lessons from U.S. implementations while assessing feasibility within the Canadian system.

**Discussion or Conclusions**

A clearer understanding of how the H&U scale is implemented across different institutions will support efforts to improve routine collection of patient-reported performance measures of palliative care and oncology experience in the domains of communication within Canadian cancer programs. Future directions include evaluating opportunities to integrate the H&U scale into our cancer centre’s patient satisfaction surveys.

What are the most effective strategies to overcome barriers to implementing the H&U scale in Canadian oncology and palliative care settings?

What can we draw from our US neighbours to help implement the H&U in Canadian Cancer and palliative care settings?

Should we consider ways to reward and encourage those institutions who are delivering high-quality cancer and palliative care?

#### 2.29.9. 130: A Qualitative Analysis of Patients’ Rationales for Insomnia Treatment Preferences

Nicole Carmona ^1,2^, Lucas Norton ^1^ and Karen Fergus ^1,2^
^1^ York University, Toronto, Canada;
^2^ Sunnybrook Odette Cancer Centre, Toronto, Canada.

**Background/Rationale or Objectives/Purpose**

Insomnia is a significant problem across the cancer care continuum and is effectively treated with cognitive behavioural therapy for insomnia (CBT-I). Studies have identified limited access to CBT-I in oncology settings, but have yet to assess patient preferences regarding insomnia treatment, such as *when is the best time to intervene, how, and in what format?* Using a mixed methods survey, this study seeks to answer these questions, with an eye toward how patients’ answers may differ across the cancer care continuum. To help elucidate barriers and facilitators, we conducted a preliminary qualitative analysis of patients’ reasoning for their treatment preferences at different time points.

**Methodology or Methods**

Participants will complete a virtual survey describing various insomnia treatments and respond to questions about the reasons for different levels of interest and willingness to participate in each treatment at various points in the cancer trajectory. Participants will provide textual responses to questions pertaining to why they were *more/less interested* in a particular treatment at a particular time point, and regarding treatments that are of interest, why they may be *more/less likely to use* it. Responses will be analyzed qualitatively. This preliminary analysis will include 25 participants with cancer (*N* = 25), ranging in treatment stage from diagnosis to survivorship, recruited from the Sunnybrook Odette Cancer Centre.

**Impact on Practice or Results**

We expect that, overall, participants will be **interested** in CBT-I as the gold standard insomnia treatment. However, **willingness** might fluctuate during active cancer treatment, especially if treatment involves surgery, fatigue, or high appointment burden.

**Discussion or Conclusions**

Our results of will help inform the creation of a patient-centred insomnia treatment resource that balances scientific evidence with sensitivity to patients’ challenges and reported barriers, providing nonpharmacological options that are accessible at every point in the cancer trajectory.

#### 2.29.10. 153: How Good Are Clinical Practice Guidelines in Psychosocial Oncology? A Quality Appraisal Using the AGREE-II Tool

Catherine Bergeron, Michelle Azzi, Catherine Hébert, Kia Watkins-Martin, Adina Coroiu, Carmen Loiselle, Martin Drapeau and Annett Körner.
McGill University, Montreal, Canada.

**Background/Rationale or Objectives/Purpose**

The use of science in clinical practice is critical to the provision of high-quality psychosocial oncology care; however, studies suggest that clinicians often fail to stay up to date with research and with the implementation of evidence-based approaches. Clinical practice guidelines (CPGs) are designed to address this gap by guiding the decisions of clinicians across a range of settings, including psychosocial oncology. A recent environmental scan identified 23 existing CPGs addressing the psychosocial challenges associated with cancer; however, little is known about their quality. The present study conducted a systematic quality appraisal of these CPGs.

**Methodology or Methods**

All 23 CPGs were independently evaluated by four trained appraisers using the AGREE-II tool, which consists of 23 items across six domains: scope and purpose, stakeholder involvement, rigor of development, clarity of presentation, applicability, and editorial independence. Composite domain scores (0–100%) were generated from appraiser scores, with higher scores indicating higher quality. A cutoff score of 60% was used to determine acceptable quality.

**Impact on Practice or Results**

Findings revealed that all 23 CPGs met the acceptability threshold (*M* = 76.45, *SD* = 12.35). Average scores across the six domains ranged from 33.83–96.26%, with the highest scores for clarity of presentation (*M* = 96.26, *SD* = 6.25) and scope and purpose (*M* = 87.38, *SD* = 19.55) while applicability received the lowest score (*M* = 33.83, *SD* = 10.72).

**Discussion or Conclusions**

Findings suggest that psychosocial oncology CPGs are specific and clear but may benefit from integrating more applicable tools to better support clinicians in providing psychosocial care to patients with cancer. This gap between the clarity and clinical applicability of the recommendations highlights the complexity of real-world implementation.

#### 2.29.11. 154: How Are Clinical Practice Guidelines Used and Perceived by Health Care Professionals in Psychosocial Oncology?

Catherine Bergeron, Kayla Payne, Sarah Houmad, Carmen Loiselle, Martin Drapeau and Annett Körner.
McGill University, Montreal, Canada.

**Background/Rationale or Objectives/Purpose**

Receiving a cancer diagnosis has a profound psychological impact on patients and their loved ones. However, patients frequently report that their psychosocial symptoms fail to be adequately addressed. Due to rising cancer rates and limited global investment in psychosocial oncology, there is an unprecedented demand for clinical care. Clinical practice guidelines (CPGs) are well-placed to improve the quality and efficiency of existing services; however, little is known about their use and perception in the field. The present study explored the perspectives of Canadian clinicians working in psychosocial oncology on their awareness and use of CPGs.

**Methodology or Methods**

A cross-sectional, online survey was developed to assess the use, perception, and barriers and facilitators to CPG implementation among clinicians providing direct psychosocial care in a range of settings. A multi-step, online recruitment strategy was utilized to recruit a diverse Canadian sample.

**Impact on Practice or Results**

A total of 172 participants were included in the final sample. The findings revealed low to moderate use of existing CPGs, with participants identifying a range of barriers to implementation such as a lack of formal training on CPGs, limited applicability to local contexts, and a lack of organizational support such as high workloads and limited time.

**Discussion or Conclusions**

Clinician perspectives are paramount to informing efforts to disseminate and implement psychosocial oncology CPGs. Current findings suggest that CPG implementation may be further supported by developing accessible training programs, ensuring easy access to CPGs, adapting recommendations to local healthcare contexts, and fostering greater community-based support to encourage usage of these critical tools.

### 2.30. Final Category: J. Palliative and End-of-Life Care

#### 2.30.1. 11: Clinical Study of Home-Based Palliative Care: Results of Two Studies on Representations of Home in Palliative Care

Lassagne Boris.
University Paris Nanterre, Paris, France.

**Background/Rationale or Objectives/Purpose**

Our research falls within the field of home-based palliative care. This qualitative study (SAFIR-HAD) in psychology focuses on the meaning behind patients requests to be hospitalized at home in end-of-life situations.

**Methodology or Methods**

From 2022 to 2025, we conducted 23 semi-structured research interviews with patients receiving home-based hospitalization. Two types of data analysis were performed: first, a thematic analysis of the verbatim transcripts, and second, a textual analysis using the ALCESTE analysis software.

**Impact on Practice or Results**

Our results show that the request to return home fulfills several fundamental functions for the patient. Home represents a place to protect a body that has become fragile. It is a familiar and reassuring environment that fosters connections with current family and friends, as well as with the individual’s history and past. In its own temporal dimension, home reflects a desire to endure and continue living, supporting an identity challenged by illness and hospitalizations.

**Discussion or Conclusions**

The research’s applications, particularly in the context of training healthcare professionals, explain that the request to return home should not be understood as simply a desire to live at home, but rather as the maintenance of certain habits that support a sense of continuity of being. Other applications aimed at caregivers support them in understanding the issues related to the organizational and relational upheavals of their environments and habits, because caring for a loved one at home leaves many traces that sometimes prevent effective grieving work.

#### 2.30.2. 39: Perspectives on Advance Care Planning for Adolescents and Young Adults Living with Advanced Cancer in Canada: Qualitative Insights from Patients, Caregivers, and Healthcare Providers

Emma Kearns ^1^, Mohamed Abdelaal ^2^, Marin Hoh ^1^, Sara Beattie ^1,3^, Shelly Cory ^4^, Kira Goodman ^5^, Leonie Herx ^1,6^, Brian Kelly ^1,3^, Nicole Maseja ^7^, Ursula Sansom-Daly ^8^, Fiona Schulte ^1^, Jessica Simon ^1,3^, Maya Stern ^9^, Kayla Wiebe ^3^, Lori Wiener ^10^ and Perri Tutelman ^1^
^1^ University of Calgary, Calgary, Canada;
^2^ University of Ottawa, Ottawa, Canada;
^3^ Cancer Care Alberta, Calgary, Canada;
^4^ Canadian Virtual Hospice, Winnipeg, Canada;
^5^ Canada’s Pediatric Palliative Care Alliance, Ottawa, Canada;
^6^ Rotary Flames House, Calgary, Canada;
^7^ Patient partner, Calgary, Canada;
^8^ University of New South Wales, Sydney, Australia;
^9^ Patient partner, Toronto, Canada;
^10^ National Cancer Institute, Bethesda, USA.

**Background/Rationale or Objectives/Purpose**

Adolescents and young adults (AYAs) living with advanced cancer experience unique challenges. Advance care planning (ACP) can support AYAs in sharing their care goals and preferences, but few AYAs have the opportunity to engage in these discussions. The aim of this study was to explore the perspectives and experiences of AYAs, caregivers and providers on ACP in the Canadian context.

**Methodology or Methods**

Three virtual focus groups were conducted with Canadian AYAs living with advanced cancer (*n* = 4; age range = 28–39 years), bereaved caregivers (including parents (*n* = 2) and partners (*n* = 2), and healthcare providers (HCPs; *n* = 6, including physicians (*n* = 3), nurses (*n* = 2) and a social worker (*n* = 1)). Recordings were transcribed verbatim and analyzed using reflexive thematic analysis.

**Impact on Practice or Results**

AYAs described the uncertainty associated with living with advanced cancer and expressed a strong desire to discuss their care preferences with their HCPs and families. None of the AYAs had previously engaged in ACP conversations, largely due to feeling unsure about how to express their goals and preferences and the discomfort they perceived from others related to this topic. Caregivers and HCPs supported the value of ACP but described practical challenges including a lack of knowledge and resources and discomfort with discussing ACP related to engaging in these discussions.

**Discussion or Conclusions**

ACP is an important priority for AYAs living with advanced cancer and a priority for their caregivers and healthcare providers. The findings will inform the development and evaluation of ACP tools for AYAs living with cancer in Canada and those who care for them.

#### 2.30.3. 51: How the Representation of Palliative Care in Art and Entertainment Might Impact Clinical Practice

Wilson Kwong.
Princess Margaret Cancer Centre, Toronto, Canada.

**Background/Rationale or Objectives/Purpose**

How palliative care is represented in art and entertainment invariably shapes the way patients view death and dying in real life. In many cases, patients and families’ conception of what palliative is causes a lot of psychosocial distress, even before palliative care clinicians are formally involved in their care. Understanding how the various aspects of end-of-life care are represented in the content that patients consume might help clinicians understand how to best tackle this topic in a clinical setting.

**Methodology or Methods**

A scoping review of how palliative care is represented in art and entertainment, and whether its representation is accurate. Results will be presented in a qualitative manner to outline inaccuracies in how palliative care might be represented on screen, and how this affects clinical practice.

**Impact on Practice or Results**

In general, palliative care is almost exclusively associated with death and dying in art and entertainment. The dying process tends to be marked by suffering, with little display of comfort-based interventions. Advanced care planning discussions are rarely displayed, and CPR success rates are grossly overestimated.

**Discussion or Conclusions**

Palliative care is represented in art and entertainment through a very narrow scope, with an almost exclusive focus on suffering and the dying process. This needs to be taken into consideration when palliative care is provided to patients and families, as they might have inherent biases as to what the specialty actually entails. These biases often result in psychosocial stressors prior to palliative care being formally involved, and as clinicians, understanding and addressing these biases will likely result in more seamless and effective clinical care.

#### 2.30.4. 117: Patients’ Reasoning for Accepting or Declining Early Palliative Care

Eliza Dent ^1,2^, Rachel Sue-A-Quan ^3^, Kathy Khorramak ^2^, Ashley Pope ^2^, Gary Rodin ^1,2,4^, Breffni Hannon ^1,2,5^ and Camilla Zimmermann ^1,2,5^
^1^ Institute of Medical Science, Faculty of Medicine, University of Toronto, Toronto, Canada;
^2^ Department of Supportive Care, Princess Margaret Cancer Centre, University Health Network, Toronto, Canada;
^3^ Faculty of Medicine and Dentistry, University of Alberta, Edmonton, Canada;
^4^ Department of Psychiatry, University of Toronto, Toronto, Canada;
^5^ Division of Palliative Medicine, Department of Medicine, University of Toronto, Toronto, Canada.

**Background/Rationale or Objectives/Purpose**

Early palliative care (EPC) improves quality of life for patients with advanced cancer, but is often not provided. Symptom screening with Targeted Early Palliative care (STEP) is a novel intervention in which routine symptom screening in oncology clinics triggers a call from a nurse, who offers EPC referral to patients with moderate-to-high symptoms. Here we qualitatively investigate patients’ reasons for choosing to accept or decline EPC, as offered by the nurse.

**Methodology or Methods**

Approximately 40 participants from a single-arm STEP pilot trial at the Princess Margaret Cancer Centre, Toronto will be recruited for qualitative interviews (20 accepting and 20 declining EPC). This ongoing qualitative study uses Corbin and Strauss’ grounded theory method. Consenting participants complete a semi-structured interview exploring factors they considered when deciding to accept/decline EPC. Interviews are analysed using the constant comparison approach, and categories (themes) are generated through discussion at weekly team meetings.

**Impact on Practice or Results**

To date, nine participants have completed the study: six accepted and three declined EPC referral. Patients in both groups found the nurse’s call helpful for immediate support and to explain palliative care. Reasons for EPC acceptance included: to manage symptoms; to receive personalized care; and to access extra support. Reasons for deferral included: perceiving symptoms as manageable; and a negative association with the word ‘palliative’. Past experiences and preconceived opinions about palliative care influenced decision making.

**Discussion or Conclusions**

Preliminary results indicate that while STEP is well-received, interventions directed specifically at the stigma associated with palliative care may be necessary.

### 2.31. Final Category: K. Pandemics and Cancer Care Issues

91: Improving Access to Virtual Cancer Care: Early Impact of CCS Resources on Behaviour Change Using the COM-B Model
Robin Tonbazian ^1^, Erin Bussin ^2^, Robert Howlett ^3^, Sara Schneiderman ^4^, Alana Chalmers ^1^, Rhoda Dinardo ^1^, Tracy Torchetti ^1^ and Annemarie Edwards ^1^
^1^ Canadian Cancer Society, Toronto, Canada;
^2^ Canadian Cancer Society, Vancouver, Canada;
^3^ Canadian Cancer Society, Halifax, Canada;
^4^ Canadian Cancer Society, Hamilton, Canada.

**Background/Rationale or Objectives/Purpose**

In response to the rapid shift to virtual care post-COVID-19, the Canadian Cancer Society (CCS) is leading a five-year initiative (2021–2026), funded by Merck Canada, to advance equitable virtual cancer care (VCC) across Canada. In collaboration with people living with cancer, caregivers, and healthcare providers, CCS co-designed short videos and tips to empower these audiences with tools and information to optimize virtual visits. The approach was informed by early qualitative research to ensure resources reflected real-world needs. These resources launched in June 2025 to address gaps in capability, opportunity, and motivation for effective VCC.

**Methodology or Methods**

A mixed-methods evaluation conducted between January and October 2025 assessed early impact using tracking data, a survey of 200 participants, and focus groups with 17 patients and caregivers. Survey questions were grounded in COM-B model of behaviour change.

**Impact on Practice or Results**

Within four months of launch, resources reached target audiences, with 49% of respondents living in rural or remote communities and 33% reporting annual household incomes below CAD 50,000. The findings demonstrate meaningful behaviour change: 93% reported improved knowledge of VCC best practices, 59% felt more comfortable using technology, and confidence increased for both patients (63%) and caregivers (72%). Critically, 96% took or intended to take action based on the capacity-building supports.

**Discussion or Conclusions**

These co-designed resources fill critical gaps in virtual cancer care by providing practical, evidence-informed supports that advance access and equity. Insights from this evaluation are informing a second set of resources, ensuring continuous improvement and reinforcing CCS’s commitment to patient- and caregiver-centered solutions in Canada’s evolving cancer care landscape.

### 2.32. Final Category: L. Patient Oriented Research Approaches

#### 2.32.1. 10: Setting the Top 10 Research Priorities for Psychedelic-Assisted Cancer Therapy in Canada

Nisha Marshall ^1^, Cristopher Albertyn ^1^, Chantal Savard ^1^, Hillary Horlock ^2^, Tamara Radar ^3^, Perri Tutelman ^1^, Ron Shore ^4^ and Linda Carlson ^1^
^1^ University of Calgary, Calgary, Canada;
^2^ Canadian Cancer Trials Group, Kingston, Canada;
^3^ James Lind Alliance, Southampton, United Kingdom;
^4^ Queen’s University, Kingston, Canada.

**Background/Rationale or Objectives/Purpose**

Psychedelic-Assisted Cancer Therapy (PACT) offers a promising treatment for existential anxiety, depression, and demoralization in cancer patients. Preliminary evidence suggests that PACT may be more effective than other potential treatments, though this has not been directly tested. Patient access to PACT is hindered by limited safety and efficacy data, insufficient clinical infrastructure or trained providers, and regulatory restrictions. Our objective is to conduct a priority-setting partnership (PSP) using the James Lind Alliance (JLA) methodology to determine the top unanswered research questions of patients, family caregivers, clinicians, and policymakers about PACT.

**Methodology or Methods**

This PSP is governed by a pan-Canadian expert steering committee of four patients, five clinicians, and three researchers. The JLA process involves (1) creating the steering committee (2) identifying potential partners; (3) gathering PACT-related questions from patients, caregivers, policymakers, and clinicians through an online survey and summarizing the responses; (4) checking summary questions against relevant scientific literature to determine whether they are unanswered; (5) ranking ~60 summary questions using a second online survey; (6) determining the top 10 research questions (from a short list of ~25) at a face-to-face prioritization workshop; and (7) disseminating results.

**Impact on Practice or Results**

An overview of the first round of survey responses will be presented as preliminary data.

**Discussion or Conclusions**

The JLA PSP method bridges the gap between research and the practical needs of patients, policymakers, and clinicians. The top 10 PACT research priorities will form a patient-oriented research agenda to maximize the relevance and impact of future PACT research.

Questions:

1. What methodological approaches (e.g., quota-based recruitment, weighted ranking, subgroup consensus analysis) can be used to ensure equitable representation and weighting of research priorities across stakeholder groups?
2. What steps can be taken to minimize response bias in the face-to-face prioritization workshop and how should disagreements be handled?

#### 2.32.2. 33: Creating a Workspace That Is Responsive to Member and Project Needs in a Patient–Scientist Collaboration

Sevtap Savas ^1,2^ and Georgia Skardasi ^1,2^
^1^ Atlantic Cancer Consortium Patient Advisory Committee, St. John’s, Canada;
^2^ Memorial University of Newfoundland, St. John’s, Canada.

**Background/Rationale or Objectives/Purpose**

Patient–scientist collaborations can approach problems in new ways and develop novel solutions by combining patients’ and scientists’ knowledge, skills, and expertise. However, views and clarity around member roles, project management, and deliverables may differ, creating conflicts that need to be resolved for creating an effective workspace.

**Objective:** To describe the issues that we faced and solutions that we implemented while collaborating within the Atlantic Cancer Consortium Patient Advisory Committee (ACC PAC).

**Methodology or Methods**

We reviewed recent meeting minutes, email correspondences, annual patient member feedback survey responses (2025), and one online comment box entry.

**Impact on Practice or Results**

The ACC PAC currently involves 11 patient advisors and four academic members. Not every advisor raised concerns. The issues identified included unclarity around advisor roles, project objectives, and obligations, as well as scheduling conflicts resulting to some advisors being unable to attend all meetings and hence feeling unheard. As a response, the committee had two meetings specifically focusing on issues raised, clarifications, and solutions. The committee also implemented a comment box that can be used to provide feedback anytime, started using a different scheduling tool, is offering additional meeting opportunities, and is talking more frequently about project objectives, obligations, constraints, and advisor roles and needs.

**Discussion or Conclusions**

It is not unusual to have differing needs in a collaboration that brings together members with different circumstances, however continuous care to address these needs is required. In patient–scientist collaborations, being reflective, flexible and responsive to conflicts arising between project obligations and member needs can help address issues.

#### 2.32.3. 46: Hope and Loneliness Among Individuals Affected by Biliary Tract Cancers: Findings from the Canadian Cholangiocarcinoma Collaborative (C3)

Samar Attieh ^1^, Christine Lafontaine ^1^, Leonard Angka ^1^, Melinda Bachini ^2^, Rebecca Auer ^1^ and Carmen G. Loiselle ^3,4^
^1^ Cancer Research Program, Ottawa Hospital Research Institute, Ottawa, Canada;
^2^ Cholangiocarcinoma Foundation, Riverton, USA;
^3^ Department of Oncology and Ingram School of Nursing, Faculty of Medicine and Health Sciences, McGill University, Montreal, Canada;
^4^ Segal Cancer Center, Jewish General Hospital, Montreal, Canada.

**Background/Rationale or Objectives/Purpose**

Hope and loneliness are key factors that may affect coping with cancer. For those diagnosed with biliary tract cancers (BTCs), maintaining hope and avoiding loneliness is particularly crucial due to the cancers’ rarity, poor prognosis, and limited support. The Canadian Cholangiocarcinoma Collaborative (C3) was established to address these challenges through system navigation, access to genomic testing and clinical trials, and support. This study aimed to measure how hope and loneliness may change over time as participants engage with C3 initiatives.

**Methodology or Methods**

A total of 92 patients and 44 informal caregivers, who joined C3 via self, peer, or physician referrals, consented to participate in the Hope study and were invited to complete e-self-reported measures of hope (Herth Hope Index, 12 items) and loneliness (UCLA Loneliness Scale, 20 items) at baseline (T0), 2 weeks after the first C3 informational session (T1), and after 2 to 3 months (T2).

**Impact on Practice or Results**

At T0, high hope scores were found (patients: M = 39.8, SD = 4.9; caregivers: M = 38.9, SD = 4.68) with low-to-moderate loneliness ratings (patients: M = 32.13, SD = 9.63; caregivers: M = 36.69, SD = 12.37). At T1, a significant decrease in hope was noted among patients (Mean Difference = −1.38, 95% CI [−2.64, −0.11], *p* = 0.029, and an increase in loneliness (MD = 1.75, 95% CI [0.27, 3.23], *p* = 0.016), with no significant change from T1 to T2. Among caregivers, no significant changes were found at T1, while a significant decrease in hope MD = −2.01, 95% CI [−3.61, −0.40], *p* = 0.010) and an increase in loneliness MD = 4.54, 95% CI [1.31, 7.77], *p* = 0.004 were found at T2.

**Discussion or Conclusions**

Findings serve to inform ongoing and future C3 initiatives to provide timely personalized support to affected individuals.

#### 2.32.4. 47: Exploring the Ramifications of Being Diagnosed with a Rare Cancer Among Patients and Informal Caregivers Affected by Biliary Tract Cancer: A Focus on Hope and Lived Experiences

Samar Attieh ^1^, Christine Lafontaine ^1^, Leonard Angka ^1^, Melinda Bachini ^2^, Rebecca Auer ^1^ and Carmen G. Loiselle ^3,4^
^1^ Cancer Research Program, Ottawa Hospital Research Institute, Ottawa, Canada;
^2^ Cholangiocarcinoma Foundation, Riverton, USA;
^3^ Department of Oncology and Ingram School of Nursing, Faculty of Medicine and Health Sciences, McGill University, Montreal, Canada;
^4^ Segal Cancer Center, Jewish General Hospital, Montreal, Canada.

**Background/Rationale or Objectives/Purpose**

Biliary tract cancers (BTCs) are rare malignancies associated with poor prognosis, limited treatment options, and significant psychosocial distress among affected individuals. The Canadian Cholangiocarcinoma Collaborative (C3) was established to enhance access to treatment and support. Using a mixed-methods design, the C3 Hope study aimed to longitudinally assess hope and loneliness among C3 patients and their caregivers and explore their lived experiences in more depth.

**Methodology or Methods**

A total of 92 BTC patients and 44 informal caregivers consented to participate in the Hope study. Seven dyads of patients and their caregivers participated in virtual focus groups. A semi-structured interview guide was used, and sessions were video-recorded and transcribed verbatim. Key themes will be identified through thematic analysis.

**Impact on Practice or Results**

Focus groups included patients (mean age = 59.86 years) and caregivers (mean age = 56.29 years), from six Canadian provinces: Alberta, British Columbia, Ontario, Manitoba, New Brunswick, and Newfoundland and Labrador. Four of the seven patients were male; five of the seven caregivers were female. Four patients reported an intrahepatic cholangiocarcinoma diagnosis, and three were unsure of the BTC subtype. Six patients were diagnosed in 2024–2025; four had undergone tumor resection and were receiving treatment. Caregivers were predominantly spouses. Participants reported high levels of hope and low-to-moderate loneliness. The thematic analysis is in progress, and results will be presented at CAPO 2026.

**Discussion or Conclusions**

Focus groups’ findings will provide an in-depth understanding of the lived experience of both patients and caregivers affected by BTC, as well as inform ongoing and future C3 initiatives, including advocacy and research.

#### 2.32.5. 71: More than the Kilometres Travelled: Uncovering the Psychosocial Impact of Travel to Treatment for Canadians Living with Blood Cancer

Christina Sit, Deidra Brown-John, Kristin Dyck, Nadine Frisk, Brandon Peel, Brooke Dewhurst and Colleen McMillan.
Leukemia and Lymphoma Society of Canada, Toronto, Canada.

**Background/Rationale or Objectives/Purpose**

- To examine the financial, physical, and psychosocial impacts of travel to treatment for Canadians diagnosed with blood cancer, their caregivers, and families;
- To identify potential differences between communities and regions.

**Methodology or Methods**

A national online survey (November 2024–January 2025), co-developed by a six-member Steering Committee including four individuals with lived experience, collected responses from 454 Canadians who experienced and blood cancer and caregivers across all provinces who had travelled outside their home city for care.

**Impact on Practice or Results**

Of the respondents, 59.7% traveled over 100 km, and 50.2% had been travelling for two years or more. Due to the need for travel, 48.8% experienced an income reduction of 10% or more; 51.7% reported persistent worry; 41.0% reported anxiety; 32.1% reported guilt related to travel; 48.6% lived apart from their immediate family, with 52.8% separated for three months or longer; 63.4% reported significant–severe stress from living apart from family; 58.1% experienced strain in partner or marital relationships.

**Discussion or Conclusions**

Travel to treatment impacts far more than time and distance. The psychosocial impact is broad and affects emotional wellbeing and family stability. Policies must reduce travel burdens and embed psychosocial supports to achieve equitable, patient-centred cancer care across Canada.

#### 2.32.6. 75: A Five-Year Account of the Achievements of the Public Interest Group on Cancer

Sevtap Savas ^1,2^, John King ^3^, Holly Etchegary ^1,2^, Derrick Bishop ^1^, Janine Taylor-Cutting ^1^, Jason Wiseman ^1^, Justin Andrews ^1^, Darrell Peddle ^1^ and Kimberly Voisey ^1^
^1^ Public Interest Group on Cancer, St. John’s, Canada;
^2^ Memorial University of Newfoundland, St. John’s, Canada;

**Background/Rationale or Objectives/Purpose**

Collaborations between public members and academics can generate innovative solutions for people and families affected by cancer. The Public Interest Group on Cancer (PI group) in Newfoundland and Labrador (NL) is a recognized partnership focused on cancer research, public engagement, and advocacy.

To describe the main initiatives, outcomes, and accomplishments of the PI group and present its future goals.

**Methodology or Methods**

We reviewed our proposals, outputs, accolades, and meeting minutes and summarized key group activities.

**Impact on Practice or Results**

The PI Group started in 2021 with 18 volunteers and CAD 2500 in funding. Since its inception, the PI Group has received funding for six projects, initiated a podcast dedicated to cancer (with 28 episodes to date), and published four scholarly and several public articles. We also organized two free public events on cancer and frequently advocated with policy makers and in the media for people and families affected by cancer. In 2024, our group received the President’s Award for Public Engagement from Memorial University. In 2025, the group’s success and impact were recognized when the group lead received a national leadership award. The PI Group is currently working on a project to profile a vision of the future of cancer in NL.

**Discussion or Conclusions**

The PI Group represents a successful patient–scientist collaboration on cancer. It has been evolving its activities, with various accomplishments and impactful roles in both public and academic settings. With its operations, accomplishments, and future plans, the PI Group may inspire others to create similar public-academic partnerships on cancer.

#### 2.32.7. 82: Co-Creating Young Adult Yoga (YAY): Partnership Processes and Outputs from a Community-Researcher Collaboration

Amanda Wurz ^1^, Reegan Garret ^2^, Eden Baerg ^1^, Paige Boklaschuk ^3^, Hannah Nette ^2^, Christine Simmons ^4^, Nathalie LeVasseur ^4^, Maxime Caru ^5^ and Patient Partners (Melissa Coombs, Sundas Sham, Liz Greenslade, anonymous) ^1^
^1^ University of the Fraser Valley, Chilliwack, Canada;
^2^ InspireHealth, Vancouver, Canada;
^3^ McGill University, Montreal, Canada;
^4^ BC Cancer, Vancouver, Canada;
^5^ The Pennsylvania State University, State College, USA.

**Background/Rationale or Objectives/Purpose**

Young adults affected by cancer experience distinct developmental and psychosocial challenges yet have limited access to age-appropriate supportive care. Although yoga can enhance wellbeing for older adults affected by cancer, it has rarely been studied among young adults, and no sustainable, accessible, age-appropriate programs exist in Canada. Researchers and InspireHealth (a community-based cancer support organization) partnered and worked alongside people with lived experience and healthcare providers to co-create Young Adult Yoga (YAY), a 12-week community-based yoga program. YAY was piloted June 2025–August 2025 to explore feasibility and gather participant feedback and was formally launched October 2025. This abstract describes the co-creation process.

**Methodology or Methods**

Co-creation followed community-based participatory research principles. Partners engaged in iterative design cycles involving structured 1:1 consultations, group meetings, and reviewing draft materials. Decisions were guided by consensus and community partner capacity and documented using meeting notes. Pilot findings informed refinements to recruitment and registration pathways, delivery logistics, and evaluation.

**Impact on Practice or Results**

Partnership outputs currently include the following aspects: (i) shared decision-making structures; (ii) partner driven program modifications, (iii) implementation resources (instructor/moderator training); (iv) embedded collaborative recruitment, registration, and data collection (evaluation) pathways; and (v) a relevant evaluation battery. Additionally, partners co-designed a novel hybrid YAY retreat to be offered at the end of each 12-week program, extending opportunities for connection and supportive care.

**Discussion or Conclusions**

Co-creation has generated tangible outputs, strengthened community capacity, and established a foundation for sustained YAY delivery and evaluation. This collaboration will also generate important insights on yoga’s impact both acutely and overtime for young adults affected by cancer.

#### 2.32.8. 120: Co-Designing an Adolescent and Young Adult Cancer Care and Support Program in British Columbia

Cheryl Heykoop, Tiffany Hill and Dornian Kat.
Anew Research Collaborative, Victoria, Canada.

**Background/Rationale or Objectives/Purpose**

In British Columbia (BC), over 1200 adolescents and young adults (AYAs) are diagnosed with cancer each year. AYAs have distinct cancer care and support experiences and needs; however, in BC there is currently no AYA program. Further, evidence suggests AYAs want to play an active role in informing and shaping cancer care and support programming; however, they often have few opportunities to do so.

**Methodology or Methods**

Over the last four years, we have applied a patient-oriented, participatory action research approach to meaningfully engage over 150 AYAs and their cancer care allies (health care providers, leadership, researchers, and supporters) to identify and co-design an AYA program in BC that reflects the distinct needs and priorities of AYAs.

**Impact on Practice or Results**

In this workshop we will (1) share our novel and innovative approach to patient engagement to co-design BC’s first AYA program, (2) explore through experiential approaches how we use creative methods to support the meaningful engagement of AYAs and cancer care allies, and (3) discuss how this approach has resulted in the creation of an AYA program road map, key priorities and pilot initiatives to improve AYA cancer care and support.

**Discussion or Conclusions**

Our approach has galvanized momentum to establish an AYA cancer care and support program in BC. We will showcase how our approach to patient engagement and co-design has built momentum to establish an AYA program and the guiding principles that have informed, shaped, and sustained our work including a focus on partnerships, equity, and a holistic approach to AYA cancer care and support.

#### 2.32.9. 122: Imagine: Reshaping Adolescent and Young Adult Cancer Care and Support Through Immersive Performance

Cheryl Heykoop ^1^, Tiffany Hill ^1^, Kat Dornian ^1^ and Alice O’Grady ^2^
^1^ Anew Research Collaborative, Victoria, Canada;
^2^ University of Leeds, Leeds, United Kingdom.

**Background/Rationale or Objectives/Purpose**

Adolescents and young adults (AYAs) diagnosed with cancer between the ages of 15 and 39 have distinct cancer care and support experiences and needs that are different than adult and pediatric patients, and these needs and experiences are often unmet by cancer care and support systems. The aim of this project was to work with a group of AYAs with lived experiences with cancer to co-create an immersive performance—a performance that involves the audience as active participants.

**Methodology or Methods**

Over a 12-month period, we applied an arts-based patient-oriented, participatory action research methodology to work with 12 AYA co-researchers to co-design an immersive performance that captured the felt sense of what it means to be an AYA navigating cancer care and support in British Columbia.

**Impact on Practice or Results**

In November 2024, twelve 40-min immersive performances were shared with over 100 clinicians, care givers, decision makers, and AYAs at the BC Cancer Summit and we created two videos to showcase the experience. Initial reactions suggest the immersive performance was a powerful and impactful experience.

**Discussion or Conclusions**

This immersive performance was a creative and impactful research and knowledge translation tool to build knowledge and understanding and inspire change in AYA cancer care and support. This workshop will present the immersive experience (in physical or video format), will discuss the process of development (including the process, challenges, and opportunities), and explore implications for future practice.

#### 2.32.10. 125: Co-Designing Community-Based Breast Cancer Screening Pathways with Patient Partners: Lessons from the West Toronto Ontario Health Team

Anuoluwapo Awotunde ^1^, Kashtin Fitzsimons ^1^, Sathuja Braganza ^1^, Rumaisa Khan ^1^, Stephanie Haller ^1^, Alejandra Priego ^1,2^, Renata Ozimek ^1^ and Snighdha Khandker ^1^
^1^ West Toronto Ontario Health Team (WTOHT), Toronto, Canada;
^2^ Unity Health, Toronto, Canada.

**Background/Rationale or Objectives/Purpose**

Breast cancer screening disparities persist among equity-deserving populations in West Toronto, including newcomers, uninsured individuals, and people without a primary care provider. To address these gaps, the West Toronto Ontario Health Team (WTOHT) implemented a patient-partnered, community-driven awareness and screening model to reduce fear, strengthen early detection pathways, and expand access for systemically marginalized groups.

**Methodology or Methods**

Multiple co-designed screening events were delivered in collaboration with primary care practices, community organizations, preventive care providers, and patient partners. Activities included needs assessment, culturally attuned outreach, provider- and ambassador-led recruitment, tailored communication, transportation supports, guided tours of screening facilities, and real-time navigation for uninsured and unattached participants. Quantitative measures captured attendance, registration, insurance status, and screening completion, while qualitative feedback explored reported satisfaction, anxiety reduction, trust building, and overall experience.

**Impact on Practice or Results**

Across all events, more than seventy community members participated, with approximately thirty completing same-day mammograms. Uninsured and unattached participants were successfully linked to community diagnostic pathways. Participant feedback consistently highlighted reduced anxiety, improved clarity of the screening process, familiarity with the equipment and process, as well as a welcoming, streamlined experience. Patient partners played a central role in fostering cultural safety, engagement, and trust.

**Discussion or Conclusions**

This patient-partnered, community-based model demonstrates strong potential for improving equitable access to breast cancer screening. Lessons learned will inform upcoming expansions, including outdoor events, strengthened navigation, and deeper primary care involvement across Ontario Health Teams to continue to address hard to reach population in fostering preventative care.

**Questions for Audience Feedback**

1. What additional supports could further reduce psychosocial barriers for first-time screeners?
2. How can follow-up and navigation be strengthened for non-insured or unattached patients?
3. What metrics best capture psychosocial impact in community-based prevention initiatives?

#### 2.32.11. 135: Better Research Through Collaboration

Anu Shukla-Jones, Mackenzie Huckvale, Jayden Domitsu, Megan Mahoney and Stephanie Michaud.
BioCanRx—Canada’s Immunotherapy Network, Ottawa, Canada.

**Background/Rationale or Objectives/Purpose**

To understand how patient partners have contributed to BioCanRx-funded research in preclinical and early phase clinical trials (translational research). We want to understand what patient partnerships in translational research looks like.

**Methodology or Methods**

BioCanRx reviewed all funded projects (applications, interim reports and final reports) since 2015. We looked for all information related to patients as partners in research, including the following: number of projects that included patients, how patients were designed into the project, and how patients contributed to projects based on descriptions provided in the reports.

**Impact on Practice or Results**

Patient partners in research increased from 7% of projects in Cycle 1 (2015–2019) to 73% in Cycle 2 (2020–2024), and currently 95% of research funded by BioCanRx includes patients as partners in research. The Learning Institute started in 2017 and has supported patient partnership in research ever since.

**Discussion or Conclusions**

We learned that patient partners improve the knowledge of researchers and research teams and the quality of research. Patients are most valuable for research focused on the following areas:

■ Short-term, early-stage, proof-of-concept studies for biotherapeutic development, assessment and validation.
■ Bridging laboratory and clinical testing, such as toxicology.
■ Phase I/II clinical trials for new immunotherapies.
■ Solutioning to address social, legal, ethical, economic, and health systems barriers for cancer immunotherapies.
■ Projects focused on refining biomanufacturing processes do not yet have a clear role for patient partners.
■ The Learning Institute provides bidirectional learning opportunities to support meaningful patient partnerships in research that has improved BioCanRx funded research over time.

#### 2.32.12. 150: Patient-Informed Development of a Sexual Health Animation for Women Undergoing Pelvic Radiation

Dalia Peres ^1^, Lauren Jones ^2^, Jodie Jenkinson ^2^, Michelle Jacobson ^3^, Taylor Incze ^1^, Steven Guirguis ^1^, Taylor Roebotham ^1^ and Andrew Matthew ^1,4^
^1^ Department of Surgical Oncology, Princess Margaret Cancer Centre, University Health Network, Toronto, Canada;
^2^ University of Toronto, Toronto, Canada;
^3^ Department of Gynecology, Women’s College Research Institute, Toronto, Canada;
^4^ Department of Supportive Care, Princess Margaret Cancer Centre, University Health Network, Toronto, Canada.

**Background/Rationale or Objectives/Purpose**

Cancer treatments frequently compromise sexual health (SH) and quality of life; however, sexual healthcare—particularly for women—remains a significant gap in oncology care. To address this need, the Sexual Health Clinic (SHC) at Princess Margaret Cancer Centre was established as a hybrid in-person and virtual model delivering comprehensive biopsychosocial care for cancer-related sexual dysfunction. As the clinic’s female-focused programming continues to evolve, and in recognition of the value of videos and animations in patient education, a novel, patient-informed animation was developed to enhance pelvic radiation patients’ sexual health knowledge and support equitable, compassionate survivorship care.

**Methodology or Methods**

Semi-structured interviews were conducted with 12 female pelvic radiation patients to explore experiences, symptoms and animation preferences. Interviews were audio-recorded, transcribed, and analyzed thematically to inform the educational content, tone, and biopsychosocial framing of the animation.

**Impact on Practice or Results**

The animation supports patient-centred, survivorship-focused care by delivering empirically informed education on the sexual side effects of pelvic radiation therapy and available treatment options. Designed to be accessible and emotionally supportive, it provides a low-barrier entry point to learning, expands access to reliable and equitable sexual health information, reduces reliance on provider-led discussions, and empowers women to engage in informed, meaningful conversations about their care.

**Discussion or Conclusions**

The animation highlights the value of thoughtful patient-centred care in reducing stigma, normalizing SH, and validating patients’ experiences following cancer treatment. The biopsychosocial foundation embedded in the animation may help reduce patients’ distress and improve SH outcomes. Future work should consider extending these principles to other treatment types and patient populations.

How can animations be most effectively integrated into oncology and survivorship workflows to normalize sexual health discussions without increasing clinician burden?

What psychosocial elements (e.g., validation, normalization, relational context) are most critical to include in visual sexual health education for women following pelvic radiation?

How might this type of low-barrier, virtual resource help reduce inequities in access to sexual health care across diverse oncology populations?

### 2.33. Final Category: M. Primary, Secondary and Tertiary Cancer Prevention

#### 2.33.1. 5: Breast Cancer in Young Women: Breaking Knowledge Barriers to Early Detection and Improving Outcomes Through Youth Council for Breast Health Chapters on Campuses Across Canada

Lorna Larsen ^1^ and Anna Feehan. ^2^
^1^ Team Shan Breast Cancer Awareness for Young Women, Huntsville, Canada;
^2^ Memorial University, St. John’s, Canada.

**Background/Rationale or Objectives/Purpose**

The incidence of breast cancer in young women (BCYW) continues to increase at alarming rates. With no routine screening available, young women are frequently diagnosed late with advanced disease and poorer outcomes. Limited awareness of breast cancer risk, symptoms and self care strategies negatively impacts help-seeking behaviour. Health-promotion strategies targeting young women to reduce knowledge barriers have been effective in increasing early detection, decreasing distress, and improving outcomes.

**Methodology or Methods**

The international BCYW Foundation launched the Youth Council for Breast Health (YCBH) program to protect the lives of young women on campuses by raising awareness about breast health, breast cancer symptoms and risk factors, promoting prevention and early detection. Team Shan Breast Cancer Awareness for Young Women (Team Shan), a Canadian charity has partnered with the BCYWF to facilitate YCBH chapters on campuses in Canada. Through public health best practice, Team Shan has successfully developed breast cancer awareness campaigns and resources to reach young women on college and university campuses with their breast cancer risk and breast health information.

**Impact on Practice or Results**

YCBH activities have been piloted with positive results e.g., pop-up campus library displays and print material sharing on campus. Four Canadian university campuses have registered for the YCBH program since the spring of 2025 and others are in the process. Activities have included campus displays, presentations, and Team Shan resource dissemination with positive results. Students have been interested in the topic, appreciated the awareness and some have volunteered to assist their YCBH chapter on campus. Data collection is ongoing. One year of data from Memorial University will be shared in the presentation.

**Discussion or Conclusions**

Peer-to-peer initiatives have been successful as health-promotion strategies. Team Shan’s use of Shan’s face and story as well as targeted messaging has resonated with young women on campus, and empowered them to take action. Goals for earlier detection and improved outcomes have been achieved. It is hoped that the YCBH program will continue to grow on post-secondary schools across Canada.

#### 2.33.2. 9: Religion and Public Health Communication: HPV Vaccine News in Canada

Xiaohe Sun ^1^, Tyler Sun ^2^, Sharlane C. L. Lau ^3^, Allison McArthur ^4^, Ishani Nath ^5^ and Gilla K. Shapiro ^3,5,6^
^1^ Temerty Faculty of Medicine, University of Toronto, Toronto, Canada;
^2^ Faculty of Health Sciences, McMaster University, Hamilton, Canada;
^3^ Department of Supportive Care, Princess Margaret Cancer Centre, Toronto, Canada;
^4^ Department of Library Services, Public Health Ontario, Toronto, Canada;
^5^ Centre for Vaccine Preventable Diseases, Dalla Lana School of Public Health, University of Toronto, Toronto, Canada;
^6^ Social and Behavioural Health Sciences Division, Dalla Lana School of Public Health, University of Toronto, Toronto, Canada.

**Background/Rationale or Objectives/Purpose**

Human papillomavirus (HPV) can cause several cancers. In Canada, HPV vaccination is publicly funded, yet uptake has not consistently reached coverage targets. Media reporting on the perspectives of religious leaders may impact vaccine behavior; however, there has been limited exploration of media depictions of religious perspectives of HPV vaccination in Canada. This study examined the content and tone of news media’s depiction of religion and HPV vaccination in Canada.

**Methodology or Methods**

To identify news reports for our content analysis, we searched Canadian Newsstream, Global Newsstream, and Factiva for English language news articles between 2006 and 2024. Published articles covered a 13-year period (2006–2018); no articles were found between 2019 and 2024. We coded 333 articles for the following aspects: (1) publication information, (2) article tone, (3) description of religious leaders’ or members’ views toward HPV vaccination, (4) voices represented, and (5) HPV vaccination content reported.

**Impact on Practice or Results**

Most articles adopted a neutral (72%) or positive (23%) tone toward HPV vaccination. Most articles (97%) focused on Catholic perspectives, with minimal inclusion of other faiths. The most frequently quoted voices in the articles were from school board trustees or administrators (46%), religious leaders (45%), and healthcare professionals (43%). In the articles, medical professionals (68%) and community leaders (45%) were frequently cited as endorsing the HPV vaccine, while religious leaders were less often cited as endorsing vaccination (14%).

**Discussion or Conclusions**

Support for the vaccine by religious leaders or members primarily focused on disease prevention, while opposition often centered on concerns about promoting sexual activity and misalignment with religious values. As HPV vaccination policies evolve, including endorsement from interfaith leaders and other stakeholders may be important for inclusive health journalism, accurate portrayal of wide-ranging religious perspectives, and the public health priority to increase HPV vaccination.

#### 2.33.3. 79: Evaluating the Behavioural and Social Drivers of HPV Vaccination in Canada: A Study Protocol for a Longitudinal Mixed Methods Study

Gilla Shapiro ^1,2^, Ève Dubé ^3^, Kerrie Wiley ^4^, Bärbel Knäuper ^5^, Shelly Bolotin ^2^, Justin Presseau ^6^, Tara Martin ^2^, Madison Leggatt ^1^, Nivatha Moothathamby ^1^ and Noel Brewer ^7^
^1^ Princess Margaret Cancer Centre, Toronto, Canada;
^2^ University of Toronto, Toronto, Canada;
^3^ Laval University, Quebec City, Canada;
^4^ University of Sydney, Sydney, Australia;
^5^ McGill University, Montreal, Canada;
^6^ Ottawa Hospital Research Centre, Ottawa, Canada;
^7^ University of North Carolina, Chapel Hill, USA.

**Background/Rationale or Objectives/Purpose**

The human papillomavirus (HPV) vaccine prevents cervical, head and neck, and anogenital cancers, but its uptake is below the Canadian national target of 90%. The World Health Organization and partners developed a survey and interview guide to understand the behavioural and social drivers (BeSD) of HPV vaccination, but these tools have not yet been evaluated. This project will (1) examine the psychometric properties of the BeSD HPV survey; (2) field test the BeSD HPV interview guide; (3) identify factors that contribute to low HPV vaccine uptake; and (4) identify BeSD HPV factors that explain variation in vaccine uptake over time.

**Methodology or Methods**

A longitudinal embedded mixed methods study design will be used to evaluate the BeSD HPV survey. A sample of 4000 parents or caregivers of children eligible for the HPV vaccine (ages 9–14) will be recruited from across Canada. A subset of 50 survey respondents will be recruited for a qualitative interview. The survey and interview will be repeated one year later with the same participants to understand changes in vaccination behaviours.

**Impact on Practice or Results**

The results generated from this study will inform actionable ways to increase HPV vaccine uptake in Canada.

**Discussion or Conclusions**

The validated BeSD HPV tools will enable program managers and researchers to systematically gather data to inform the design of HPV vaccination programs in Canada. Research findings will thereby support the goal to increase HPV vaccine uptake and prevent HPV-associated cancers.

#### 2.33.4. 81: Using Cognitive Interviewing to Test the Interpretability, Comprehensibility, and Relevance of the Behavioural and Social Drivers of Human Papillomavirus Vaccination Survey in Canadian Parents

Tara Martin ^1^, Kerrie Wiley ^2^, Nivatha Moothathamby ^3^, Madison Leggatt ^3^, Ève Dubé ^4^, Shelly Bolotin ^1^, Lisa Menning ^5^, Paul Bloem ^5^, Noel Brewer Brewer ^6^ and Gilla Shapiro ^1,3^
^1^ University of Toronto, Toronto, Canada;
^2^ University of Sydney, Sydney, Australia;
^3^ Princess Margaret Cancer Centre, Toronto, Canada;
^4^ Laval University, Quebec City, Canada;
^5^ World Health Organization, Geneva, Switzerland;
^6^ University of North Carolina, Chapel Hill, USA.

**Background/Rationale or Objectives/Purpose**

Human papillomavirus (HPV) vaccines are highly effective in preventing infection and pre-cancerous lesions when administered prophylactically and therefore are offered to school-aged children. Despite this, HPV vaccine coverage is below national targets. To better understand factors associated with HPV vaccine uptake, the World Health Organization (WHO) and partners developed the Behavioural and Social Drivers (BeSD) of HPV vaccination survey. This study tested the interpretability, comprehensibility, and relevance of the BeSD HPV survey in the Canadian context.

**Methodology or Methods**

Canadian parents/caregivers of children aged 9–14 completed cognitive interviews on the BeSD HPV survey in English or French. Open-ended, qualitative interviewing techniques were used. Survey items found to be confusing or misunderstood were identified and the survey was revised in an iterative process in consultation with the WHO’s tool developers.

**Impact on Practice or Results**

In total, 15 interviews were completed (13% in French) from January to June 2025. Most participants were women (87%), white (67%), and reported their child was not HPV-vaccinated (80%). Participants identified language, temporal, logical, and validity challenges. Interpretability challenges were identified for survey items not applicable to the child’s vaccine eligibility or school context. Comprehensibility was enhanced through wording and translation revisions. Items relating to vaccine cost or delivery in health facilities were removed to align with Canada’s publicly funded, school-based programs.

**Discussion or Conclusions**

Survey items were revised to improve comprehension and relevance to the Canadian setting. The revised survey will be psychometrically validated and factors that contribute to low HPV vaccine uptake will be examined.

### 2.34. Final Category: N. Novel Interventions and Clinical Trials in PSO

#### 2.34.1. 58: A Progress Report on the Pilot Study of an Adaptation of the Fear of Recurrence Therapy to Parents of Pediatric Cancer Survivors—Parent-FORT

Sophie Lebel ^1^, Celeste Holy ^1^, Janelle Gibson-Haerle ^2^, Jeff Brinson ^3^, Lesleigh Abbott ^4^, Lauriane Giguère ^1^, Amy Givogue ^4^, Greg Guilcher ^5^, Lauren Heathcote ^6^, Emily Johnson ^4^, Christine Maheu ^7^, Kim McMillan ^1^, Wendy Pelletier ^8^, K. Brooke Russell ^1^, Fiona Schulte ^8^, Heloise Sirois-Leclerc ^4^ and Perri Tutelman ^8^
^1^ University of Ottawa, Ottawa, Canada;
^2^ Patient partner, Calgary, Canada;
^3^ Patient partner, Hamilton, Canada;
^4^ CHEO, Ottawa, Canada;
^5^ Alberta Children Hospital, Calgary, Canada;
^6^ King’s College London, London, United Kingdom;
^7^ McGill University, Montreal, Canada;
^8^ University of Calgary, Calgary, Canada.

**Background/Rationale or Objectives/Purpose**

Objectives: Fear of cancer recurrence (FCR) can be addressed in cancer survivors with brief interventions, such as the FORT group intervention. However, none of these interventions have been tested with parents of survivors of childhood cancer. The goal of the present study is to test the acceptability, feasibility, and effect size of a six-week FORT intervention for parents (Parent-FORT) in a multimethod randomized pilot study.

**Methodology or Methods**

Sample and setting: The inclusion criteria are as follows: (1) parent (or primary caregiver) of a survivor of childhood cancer; (2) presenting with clinical levels of FCR; and (3) access to a computer with an internet connection. Participants will be randomized to the intervention or to a waitlist control group, in which case they will be offered the intervention after the study. Methods: Parents will be recruited directly via clinicians, databases of survivors of childhood cancer, and community support partners. The feasibility criteria are as follows: (1) ability to recruit and randomize 36 parents in 12 months; (2) ability to deliver FORT-Parent to 29 parents in 15 months; (3) 80% of participants completing 5 out of 6 sessions; (4) complete measures for at least 90% of participants; (5) ability to deliver FORT-Parent as intended; and (6) satisfactory ratings > than 80%.

**Impact on Practice or Results**

As of November 2025, we have screened and recruited 20 and 12 parents in 3 months, respectively. A first group will be offered in January 2026.

**Discussion or Conclusions**

Clinical implications: Our proposed research has direct implications for clinical services development to improve quality of life for parents of childhood cancer survivors.

#### 2.34.2. 59: Acceptability and Preliminary Effectiveness of a Brief Narrative Care Intervention for Patients at the Conclusion of Primary Cancer Treatment

Lucas Norton ^1^, Iana Ianakieva ^1^, Benjamin Stevenson ^1^, Darby Erler ^2^, Catriona Buick ^1,2^, Erin Gross ^2^ and Karen Fergus ^1,2^
^1^ York University, Toronto, Canada;
^2^ Sunnybrook Health Sciences Centre, Toronto, Canada.

**Background/Rationale or Objectives/Purpose**

The transitional phase following the completion of treatment for primary cancer is commonly associated with elevated distress and existential concerns. It can also be the impetus for deeper reflection and meaning making in relation to one’s personal cancer journey. A narrative care approach to supporting patients at this critical juncture holds promise given evidence that such approaches can foster expression of personal agency, enhanced perspective, and experientially derived insights. This study examined the acceptability and preliminary effectiveness of a 90-min single session narrative care intervention (NCI) for individuals at the conclusion of primary cancer treatment.

**Methodology or Methods**

Data for this study were collected from a heterogeneous sample of cancer patients recruited between 2017 and 2024 at Sunnybrook’s Odette Cancer Centre. Participants (n = 42) were asked to complete pre- and post-NCI questionnaires (which included measures of affect, wellbeing, and self-efficacy in coping with cancer) in addition to participating in the NCI—a 90-min semi-structured interview which guides patients through a telling of their cancer story from diagnosis to the conclusion of treatment. Limited effectiveness of the study was examined using a within-subjects design; a two-tailed paired-sample *t*-test was used to detect significant differences between self-report measures pre- and post-NCI. 32 participants completed the post-NCI questionnaire which also included a treatment satisfaction survey comprised of binary questions (yes/no), Likert scale questions, and open-ended text box responses. These results were examined quantitatively and qualitatively to ascertain acceptability and feasibility of the NCI.

**Impact on Practice or Results**

Limited effectiveness testing revealed a significant increase in participant reported experiences of social wellbeing. Examination of treatment satisfaction indicated that 93% of participants felt that the NCI was helpful, and 73% agreed or strongly agreed that “the interview highlighted important insights/learnings in relation to my experience with cancer”. Qualitative themes identified included a deeper recognition of one’s own struggles, a more nuanced understanding of the varied impacts cancer has had on a range of life domains, and changes in perspective around relationships with loved ones (such as recognition of the value of caregiver support and greater clarity around setting boundaries). Taken together, these findings support the acceptability, and potential benefit of a single session, narrative care intervention for patients following the completion of treatment.

**Discussion or Conclusions**

As the NCI is a brief, time-limited intervention, it demands fewer psychosocial resources than a full course of therapy and provides greater accessibility for individuals who could benefit from narrative support but may not require longer-term therapeutic intervention. It could further serve as a screening tool to identify individuals who are appropriate for referral to more comprehensive psychosocial support.

Questions: Could a single-session narrative care intervention be incorporated into your hospital/clinic? Do you see such an intervention at the conclusion of treatment as filling a psychosocial support need? Do you have thoughts on ways in which patients could be identified as appropriate candidates for an intervention like the NCI?

#### 2.34.3. 102: Connected Care: Rapid Virtual Interdisciplinary Psychiatry for Cancer Patients

Deborah McLeod ^1^, Christian Schulz-Quach ^2,3,4^, Catherine Chan ^5^, Ying Jiang ^5^, Laura Beatty ^1^, Katie Hucklebridge ^1^, Colleen Dunphy ^5^, Alexandre Ngoc Nguyen Teichmann ^3,6^ and Meaghan Wright ^5^
^1^ Carepath Digital Health, Mississauga, Canada;
^2^ Department of Psychiatry, University of Toronto, Toronto, Canada;
^3^ Institute of Health Policy, Management and Evaluation, University of Toronto, Toronto, Canada;
^4^ University Health Network—Princess Margaret Cancer Centre, Toronto, Canada;
^5^ Ontario Health (Cancer Care Ontario), Toronto, Canada;
^6^ Department of Psychiatry, Faculty of Medicine, University of Ottawa, Toronto, Canada.

**Background/Rationale or Objectives/Purpose**

People with cancer in rural and remote communities face substantial barriers to accessing specialized psychiatric care, contributing to poorer cancer-related outcomes and increased morbidity. To address these gaps, Ontario Health, Carepath Digital Health, and CPAC launched the Virtual Psychiatry Assessment (VISTA) Program in December 2024, expanding access to specialized mental health support through a rapid, collaborative virtual care model.

**Methodology or Methods**

Over two appointments, VISTA provides the following: (1) comprehensive psychosocial evaluation conducted by a mental health clinician (MHC), (2) joint consultation involving the MHC and a consulting psychiatrist. Detailed psychiatric management plans were developed collaboratively for the referring providers. Seventeen patients were referred to the pilot, and fourteen of them completed the full assessment pathway. Patient satisfaction was high (100% rated their experience as very good).

The mixed-methods evaluation assessed the feasibility, acceptability, and implementation of VISTA across two Regional Cancer Centres in Northern Ontario. Quantitative measures included referral volumes, PHQ-9/GAD-7 scores, and process timelines; qualitative data were obtained from patient surveys and semi-structured interviews.

**Impact on Practice or Results**

Virtual psychiatric consultation models show promise for extending and accelerating specialized mental health care to rural cancer patients; success relies on robust system integration, clear referral workflows and coordination across providers.

**Discussion or Conclusions**

Key implementation challenges included hesitation among referring providers to assume management plan responsibility, administrative barriers with billing requirements, and system-level communication gaps between oncology, mental health and primary care. Success factors included team-based care coordination, administrative support, and technological flexibility. Sustained implementation will require improved referral pathways, provider education, and system-level coordination between oncology and primary care.

#### 2.34.4. 121: A Mixed-Methods Pilot Study Evaluating the Therapeutic Impact of a Digital Storytelling Group for Women with Premenopausal Breast Cancer

Janet de Groot ^1,2^, Jessame Gamboa ^3^, Dana Male ^2^, Fay Strohschein ^3^, Laura Labelle ^2^, Hao Chloe ^1^ and Kathleen Sitter ^3^
^1^ Cumming School of Medicine, Calgary, Canada;
^2^ Arthur JE Child Comprehensive Cancer Centre, Calgary, Canada;
^3^ University of Calgary, Calgary, Canada.

**Background/Rationale or Objectives/Purpose**

To explore the therapeutic benefits of a pilot novel arts-based intervention (six-session digital storytelling group [DST-G]) for women with premenopausal breast cancer.

**Methodology or Methods**

We conducted a concurrent mixed-methods study to assess (a) Patient-Reported Outcomes on depressive and post-traumatic stress symptoms and quality of life on completion of DST-G; (b) explore experiences, perceived therapeutic benefits and potential mechanisms of action of DST-G utilizing a semi-structured interview.

**Impact on Practice or Results**

Participants showed a moderate reduction in depressive symptoms (PHQ9). For post-traumatic stress symptoms (PCL-5), participants showed an average reduction of 9.7 points (SD = 17.8), which indicates a Minimal Clinically Important Difference (MCID). Quality-of-life scores showed a small mean improvement.

Participants experienced connection and belonging within the group, which supported storytelling. During the creative process, they found reliving treatment and changes in relationships an at times, daunting process. In confronting difficult questions and making narrative, visual and musical choices to develop a digital story, the participants described feeling transformed and more at peace.

**Discussion or Conclusions**

The qualitative findings provide insight into the therapeutic potential of digital stories. Additionally, the quantitative findings indicate the need for an adequately powered sample size to evaluate effectiveness of the DST-G arts-based intervention.

1. What questionnaires are recommended to evaluate patient reported outcomes?
2. What interview questions could be added to further explore the potential mechanisms of action of DST-G.

#### 2.34.5. 143: Association of Insomnia with Dose Reduction and Discontinuation of Adjuvant Palbociclib in Early Breast Cancer: The PALLAS Trial (ABCSG-42, AFT-05)

Claudia McBrearty ^1,2,3^, Dominik Hlauschek ^4^, Erica Mayer ^5^, Josée Savard ^1,2,3^, Florence Lévesque ^3^, Julie Lemieux ^1,3^, Manuel Ruiz-Borrego ^6^, Tiffany Traina ^7^, Katherine Clifton ^8^, Daniel Egle ^4,9^, Diana Lake ^7^, Silvia Antolin-Novoa ^10^, Judith Bliss ^11^, Hope Rugo ^12^, Gabriele Zoppoli ^13^, Philipp Wittmann ^14^, Lidija Sölkner ^14^, Michael Gnant ^15^ and Angela DeMichele ^16^
^1^ Cancer Research Centre, Faculty of Medicine, Université Laval, Quebec, Canada;
^2^ School of Psychology, Université Laval, Québec, Quebec, Canada;
^3^ CHU de Québec, Université Laval, Quebec, Canada;
^4^ Austrian Breast and Colorectal Cancer Study Group, Vienna, Austria;
^5^ Dana-Farber Cancer Institute, Boston, USA;
^6^ Hospital Virgen del Rocío Sevilla, GEICAM Spanish Breast Cancer Group, Sevilla, Spain;
^7^ Memorial Sloan Kettering Cancer Center, New York, USA;
^8^ Department of Medicine, Division of Oncology, Washington University, Saint Louis, USA;
^9^ Department of Gynecology and Gynecological Oncology, Medical University of Innsbruck, Innsbruck, Austria;
^10^ Complejo Hospitalario Universitario A Coruña, A Coruña, Spain;
^11^ The Institute of Cancer Research, London, United Kingdom;
^12^ University of California San Francisco, San Francisco, USA;
^13^ IRCCS Ospedale San Martino, Genoa, Italy;
^14^ Austrian Breast and Colorectal Cancer Study Group (ABCSG), Vienna, Austria;
^15^ Medical University of Vienna, Vienna, Austria;
^16^ Perelman School of Medicine, University of Pennsylvania, Philadelphia, USA.

**Background/Rationale or Objectives/Purpose**

This study aimed to evaluate the association between sleep problems and palbociclib dose reduction and premature discontinuation in patients receiving adjuvant palbociclib for breast cancer.

**Methodology or Methods**

This post hoc analysis was conducted using data from the PALLAS trial (NCT02513394), a prospective, multicenter, randomized phase III study that enrolled 5796 patients with hormone receptor-positive, HER2-negative early breast cancer. The present analysis included patients randomized to the palbociclib plus adjuvant endocrine therapy arm (n = 2880), of whom 2166 completed the cycle 1 day 1 (baseline) and at least one follow-up EORTC QLQ-C30 assessment. Patients were classified as having insomnia or as good sleepers based on the EORTC QLQ-C30 sleep item (range: 1–4), with a score of 3 used as the threshold for insomnia. Simple and time-dependent Fine and Gray regression was used to assess the association of insomnia with palbociclib adherence.

**Impact on Practice or Results**

Patients with baseline insomnia showed a numerically higher cumulative incidence of early palbociclib discontinuation in unadjusted analyses (*p* = 0.051). In univariable models, baseline insomnia was not significantly associated with early discontinuation (Hazard Ratio [HR] = 1.15; 95% Confidence Interval [CI]: 1.00–1.34; *p* = 0.056). In contrast, insomnia at any timepoint was associated with a higher risk of early discontinuation (HR = 1.15, 95% CI: 1.01–1.31; *p* = 0.038). After adjustment for clinical, demographic, and quality-of-life covariates, neither baseline nor time-dependent insomnia remained significantly associated with early discontinuation.

**Discussion or Conclusions**

The findings suggest that sleep problems do not predict palbociclib adherence over and above the influence of the overall symptom burden.

Support: AFT, ABCSG, Pfizer, PrECOG, NSABP Foundation, GBG, BIG: 

https://acknowledgments.alliancefound.org (accessed on 1 January 2026).

#### 2.34.6. 149: The Evolution of a Sexual Health Clinic in an Urban Oncology Centre: Real-World Uptake and Biopsychosocial Outcomes

Andrew Matthew ^1,2^, Steven Guirguis ^1^, Dalia Peres ^1^, Taylor Incze ^1^, Bibiana Kemerer ^3^, Nicolle Fox ^3^, Taylor Roebotham ^4^ and Dean Elterman ^5^
^1^ Department of Surgical Oncology, Princess Margaret Cancer Centre, University Health Network, Toronto, Canada;
^2^ Department of Supportive Care, Princess Margaret Cancer Centre, University Health Network, Toronto, Canada;
^3^ Princess Margaret Cancer Centre, University Health Network, Toronto, Canada;
^4^ Department of Obstetrics and Gynecology, Princess Margaret Cancer Centre, University Health Network, Toronto, Canada;
^5^ Division of Urology, University Health Network, Toronto, Canada.

**Background/Rationale or Objectives/Purpose**

Sexual health is commonly compromised following cancer treatment, with long-term sexual dysfunction (SD) affecting up to 90% of cancer survivors in Canada. Unfortunately, access to specialized sexual health care is limited, as traditional in-person models are constrained by geographic and funding barriers. Digital health innovations offer a scalable solution. This abstract reports early uptake, engagement, and outcomes of a hybrid in-person and virtual Sexual Health Clinic (SHC) implemented within a large urban cancer centre.

**Methodology or Methods**

The SHC uses a biopsychosocial, interdisciplinary model combining virtual education modules, virtual care facilitation by a sexual health counsellor, and in-person clinic visits. Real-world program data were analyzed to assess SHC uptake over its first three years (2023–2025). Virtual platform engagement and Patient-Reported Outcomes were examined for male patients only, reflecting phased implementation, with female-focused services under development.

**Impact on Practice or Results**

The SHC received over 400 referrals annually, representing over 30 cancer types. Over 88% indicated an intention to attend, with 80% completing initial appointments. Virtual engagement was robust, with a mean of 207 module pages viewed, over 2000 trackers completed, and more than 2300 messages exchanged with sexual health counsellors. Baseline assessments identified SD including erectile dysfunction (72%), orgasmic dysfunction (48%), and reduced sexual desire (39%), with improvements observed over time among patients engaged in care.

**Discussion or Conclusions**

Findings support a hybrid virtual and in-person model of sexual health care in oncology. Future work will evaluate virtual services for female sexual dysfunction and longer-term patient outcomes.

Questions:

How can hybrid sexual health models be integrated into cancer care pathways without increasing provider burden?

As services expand to female survivors, what unique barriers and facilitators should be anticipated compared with delivering care to male survivors?

What adaptations to this hybrid model would be required to improve access across diverse geographic and sociocultural contexts?

#### 2.34.7. 151: Centering Families in Cancer Care: Attachment-Informed Mindfulness Framework

Sofia Barkova and Linda E. Carlson.
University of Calgary, Calgary, Canada.

**Background/Rationale or Objectives/Purpose**

As the burden of cancer is growing, many families are experiencing its impact. People living with cancer, their caregivers, and other family members are all at high risk for distress. Centering families in psychosocial oncology within an attachment framework, trained clinicians can assess and help alleviate the distress of all family members. Family-centered care may also be more culturally appropriate than individually targeted interventions for the many communally focused cultures represented in Canada. There is a paucity of research on family-focused psychosocial interventions in adult cancer care generally, and even more so from an attachment perspective focusing on communal cultures.

**Methodology or Methods**

To fill this gap in family-focused care, we suggest applying a framework shaped by the dynamic maturational model of attachment and adaptation (DMM). The DMM model interprets interpersonal patterns as manifestations of self-protective attachment strategies that also affect individuals’ information-processing styles. Mindfulness-based cancer recovery (MBCR) is a structured and evidence-based training program in mindfulness skills which has proven effective for distress management in people with cancer and, occasionally, dyads. To address family distress on a larger scale, we propose a novel attachment-informed mindfulness-based multifamily intervention for families facing cancer.

**Impact on Practice or Results**

This approach leverages established evidence for the MBCR program while extending the dynamic maturational model of attachment and adaptation to oncology, potentially decreasing familial distress.

**Discussion or Conclusions**

Research is needed to explore the feasibility of the proposed conceptual approach. Moreover, experts in multicultural aspects of cancer care and patient partners from different cultural backgrounds should be involved in program development and testing before future implementation.

Questions for feedback: (1) Do you see attachment theory as potentially useful in PSO? What are potential barriers to its use by PSO professionals? (2) Can you envision adding attachment-related content to mindfulness-based interventions? What might that look like? (3) What are barriers to the feasibility of a family-centered intervention as described above? What are potential strengths? In which setting is it most likely to work well?

### 2.35. Final Category: O. Survivorship

#### 2.35.1. 17: Efficiency of a Stepped Care Cognitive–Behavioural Therapy for Insomnia Integrated in Routine Cancer Care: Results of the IMPACT Program

Josée Savard ^1,2,3^, Émilie Godin ^2,3^, Aude Caplette-Gingras ^2,4^, Charles M. Morin ^1,5^, Lynda Bélanger ^2^, Marie-Pierre Gagnon ^6^ and Marie-Ève Lamontagne ^7^
^1^ École de psychologie, Université Laval, Québec, Canada;
^2^ CHU de Québec-Université Laval Research Center, Québec, Canada;
^3^ Université Laval Cancer Research Center, Québec, Canada;
^4^ Centre des maladies du sein, CHU de Québec-Université Laval, Québec, Canada;
^5^ CERVO Brain Research Centre, Québec, Canada;
^6^ Faculté des sciences infirmières, Université Laval, Québec, Canada;
^7^ Département de réadaptation, Université Laval, Québec, Canada.

**Background/Rationale or Objectives/Purpose**

Insomnia is highly prevalent in cancer patients. Yet, it often remains overlooked and undertreated. Cognitive–behavioural therapy for insomnia (CBT-I) is the treatment of choice for cancer-related insomnia but its accessibility is limited. The main goals of the IMPACT (Insomnia in Patients with Cancer—Personalized Treatment) program were to assess the feasibility and efficiency of implementing a stepped care CBT-I in four cancer centers in the Quebec City area. This presentation will focus on efficiency data.

**Methodology or Methods**

Patients having a score ≥ 4 on the sleep item of the Edmonton System Assessment System-Revised (ESAS-R-sleep) received a leaflet explaining the stepped care CBT-I and how to access the web-based program (Step 1; called Insomnet). Patients with residual insomnia symptoms after completing Step 1 were offered 1–3 booster sessions with a clinical psychologist (Step 2). Patients completed a daily sleep diary throughout the course of the program and a battery of self-report questionnaires at pre- and post-Step 1 and at 6-, 12-, and 24-month follow-ups.

**Impact on Practice or Results**

The mean ISI score (N = 188) significantly decreased from 17.5 at pre-treatment to 8.8 at post-Step 1 (*p* < 0.0001; *d* = −2.3). The insomnia remission rate was 47.9%. These effects were well maintained at follow-up. During Step 1, significant improvements of all sleep parameters from a daily diary were found (all *p* < 0.0001), with effects of a moderate-to-large magnitude (N = 373). Step 2 further reduced wake time in unremitted patients following Step 1 (N = 13). Significant improvements of depression, anxiety, fatigue, quality of life and satisfaction with life were also found.

**Discussion or Conclusions**

Together, these findings indicate that a stepped care CBT-I integrated in routine cancer care is effective and produces improvements that are similar to those found in randomized controlled trials conducted with selected samples.

#### 2.35.2. 23: Insights into the Workplace Experiences of Individuals Affected by Cancer in Newfoundland and Labrador

Krista King ^1^, Derrick Bishop ^2^, Stephanie Budgell ^3^, Melanie Vokey ^1^, Georgia Skardasi ^1^, Cindy Whitten ^1,4^, Holly Etchegary ^1^, Eric Tenkorang ^1^, Teri Stuckless ^1,4^ and Sevtap Savas ^1^
^1^ Memorial University of Newfoundland, St. John’s, Canada;
^2^ Patient partner, CBS, Canada;
^3^ Patient partner, Newfoundland, Canada;
^4^ NL Health Services, St. John’s, Canada.

**Background/Rationale or Objectives/Purpose**

Newfoundland and Labrador (NL) have the second-highest incidence rate of cancer in Canada. However, limited research has examined how individuals affected by cancer navigate employment after diagnosis. We explored the workplace experiences of individuals affected by cancer in NL, with particular attention to the supports and challenges encountered throughout the cancer journey.

**Methodology or Methods**

We present findings from a subset of a larger study. A total of twenty-five participants (n = 25) were recruited, all of whom had received a cancer diagnosis within the preceding five years. Participants resided in urban centres as well as rural and remote communities. This diversity offered valuable insights across different geographic regions and employment contexts.

**Procedures:** Two patient partners were engaged throughout all phases of the research process. Qualitative data-collection methods and thematic analysis were used to explore participants lived workplace experiences. A survey was utilized to capture sociodemographic information.

**Impact on Practice or Results**

Some participants experienced empathetic and supportive environments in the workplace, while others encountered significant barriers such as (i) limited return-to-work flexibility, (ii) discrimination, (iii) inadequate health benefits, and (iv) workplace hostility. Self-employed individuals experienced additional challenges due to lack of benefits and limited capacity to maintain work during active treatment.

**Discussion or Conclusions**

These findings highlight the need for individualized return-to-work practices, workplace education to reduce stigma, and enhanced health benefits. Implementing supportive policies and system-level interventions is critical to fostering inclusive, equitable, and sustainable employment for individuals living with, or beyond, a cancer diagnosis.

#### 2.35.3. 24: Measuring Self-Management (SM) and SM Support in Oncology: A Scoping Review to Know Where We Stand

Sitara Sharma ^1^, Jenson Price ^2^, Sarah Chehayeb ^3^ and Jennifer Brunet ^1^
^1^ University of Ottawa, Ottawa, Canada;
^2^ Trent University, Peterborough, Canada;
^3^ McGill University, Montreal, Canada.

**Background/Rationale or Objectives/Purpose**

Emphasis on self-management (SM) and SM support (SMS) is growing in oncology, yet existing SM(S) measures remain numerous and diverse. Synthesis is needed to map this landscape, identify promising tools, pinpoint development needs, and ultimately guide more informed measure selection in research/practice. Therefore, we aimed to: (1) synthesize measures used to assess patient-reported SM and healthcare provider (HCP)-delivered SMS—both central to effective cancer survivorship—and (2) identify gaps in SM(S) measurement.

**Methodology or Methods**

Following JBI scoping review methodology, four electronic databases were searched for English-language articles administering ≥ 1 SM(S) measure(s) to patients and/or HCPs in oncology. Two reviewers screened 6551 titles/abstracts and three reviewed 1166 full-texts, yielding 409 included articles. Extracted data were analyzed descriptively, and measures were categorized by SM(S) components (tasks, skills, mechanisms).

**Impact on Practice or Results**

We identified 190 SM(S) measures (54% published/peer-reviewed; 46% study author-developed), with 52 published tools appearing in ≥2 articles. Nearly all assessed SM (98%); 2% evaluated SMS. The *Patient Activation Measure* (n = 72), *Self-Efficacy for Managing Chronic Disease Scale* (n = 29), and *General Self-Efficacy Scale* (n = 22) were used most. Measures generally captured 58% (7/12) of key SM(S) components—often medical management (task), risk reduction/health maintenance (skill), and self-efficacy (mechanism). Notably, 64% of tools were designed for non-cancer populations, and many adapted/revised versions lacked validation.

**Discussion or Conclusions**

SM(S) measurement in oncology is highly variable, rarely context-specific, and insufficiently evaluates HCP-delivered SMS. Echoing the *Global Partners for SM in Cancer’s* recommendations, findings underscore the need to develop/validate high-quality, multidimensional, conceptually grounded measures to better assess SM(S) in cancer care.

#### 2.35.4. 25: The Use of Visual Methods to Understand Cancer Survivorship: Reflections and Recommendations

Sandra Houle and Roanne Thomas.
University of Ottawa, Ottawa, Canada.

**Background/Rationale or Objectives/Purpose**

In this study, we reflect on the use of visual methods within psychosocial oncology research, drawing upon experiences from two completed arts-based research projects, exploring how women live well post-treatment. The current study’s purpose is to identify possibilities and challenges associated with the use of visual methods for understanding and representing survivorship.

**Methodology or Methods**

Across the two projects, 21 participants who had completed cancer treatment took part in virtual and in-person art-making workshops focused on expressing their lived experiences of survivorship. Participants created visual artwork and engaged in recorded group discussions and individual interviews. Photographs of participants’ artwork were taken, and interviews were transcribed verbatim and analyzed. The focus of the current study is on the visual elements and methods.

**Impact on Practice or Results**

Based on our experiences, we identified several methodological strengths. For example, visual methods encouraged reflection, agency, and connection among participants. Challenges were associated with data management and the analysis of visual materials.

**Discussion or Conclusions**

Visual inquiry offers much potential for understanding survivorship while also requiring careful attention to both pragmatic and interpretive processes. We outline various strategies for researchers who wish to engage with visual methods in the future. These include attending to relational dimensions of creativity and data collection, as well as creating flexible, analytic frameworks.

#### 2.35.5. 41: Patient and Health Care Provider Perspectives on a Transdiagnostic Digital Health Intervention to Address Comorbid Mental and Physical Health Problems in Survivors of Childhood Cancer

Jorja K. Spratt ^1^, Emma Kearns ^1^, Marin Hoh ^1^, Nicole Alberts ^2^, Gregory Guilcher ^1,3^, Lindsay Jibb ^4,5^, Melanie Noel ^1^, Quynh Pham ^6^, Vinesha Ramasamy ^7^, Kathleen Reynolds ^3^, Brooke Russell ^8^, Fiona Schulte ^1,3^, Maya Stern ^7^, Jennifer Stinson ^4,5^ and Perri R. Tutelman ^1^
^1^ University of Calgary, Calgary, Canada;
^2^ Concordia University, Montreal, Canada;
^3^ Alberta Children’s Hospital, Calgary, Canada;
^4^ University of Toronto, Toronto, Canada;
^5^ The Hospital for Sick Children, Toronto, Canada;
^6^ Toronto General Hospital Research Institute, University Health Network, Toronto, Canada;
^7^ Patient Partner, Calgary, Canada;
^8^ University of Ottawa, Ottawa, Canada.

**Background/Rationale or Objectives/Purpose**

Childhood cancer survivors (CCS) commonly experience co-occurring mental (e.g., depression and anxiety) and physical (e.g., pain, fatigue) health symptoms secondary to cancer and its treatments. Resources to address these symptoms are limited and there are significant barriers to accessing care. A digital transdiagnostic intervention (TDI) that addresses two or more symptoms concurrently could help bridge this gap. The objective of this study was to identify user needs, intended use, and necessary content and structure of a digital TDI for CCS.

**Methodology or Methods**

Virtual semi-structured interviews were conducted with Canadian CCS (*n* = 18, currently 15–24 years, diagnosed between 1 and 17 years, 56% cisgender women) who were >2 years from treatment completion and healthcare providers (HCPs) (*n* = 10, 78% cisgender women) from various disciplines. The interviews were audio-recorded, transcribed verbatim, and analyzed using reflexive thematic analysis.

**Impact on Practice or Results**

Preliminary results suggest that CCS experience unmet needs related to independently managing their physical and mental health symptoms after treatment. Reported barriers included lack of supports tailored to the childhood cancer experience, geographic disparities, and cost. Most participants endorsed the development of a digital TDI to support CCS with managing symptoms after treatment. Suggestions for the TDI included accommodating varying cognitive, visual, and auditory abilities, inclusion of customizable content based on needs and demographics, and incentivizing use.

**Discussion or Conclusions**

CCS face many unmet needs related to managing their mental and physical health symptoms after treatment and which may be addressed through a digital TDI. The findings will inform the co-design of a novel digital TDI tailored to the needs of CCS.

#### 2.35.6. 43: Exploring Social Stigma Associated with Pediatric Cancers in Newfoundland and Labrador

Rasel Siddique ^1^, Georgia Skardasi ^1^, Kayla Crichton ^1^, Lisa Goodyear ^2^, Teri Stuckless ^1^, Gerard Farrell ^1^, Eric Tenkorang ^1^, Holly Etchegary ^1^ and Sevtap Savas ^1^
^1^ Memorial University of Newfoundland, St. John’s, Canada;
^2^ Janeway Children’s Hospital, St. John’s, Canada.

**Background/Rationale or Objectives/Purpose**

Many pediatric cancer survivors face challenges, including social stigma. Stigma—often developing due to misconceptions, fear, or visible treatment effects—may lead to social isolation, reduced opportunities, and impaired quality of life of survivors. Limited research in Canada, particularly in Newfoundland and Labrador (NL), has explored the stigma-related experiences of pediatric cancer survivors. This study aims to understand the experience with and impact of pediatric cancer-related stigma. It also explores the coping strategies utilized and support needs experienced by this patient population.

**Methodology or Methods**

This is a patient-oriented, qualitative, cross-sectional study. Eligible participants (ages 10–39) are recruited through hospitals, cancer organizations, social media, and community networks. Semi-structured virtual interviews explore participants’ experiences with stigma, coping mechanisms, and support needs. To date, six interviews have been done, with five interviews transcribed verbatim. Using inductive thematic analysis, preliminary themes from transcribed interviews have been identified.

**Impact on Practice or Results**

The preliminary results identified the presence of stigma experienced by pediatric cancer survivors in NL common themes in participant experiences. Once completed, the findings of this study will be critical in informing healthcare providers, policymakers, and support organizations to develop targeted psychosocial interventions and anti-stigma programs for pediatric cancer survivors.

**Discussion or Conclusions**

Our study is ongoing with additional participants expected to join the study. The final analysis will improve the current level of understanding of stigma-related experiences in people affected by pediatric cancers and may help address cancer-related stigma and its effects locally, nationally, and globally.

#### 2.35.7. 44: Supporting School and Work Transitions in Young Cancer Survivors with Complex Needs: Pre-Test of a Liaison and Support Intervention

Marco Bonanno ^1^, Julie Desrosiers ^2^, Lye-Ann Robichaud ^3^, Nadège Gendron Granger ^4^, Nathalie Labonté ^5^, Élodie Bergeron ^6^, Christine Maheu ^7^, Leandra Desjardins ^1^, Marie-Claude Charrette ^1^ and Serge Sultan ^8^
^1^ CHU Sainte-Justine, Montreal, Canada;
^2^ Lucie-Bruneau Rehabilitation Centre, Montreal, Canada;
^3^ Integrated University Health and Social Services Centre—North Island of Montreal, Montreal, Canada;
^4^ Université du Québec à Montréal, Montreal, Canada;
^5^ Marie-Victorin School Service Centre, Montreal, Canada;
^6^ Director of Family Services, Research and Partnerships, Leucan, Montreal, Canada;
^7^ McGill University, Montreal, Canada;
^8^ Université de Montréal, Montreal, Canada.

**Background/Rationale or Objectives/Purpose**

Adolescents and young adults (AYAs) cancer survivors with complex needs face challenges that hinder school and work reintegration. Despite the need for coordinated support, hospital–community connections remain limited. This study describes and evaluates a liaison/support role promoting educational and vocational integration, and identifies key strengths and challenges of adding this role to our team.

**Methodology or Methods**

Ten AYA survivors aged ≥ 14 years and one caregiver per participant will be recruited. Eligible participants are followed, or were followed within the past two years, in pediatric oncology and present late effects affecting school/work reintegration. The study is in preparation. Participants will be referred by oncology nurses or other professionals. A career/guidance counselor will deliver 6–10 in-person or online sessions, including initial assessment and final review; caregivers will attend parts of these meetings. The activity involves collaboratively setting one–three priorities. We will collect data through grids and questionnaires before, after, and six months post-intervention.

**Impact on Practice or Results**

Data collection will address six objectives: (1) describe youth/caregiver support needs; (2) assess social validity; (3) evaluate perceived post-intervention changes; (4) document the liaison process using the TIDieR checklist; (5) explore facilitators and barriers; and (6) assess autonomy with the AIR Self-Determination Scale. Quantitative data will be analyzed descriptively, and open-ended responses coded inductively.

**Discussion or Conclusions**

This pre-test will provide insights into the feasibility, relevance, and usefulness of the intervention. The findings will guide refinement of the liaison role and workflow with the clinical team. We seek feedback on the relevance and clarity of measures, and on strategies to enhance clinical impact.

#### 2.35.8. 50: Profiles of Post-Traumatic Stress and Post-Traumatic Growth in Dyads of Parents of Childhood Cancer Survivors

Vivianne Gravel ^1,2^, Katherine Péloquin ^2,3^, Maja Krajinovic ^2,3,4^, Caroline Laverdière ^2,3^, Stacey Marjerrison ^5^, Bruno Michon ^6^, Émélie Rondeau ^2^, Daniel Sinnett ^2,3^ and Serge Sultan ^1,2,3^
^1^ Department of Psychology, Université de Montréal, Montréal, Canada;
^2^ Research Centre, CHU Sainte-Justine, Montréal, Canada;
^3^ Department of Pediatrics, Université de Montréal, Montréal, Canada;
^4^ Department of Pharmacology, Université de Montréal, Montréal, Canada;
^5^ Department of Pediatrics, McMaster University Children’s Hospital, Hamilton, Canada;
^6^ Mother-Child Center, CHU de Québec-Université Laval, Québec, Canada.

**Background/Rationale or Objectives/Purpose**

Post-traumatic stress symptoms (PTSSs) and post-traumatic growth (PTG) are common phenomena in parents who have faced childhood cancer. Very few studies examined these aspects in dyads of parents. Moreover, their contributing factors are not well identified. We aimed to describe PTSS and PTG in parental dyads, explore categories of dyads and understand the role of demographic and clinical factors as contributors to PTSSs and PTG.

**Methodology or Methods**

Dyads of parents of successfully treated childhood acute lymphoblastic leukemia survivors (14 ± 5 years post-diagnosis, *n* = 223) filled out self-report questionnaires to evaluate PTSSs and PTG as part of a larger cohort follow-up in a major Québec study. Categories of dyads were explored using latent profiles analyses. Associations between demographic and clinical factors and PTSSs and PTG levels as well as categories of dyads were modeled using dyadic analyses (APIM models).

**Impact on Practice or Results**

Parents reported fewer PTSSs and more PTG than other clinical samples. A two-profile solution showed the best fit: low PTSS–moderate PTG (83%) and very low PTSS–very low PTG (17%) in both fathers and mothers. Poor APIM model–data fit was observed, but time since diagnosis and parents’ level of education were associated with variables of interest in bivariate associations.

**Discussion or Conclusions**

Despite low levels of PTSS and moderate PTG in most parents long after treatments, the results suggest original intricacies between PTSS and PTG within parental dyads, with dyads reporting the most PTSS also reporting the most PTG, as well as potential predictors to further explore. These could help identify both vulnerable and resilient dyads.

#### 2.35.9. 54: Describing the Development of the Supportive Care 2030 Movement

Margaret Fitch.
East York, East York, Canada.

**Background/Rationale or Objectives/Purpose**

Efforts are being made around the world to implement supportive care to daily practice. Yet patients, survivors and family member continue to report unmet needs. Strategies are needed at multiple levels to shift how support is provided and person-centered care is fully achieved. This presentation describes the development of the Supportive Care 2030 Movement, a visionary call to action develop under the auspices of the Multi-national Association of Supportive Care in Cancer.

**Methodology or Methods**

From September 2022 through June 2023, a modified Delphi process was used to develop and achieve consensus regarding statements about supportive care that aim to describe a preferred future in the field. Leaders of MASCC Study Groups (N = 22) participated as an Expert Panel to provide input and rankings for the first two Delphi rounds and Patient Advocates (N = 11) then examined and offered input about the resulting statements.

**Impact on Practice or Results**

Consensus (80% agreement) was attained on 13 ambition statements, with two sub-statements. The ambition statements addressed global standards for the domains of supportive care on guideline development and implementation, coordinated and individualized care, dedicated supportive oncology services, self-management, needs for screening and actions, patient education, behavioral support, financial impact minimization, comprehensive survivorship care, and timely palliative care, reflecting collaboration, coordination and team-based approach across all levels.

**Discussion or Conclusions**

The statements describe a desired state for supportive care by 2030 and can be used to inform action plans in practice, education, research and policy. Achieving these visionary ambitions for supportive care is not the sole responsibility of MASCC and will require the concerted efforts of the global community.

Questions: How relevant are these Ambitions Statements to your practice? What is needed to implement these Ambitions Statements in your setting?

#### 2.35.10. 55: An Emerging Survivor Population: Individuals Living with Metastatic Disease

Margart Fitch.
East York, East York, Canada.

**Background/Rationale or Objectives/Purpose**

With advances in treatment, an emerging population with supportive care needs are individuals living with advanced or metastatic disease. These individuals face unique challenges and can have different priorities compared to those with early-stage disease or those nearing the end-of-life. This presentation describes the development of standards and practice recommendations for this population by an Expert Panel under the auspices of MASCC and ASCO.

**Methodology or Methods**

An expert panel comprising MASCC and ASCO members was formed. Standards and recommendations relevant to the provision of quality survivorship care for people affected by advanced or metastatic cancer were developed through: (1) a systematic review of unmet supportive care needs; (2) a scoping review of cancer survivorship, supportive care, and palliative care frameworks and guidelines; and (3) an international modified Delphi consensus process.

**Impact on Practice or Results**

A systematic review involving 81 studies and a scoping review of 17 guidelines/frameworks informed initial standards and recommendations. Seventy-seven experts (including 8 people with lived experience) across 33 countries (33% low-to-middle resource countries) participated in the Delphi study and achieved ≥ 94.8% agreement for seven standards (1. Person-Centred Care; 2. Coordinated and Integrated Care; 3. Evidence-Based and Comprehensive Care; 4. Evaluated and Communicated Care; 5. Accessible and Equitable Care; 6. Sustainable and Resourced Care; 7. Research and Data-Driven Care) and ≥84.2% agreement across 45 practice recommendations.

**Discussion or Conclusions**

These MASCC/ASCO standards will support optimization of health outcomes and care experiences by providing guidance to cancer care stakeholders for consistent provision of quality survivorship care for people affected by advanced or metastatic cancer.

Questions: What barriers do you see in your setting to implementing these standards and recommendations? What would facilitate the implementation of these standards and recommendations in your setting?

#### 2.35.11. 70: Improving Access to Psychosocial Support After Breast, Lung, Colorectal, Ovarian or Cervical Cancer for Women in Remote Northwest Territories: Implementation Evaluation of the TextMeHealthy Program

Anna C Singleton ^1^, Allyson R Todd ^2^, Rebecca Raeside ^2^, Karice K Hyun ^3,4^, Rosanna Strong ^5^, Rosemary Youngblut ^5^, Pamela Monkman ^5^, Jill Christensen ^5^, Stephanie R Partridge ^2^ and Julie Redfern ^6,7^
^1^ The Daffodil Centre, University of Sydney, Sydney, Australia;
^2^ School of Nursing, The University of Sydney, Sydney, Australia;
^3^ School of Health Sciences, The University of Sydney, Sydney, Australia;
^4^ Concord Repatriation Hospital, Sydney Local Health District, Sydney, Australia;
^5^ The NWT Breast Health/Breast Cancer Action Group, Yellowknife, Canada;
^6^ Institute for Evidence-Based Health, Bond University, Gold Coast, Australia;
^7^ Faculty of Medicine and Health, University of Sydney, Sydney, Australia.

**Background/Rationale or Objectives/Purpose**

The Northwest Territories (NWTs) in Canada are entirely remote; half the population are Indigenous. In the past decade, ~270 women completed early-stage breast, lung, colorectal, ovarian, or cervical cancer treatment. After treatment, access to locally and culturally appropriate psychosocial support is limited. Text message support programs offer an accessible solution but did not exist in NWT. To address this gap, we co-designed *TextMeHealthy* with 41 NWT community members (29% were Indigenous). This pilot aimed to evaluate implementation of *TextMeHealthy* from a community-based organisation.

**Methodology or Methods**

Single-arm pilot implementation trial with 3-month follow-up. Eligible participants were adult women ≤ 10 years post-treatment for early-stage breast, lung, colorectal, ovarian or cervical cancer. ‘NWT Action Group’ recruited participants via community gatherings, letters, posters, emails, phone, and social media, directing participants to our website (e-consent provided). *TextMeHealthy*: participants selected 2–4 weekly messages about mental health, nutrition, health management, and/or healthcare system navigation. Outcomes: uptake, representativeness, fidelity, acceptability/utility, and costs. Qualitative data was analyzed using inductive thematic analysis (dual coded).

**Impact on Practice or Results**

Uptake among website visitors was high (24/28; 85%). Participants: mean age 57 ± 12 years (range 34–76), 42% Indigenous, 76% travelled ≥ 200 km to appointments. Fidelity: 957/980 messages delivered (98%), n = 0 opted-out. Feedback (n = 13): TextMeHealthy was easy-to-understand (100%), useful (77%), and supportive (69%). Themes for improvements: more content on side effects, longer duration and simpler registration process. Cost: CAD 10.72/participant/3 months.

**Discussion or Conclusions**

Implementation of *TextMeHealthy* was feasible for delivering psychosocial support to diverse women in remote NWT. Future directions include sustained delivery and translating *TextMeHealthy* into Indigenous languages.

#### 2.35.12. 95: A Multimodal Online Chronic Pain Management Program for Survivors of Breast Cancer: Evaluating the Feasibility of the, “I Can Manage Pain After Cancer” (ICM-PAC) Program

Karen Zhang ^1,2,3^, Greg Tippin ^1,4^, Som Mukherjee ^2,3^, Greg Pond ^2,3^, Silva Darrouj ^2^, Peter Ellis ^2,3^, Deb Evans ^2^, Kathleen Gallagher ^4^, Veronica Wong ^4^, Jonathan Sussman ^2,3^, Jonathan Yen ^5^ and Denise Bryant-Lukosius ^2,3,6^
^1^ Department of Psychiatry and Behavioural Neurosciences, McMaster University, Hamilton, Canada;
^2^ Juravinski Hospital and Cancer Centre, Hamilton, Canada;
^3^ Department of Oncology, McMaster University, Hamilton, Canada;
^4^ Michael G. DeGroote Pain Clinic, Hamilton, Canada;
^5^ Alan Edwards Pain Management Unit, McGill University, Montreal, Canada;
^6^ School of Nursing, McMaster University, Hamilton, Canada.

**Background/Rationale or Objectives/Purpose**

Objectives: Pain following breast cancer treatment is common and impacts survivors’ functioning and quality of life. Although the biopsychosocial model is the gold standard for pain management, its application within cancer care remains underexplored. This study evaluated the feasibility of the “I Can Manage Pain After Cancer” (ICM-PAC) program for breast cancer survivors.

**Methodology or Methods**

Sample and setting: Participants were recruited from a hospital in Southcentral Ontario. Eligibility criteria included stage I–III breast cancer, completion of surgery and chemoradiation, and bothersome pain for over three months.

Procedures: The six-session online intervention delivered pain education, physiotherapy exercises, energy conservation strategies, and cognitive behavioural techniques. Sessions were facilitated by nursing, psychology, physiotherapy, and occupational therapy. Participants completed pre and post questionnaires assessing pain, psychological outcomes, healthcare utilization and program acceptability. Feasibility was evaluated using predefined success indicators.

**Impact on Practice or Results**

All feasibility indicators were met (consent rate (62.2%), attendance rate (75%), and attrition (13%)). Participants (N = 18; mean age = 54) found the program highly acceptable (mean = 4.11/5). Pre–post analyses revealed a 1.7-point reduction in pain interference T-scores, approaching the Minimal Clinically Important Difference of 2.0. Improvements were also observed in psychological stress (t(17) = 2.28, *p* < 0.045), depression (t(17) = 3.48, *p* < 0.01), fear of recurrence (t(17) = 4.28, *p* < 0.001), and healthcare utilization (t(17) = 3.24, *p* < 0.001).

**Discussion or Conclusions**

The ICM-PAC program demonstrated strong feasibility and acceptability, with promising effects on pain and psychological outcomes. The findings support the need for a randomized controlled trial to evaluate efficacy.

#### 2.35.13. 100: Exploring the Potential Efficacy of Psychedelic-Assisted Therapy as a Treatment for Fear of Cancer Recurrence: A Literature Review

Chloe House ^1^ and Sheila Garland ^2^
^1^ University of Ottawa, Ottawa, Canada;
^2^ Memorial University of Newfoundland, St. John’s, Canada.

**Background/Rationale or Objectives/Purpose**

Psychedelic-assisted therapy (PAT) is an exciting new frontier for psychological research. While research in the field is sparse and relatively new, current findings show promise for PAT’s ability to provide positive, lasting treatment outcomes for several psychological health issues and concerns. However, though nearly 60% of 1-year cancer survivors experience clinical levels of fear of cancer recurrence (FCR), there are currently no available studies using PAT to treat FCR.

**Methodology or Methods**

Sample and Setting: Three databases were searched for papers published on the use of PAT on a sample of cancer survivors for any psychological health or wellness concerns: PubMed, PsycINFO, and EMBASE.

Procedures: A literature review was conducted to collect all available research on using PAT for cancer survivorship concerns. The data-collection methodology follows that recommended by the PRISMA 2020 checklist to ensure all relevant data is collected. Therapeutic outcomes were compared to concerns survivors report when experiencing FCR to determine treatment compatibility and research relevance.

**Impact on Practice or Results**

A total of 46 relevant articles were identified for analysis. The therapeutic outcomes identified by previous research were identified when using psychedelics for individuals experiencing a variety of psychological issues, namely depression, anxiety, and end-of-life distress. The literature indicated three trends in the outcomes of PAT for cancer survivors: (1) reduction in existential or cancer-related distress; (2) reduction in pain; (3) reduction in depression and anxiety. These findings were compared against the symptomology and associated challenges of FCR. Psychological distress, anxiety, and new or worsening pain are known symptoms of FCR, overlapping with many of the concerns that have been previously researched.

**Discussion or Conclusions**

These findings indicate that the use of psychedelics in the treatment of FCR may be a valuable route of exploration for those seeking a long-term reduction in FCR and associated psychological health concerns.

#### 2.35.14. 134: Psychosocial, Socioeconomic Factors and Neurocognitive Functioning in Pediatric Cancer Survivors

Yustine Alejandra Carruyo Soto ^1,2^, Charlotte Roy ^1,2^, Émilie Trudel ^1,2^, Laurianne Buron ^1,2^, Ariane Levesque ^1^, Serge Sultan ^1,2^, Sébastien Perreault ^1^, Hallie Coltin ^1^, Laura Janzen ^3^ and Leandra Desjardins ^1,2^
^1^ CHU Sainte-Justine Research Centre, Montreal, Canada;
^2^ Université de Montréal, Montreal, Canada;
^3^ SickKids Research Institute, Toronto, Canada.

**Background/Rationale or Objectives/Purpose**

Pediatric Cancer Survivors (PCSs) are at elevated risk for neurocognitive impairments (Compas et al., 2017); however, psychosocial and socioeconomic factors may play a protective role (Liviana Caldiroli et al., 2025; Parsons et al., 2020; Torres et al., 2021). This study examined associations between parent-reported child resilience, anxiety, depression, parent education level, medical, and demographic factors with neurocognitive performance in PCS. Participants were 97 parent–child dyads; the children were 8–16 years old, at least one year post-treatment, and recruited from Ste-Justine and SickKids Hospital.

**Methodology or Methods**

Cognitive outcomes were assessed using standardized WISC-V scores, and executive functioning was measured using the BRIEF-2 screening parent form. Anxiety, depression and resilience were measured with BASC-3 T-scores.

**Impact on Practice or Results**

PCS were, on average, 3.95 years old at diagnosis, 8.92 years post-diagnosis, and 55.7% female. Mean scores for all WISC-V indices were within the average range, and 37% of the sample had clinically elevated BRIEF-2 scores. All variables were included in linear regression analyses. Parental education and child resilience were significantly associated with neurocognitive outcomes. Parental education and parent-reported resilience were significantly associated with higher IQ, Verbal Comprehension Index and Visuo-Spatial Index (B’s ranged from 0.02 to 13.31 and *p*’s ranged from <0.001 to 0.03). No significant associations were observed for the Working Memory, Processing Speed, or Fluid Reasoning indexes (all *p*’s > 0.05).

**Discussion or Conclusions**

Future research on neurocognitive outcomes in PCS should incorporate socioeconomic measures, as these factors may exert a strong influence on neurocognitive functioning. Supporting children of parents with lower educational attainment may help mitigate cognitive difficulties in this population.

#### 2.35.15. 142: The Moderating Role of Demographic and Clinical Factors in the Effectiveness of a Group Cognitive-Behavioral Therapy for Fear of Cancer Recurrence Integrated in Routine Care

Emma Coughlan ^1,2,3^, Claudia Mc Brearty ^1,2,3^ and Josée Savard ^1,2,3^
^1^ Cancer Research Centre, Faculty of Medicine, Université Laval, Quebec, Canada;
^2^ School of Psychology, Université Laval, Quebec, Canada;
^3^ CHU de Québec, Université Laval, Quebec, Canada.

**Background/Rationale or Objectives/Purpose**

This study aimed to explore sociodemographic, clinical, and psychological moderators of the effect of a group-based cognitive–behavioral therapy (CBT) program for fear of cancer recurrence (FCR) integrated in routine care.

**Methodology or Methods**

Over the past 6 years, 75 participants took part in a group CBT for FCR (groups of 5–11 participants), offered in routine care at the CHU de Québec-Université Laval, and completed a battery of questionnaires in the context of a quality-of-care assessment. Pre- and post-intervention measures were used. The following variables were analyzed as potential moderators of effectiveness: age, biological sex, education level, employment, cancer type, past cancer recurrence, past and ongoing treatments, and baseline anxiety, depression, insomnia, fatigue symptoms, and quality of life.

**Impact on Practice or Results**

Most participants were middle-aged women with post-secondary education. Breast cancer was the most frequent diagnosis. FCR severity decreased significantly from pre- to post-treatment. Only employment (greater reductions in FCR scores observed for retired and employed participants vs. “other” category), treatment status (greater FCR reduction for participants undergoing radiotherapy and for participants having completed hormone therapy), and quality of life (greater FCR reduction for participants having a greater quality of life) were significant moderators of treatment effects.

**Discussion or Conclusions**

These findings further support the effectiveness of this group-based therapy, with treatment outcomes remaining largely consistent across sociodemographic, clinical, and baseline psychological characteristics. Although this study has certain limitations, it contributes to the growing evidence suggesting that structured CBT interventions represent a promising approach for managing FCR within routine cancer care.

#### 2.35.16. 144: Psychosocial and Functional Drivers of Quality of Life in Cancer-Related Lymphedema: Preliminary Findings

Georgina Cama ^1,2^, Genevieve Chaput ^3,4^, Samara Perez ^1,2,4^ and Anna Towers ^3,4^
^1^ McGill University Health Centre, Montreal, Canada;
^2^ Research Institute of the McGill University Health Centre, Montreal, Canada;
^3^ Department of Family Medicine, Secondary Care and Oncology Departments, McGill University Health Centre, Montreal, Canada;
^4^ McGill University, Montreal, Canada.

**Background/Rationale or Objectives/Purpose**

This study investigates how functional and psychosocial domains impact Quality of Life (QoL) in upper-limb (UL) and lower-limb (LL) cancer-related lymphedema (CRL) patients. While clinical focus often prioritizes limb swelling, psychosocial factors significantly influence long-term outcomes and the lifelong self-management required for this chronic and incurable condition.

**Methodology or Methods**

A retrospective chart review included 507 patients (n = 235 UL; n = 272 LL) treated from January 2019 to December 2020, in a specialized lymphedema clinic in Montreal, Canada.

The validated LYMQOL tool assessed four domains: Function, Appearance, Symptom, and Emotion. Pearson correlation and multiple linear regression were utilized to identify independent predictors of overall QoL for each cohort.

**Impact on Practice or Results**

Findings indicate divergent psychosocial drivers. For UL patients, QoL was independently predicted by function (β = −0.73, *p* < 0.001) and appearance (β = −0.62, *p* < 0.001). Conversely, LL patients’ QoL was driven by function (β = −1.02, *p* < 0.001) and emotion (β = −0.87, *p* < 0.001). In both cohorts, physical symptoms and emotion were strongly correlated (r ≈ 0.63, *p* < 0.001), suggesting physical symptoms are inextricably linked to psychological suffering in CRL.

**Discussion or Conclusions**

CRL imposes limb-specific psychosocial burdens: UL patients appear uniquely impacted by body image concerns, whereas LL patients report higher distress related to mobility. These results highlight the need for limb-specific psychosocial interventions.

How can psychosocial support be integrated into CRL care models that traditionally focus on volume reduction?

Which assessment tools support physicians and lymphedema therapists identify and address the psychosocial impact of CRL?

#### 2.35.17. 148: Insights from the TrueNorth Sexual Health and Rehabilitation eTraining Program to Advance Prostate Cancer Care

Dalia Peres ^1^, Taylor Incze ^1^, Steven Guirguis ^1^, Kellie Paich ^2^, Deborah McLeod ^3^, John Robinson ^4^, Lauren Walker ^4^, Richard Wassersug ^5^ and Andrew Matthew ^1,6^
^1^ Department of Surgical Oncology, Princess Margaret Cancer Centre, University Health Network, Toronto, Canada;
^2^ Clinical Quality and Survivorship, Movember Foundation, Culver City, USA;
^3^ School of Nursing, Dalhousie University, Halifax, Canada;
^4^ Department of Oncology, University of Calgary, Calgary, Canada;
^5^ Cellular and Physiological Sciences, University of British Columbia, Vancouver, Canada;
^6^ Department of Supportive Care, Princess Margaret Cancer Centre, University Health Network, Toronto, Canada.

**Background/Rationale or Objectives/Purpose**

To date, the TrueNorth Sexual Health and Rehabilitation eTraining (SHAReTraining) program has trained 146 healthcare providers, equipping them with the knowledge, skills and confidence to address the biopsychosocial needs of patients with prostate cancer (PCa) and their partners. As the program expands internationally, ongoing systematic evaluation is required to ensure clinical relevance and sustained learner engagement. This study examined participant experiences to identify key elements underpinning SHAReTraining’s success and areas for improvement.

**Methodology or Methods**

We conducted semi-structured interviews, exploring participants’ experiences, perceptions of content, delivery, and learning processes. Interviews were audio-recorded, transcribed verbatim, and anonymized. Data were analyzed using thematic content analysis to identify recurring ideas and shared perspectives.

**Impact on Practice or Results**

Participants included nine recent SHAReTraining graduates (clinical psychology PhD students, radiation therapists, social workers) and four former SHAReTraining facilitators who are experts in the field. Participants described SHAReTraining as an informative and confidence-building program for addressing the sexual health needs of PCa patients. Key strengths of SHAReTraining included the co-facilitation model and the impact of a multidisciplinary cohort. Opportunities for enhancement focused on expanding cultural and relational content and increasing practical application.

**Discussion or Conclusions**

Identifying SHAReTraining’s core strengths and areas for refinement will support program optimization while preserving essential elements as the training scales internationally and adapts to diverse applications and sociocultural contexts.

### 2.36. Final Category: P. Other

#### 2.36.1. 13: Adapting a Group Cognitive–Behavioural Therapy for Fear of Cancer Recurrence for Women with a BRCA1/2 Genetic Mutation: A Modified Delphi Study

Emma Coughlan ^1,2,3^ and Josée Savard ^1,2,3^
^1^ Université Laval, Québec, Canada;
^2^ CHU de Québec-Université Laval Research Center, Québec, Canada;
^3^ Université Laval Cancer Research, Québec, Canada.

**Background/Rationale or Objectives/Purpose**

Fear of cancer recurrence (FCR) is particularly common among women carrying a *BRCA1/2* genetic mutation, and as such, they have expressed a clear need for psychological interventions tailored to their specific concerns (Michel et al., 2024; Savard et al., 2024). To address this issue, this modified Delphi study was aimed at identifying the necessary adaptations to the FCR group therapy currently offered as part of routine care at the CHU de Québec-Université Laval in order to better meet these women’s needs.

**Methodology or Methods**

A committee of seven experts was convened, including two patient partners, two psycho-oncologists, one hemato-oncologist, one epidemiologist, and one researcher with expertise in genetic cancers.

**Procedures:** The expert committee first reviewed a selection of the literature and existing FCR treatment protocols. A two-hour online discussion led to the development of a questionnaire listing 24 potential protocol modifications, which were administered in two rounds. Participants had to rate the relevance of each proposed change using a Likert scale ranging from “not at all relevant” to “very relevant.”

**Impact on Practice or Results**

The relevance of the items was assessed using median scores, while consensus was determined based on the difference between the interquartile range (IQR) and the adjusted IQR. At the end of the two rounds, 79% of the items achieved consensus. Among the proposed changes, 37% were deemed “highly relevant,” 21% “moderately relevant,” 26% “somewhat relevant,” and 16% “not at all relevant.” A total of 11 adaptations was retained based on their clinical feasibility.

**Discussion or Conclusions**

This modified Delphi study identified key adaptations to make to the existing FCR group therapy, to better meet needs of cancer survivors with a *BRCA1/2* mutation.

1. In your view, what shapes the experience of FCR of cancer survivors with a *BRCA1/2* genetic mutation?
2. Do you see other aspects that we should have considered in adapting the treatment protocol?

#### 2.36.2. 49: Shaping the Future of Psychosocial Oncology Advocacy Through a Shared Vision and a Collective Priority Agenda

Carmen G. Loiselle ^1,2^ and Samar Attieh ^3^
^1^ Department of Oncology and Ingram School of Nursing, Faculty of Medicine and Health Sciences, McGill University, Montreal, Canada;
^2^ Segal Cancer Center, Jewish General Hospital, Montreal, Canada;
^3^ Division of Experimental Medicine, Faculty of Medicine and Health Sciences, McGill University, Montreal, Canada.

**Background/Rationale or Objectives/Purpose**

The Canadian Association of Psychosocial Oncology (CAPO) sought input from Canadian organizations advocating for psychosocial oncology (PSO) to co-design a national advocacy agenda with key action items.

**Methodology or Methods**

In an initial brief online survey, 54 Canadian organizations reported being involved in some form of PSO advocacy. Of these, 35 completed a more extensive online follow-up survey (27 items) aiming at identifying PSO advocacy priorities, strategies, and future directions. Thematic analysis was used to analyze open-ended responses and identify the main themes.

**Impact on Practice or Results**

The emerging main themes included the following: (1) converging advocacy efforts and engaging influential champions, (2) ensuring that patients and informal caregivers are represented in all advocacy initiatives put forward, (3) securing sustained advocacy funding, (4) increasing public and government awareness of the significance of psychosocial effects linked to cancer, and (5) adopting scalable strategies and measuring advocacy-related outcomes to better inform policy at local, provincial, and national levels. Respondents also underscored the importance of CAPO in leading advocacy initiatives through capacity-building, the creation of advocacy toolkits that include best practices, forging new collaborations, and prioritizing strategic partnerships.

**Discussion or Conclusions**

A shared national PSO advocacy agenda actively disseminated by CAPO will serve to promote more equitable and people-centred cancer care throughout Canada.

#### 2.36.3. 93: A Strategic Plan for Supportive Care in British Columbia: Moving from Vision to Action

Alan Bates ^1^, John-Jose Nunez ^1^, Julia Ridley ^1^, Lauren Capozzi ^2^, Nadeesha Fernando ^1^, Ben Lee ^1^, Jonathan Avery ^2^, Tammy Hoefer ^3^ and Caylee Raber ^4^
^1^ BC Cancer, Vancouver, Canada;
^2^ BC Cancer, Kelowna, Canada;
^3^ BC Cancer, Prince George, Canada;
^4^ Emily Carr University, Vancouver, Canada.

**Background/Rationale or Objectives/Purpose**

Over the past year, BC Cancer has worked to integrate strategic planning for supportive care, including psychosocial oncology, pain and symptom management/palliative care, and cancer rehabilitation into British Columbia’s broader Cancer Plan. It has been recognized that Supportive Care is not peripheral to acute cancer care, but rather a core element of comprehensive cancer care that must be incorporated into the design of the wider interdisciplinary care team.

**Methodology or Methods**

Supported by the BC Cancer Executive, BC Cancer Patient Engagement, and the Emily Carr Health Design Lab, BC Cancer Supportive Care has conducted strategic planning over the past year including an environmental scan of supportive care services at other leading cancer centres, workshops with supportive care staff, operations leaders, professional practice leaders, Indigenous Health, patient partners, and others, input from clinicians who refer to Supportive Care, survey data from over 500 patients and caregivers, and lessons from virtual and in-person patient and caregiver workshops.

**Impact on Practice or Results**

Key products from the strategic planning include a health human resources plan outlining staffing needs across all supportive care disciplines (e.g., counselling, psychiatry), a 3-year roadmap outlining tangible actions needed to achieve our goals, an increased awareness and understanding of supportive care across BC Cancer, and a strengthened partnership with patients, caregivers and the broader cancer care community.

**Discussion or Conclusions**

In the discussion portion of the session, we will also describe the significant challenges encountered and hope to receive audience feedback on our human resource planning and on the order of priorities set out in our 3-year roadmap.

#### 2.36.4. 114: Advocacy: Influencing Change from the Personal to the Structural

Laura Burnett ^1^ and Stephen Piazza ^2^
^1^ Canadian Cancer Society, Hamilton, Canada;
^2^ Canadian Cancer Society, Toronto, Canada.

**Background/Rationale or Objectives/Purpose**

Psychosocial oncology professionals support patients and caregivers through direct advocacy, empowering individuals to navigate complex health systems. This 90-min interactive workshop will invite professionals to explore how these direct advocacy practices can be scaled up to influence structural change at federal, provincial, and health system levels.

**Methodology or Methods**

Building on the foundational advocacy skills, this workshop will examine how frontline insights and professional expertise can be leveraged to shape policy, improve system responsiveness, and address systemic barriers to care. Participants will engage with real-world examples, reflect on their own advocacy experiences, and collaborate to identify opportunities for collective action.

**Impact on Practice or Results**

Workshop participants will explore advocacy that integrates the specialist knowledge of psychosocial oncology professionals with the lived experiences of patients and/or best practices in advocacy. By blending these perspectives, we aim to create strategies that are both grounded and impactful.

**Discussion or Conclusions**

Whether participants are new to advocacy or already engaged in systemic efforts, this session will provide tools, inspiration, and a shared space to envision enhancements in the cancer care system. Join us to help continue to strengthen advocacy in oncology, where individual voices come together to influence change.

